# Evidence for common horizontal transmission of *Wolbachia* among butterflies and moths

**DOI:** 10.1186/s12862-016-0660-x

**Published:** 2016-05-27

**Authors:** Muhammad Z. Ahmed, Jesse W. Breinholt, Akito Y. Kawahara

**Affiliations:** Florida Museum of Natural History, University of Florida, Gainesville, FL 32611 USA

**Keywords:** Butterfly, Genome, Lateral gene transfer, MLST strains, Moth, Symbiont, Transmission route

## Abstract

**Background:**

*Wolbachia* is one of the most widespread bacteria on Earth. Previous research on *Wolbachia*-host interactions indicates that the bacterium is typically transferred vertically, from mother to offspring, through the egg cytoplasm. Although horizontal transmission of *Wolbachia* from one species to another is reported to be common in arthropods, limited direct ecological evidence is available. In this study, we examine horizontal transmission of *Wolbachia* using a multilocus sequence typing (MLST) strains dataset and used *Wolbachia* and Lepidoptera genomes to search for evidence for lateral gene transfer (LGT) in Lepidoptera, one of the most diverse cosmopolitan insect orders. We constructed a phylogeny of arthropod-associated MLST *Wolbachia* strains and calibrated the age of *Wolbachia* strains associated with lepidopteran species.

**Results:**

Our results reveal inter-specific, inter-generic, inter-familial, and inter-ordinal horizontal transmission of *Wolbachia* strains, without discernible geographic patterns. We found at least seven probable cases of horizontal transmission among 31 species within Lepidoptera and between Lepidoptera and other arthropod hosts. The divergence time analysis revealed that *Wolbachia* is recently (22.6–4.7 mya, 95 % HPD) introduced in Lepidoptera. Analysis of nine Lepidoptera genomes (*Bombyx mori*, *Danaus plexippus, Heliconius melpomene*, *Manduca sexta*, *Melitaea cinxia*, *Papilio glaucus*, *P. polytes*, *P. xuthus* and *Plutella xylostella*) yielded one possible instance of *Wolbachia* LGT.

**Conclusions:**

Our results provide evidence of high incidence of identical and multiple strains of *Wolbachia* among butterflies and moths, adding Lepidoptera to the growing body of evidence for common horizontal transmission of *Wolbachia*. This study demonstrates interesting dynamics of this remarkable and influential microorganism.

**Electronic supplementary material:**

The online version of this article (doi:10.1186/s12862-016-0660-x) contains supplementary material, which is available to authorized users.

## Background

Offspring vertically inherit both nuclear and non-nuclear genetic material from their mothers [1]. Among the non-nuclear material inherited are intracellular bacteria which are transferred vertically from mother to offspring and often live in symbioses with their hosts [2]. These symbionts may be obligate (essential for host survival) or facultative, in which case they can increase or decrease host fitness [3, 4]. Obligate symbionts are found within specialized cells and typically share a long evolutionary history with their hosts [5], whereas facultative symbionts tend to have more recently formed host associations. *Wolbachia* (Alphaproteobacteria: Rickettsiales: Rickettsiaceae) is a genus of facultative endosymbiont common among arthropods that is estimated to have infected more than half of arthropod species [6], including two-thirds of all extant insect species [7]. As with other facultative endosymbionts, *Wolbachia* has been thought to primarily undergo vertical transmission from mother to offspring with high fidelity [5]. However, symbionts can also develop host associations via horizontal transmission between different host species [2, 4, 8]. Horizontal transmission is thought to be the most likely explanation for closely related symbionts occurring in phylogenetically distant insect lineages [2, 8–13]. There have been multiple phylogenetic and transinfection studies reporting evidence of *Wolbachia* transmission between both phylogenetically close and distant hosts [9, 14–18]; it is therefore probable that horizontal transmission of *Wolbachia* is occurring between some arthropod taxa [4].

Butterflies and moths (Lepidoptera) constitute one of the most diverse insect orders with nearly 158,000 described species [19]. Lepidoptera play an important role in ecosystems and serve primarily as pollinators and herbivores, though some species feed on blood and other animal secretions [20–23]. The order includes many significant agricultural pests, and some species serve as models for many biological disciplines [24]. Furthermore, lepidopteran larvae are hosts to other major insect radiations – the parasitic flies and wasps [25–27]. Despite the diversity of Lepidoptera and their many associations with other organisms, little is known about their bacterial community.

*Wolbachia* are some of the most widespread endosymbiotic microbes [6, 28–30]. In nematodes, *Wolbachia* interact mutually [28], and in arthropods, *Wolbachia* most commonly interact with their hosts via a parasitic manipulation of the reproductive system [28]. Consequently, *Wolbachia* has been thought to undergo vertical transmission much more frequently than horizontal transmission [28]. *Wolbachia* most commonly affects Lepidoptera via reproductive manipulation and can induce multiple phenotypes including feminization, male killing, and cytoplasmic incompatibility [31–33]. One strain of *Wolbachia* enhances the susceptibility of its lepidopteran host to baculovirus, rendering it a potential biological control agent against the agricultural pest *Spodoptera exempta* [34]. It was recently estimated that approximately 80 % of Lepidoptera species are infected with *Wolbachia* [29], a prediction that is considerably higher than the 52 % estimated infection frequency across arthropods [6]. However, the reported mean prevalence (27 %) in Lepidoptera [29] does not significantly differ from the estimated prevalence in arthropods (24 %) [6]. The high incidence and low prevalence may reflect opportunities for substantial horizontal transfer of *Wolbachia* in Lepidoptera.

After *Wolbachia* undergoes stable horizontal transmission from natural to novel hosts, there are multiple possible phenotypic effects. We define “phenotype” as the set of observable characteristics of host result from its interaction with *Wolbachia.* The *Wolbachia* phenotype can become stronger, weaker, or remain the same, and in some cases, it can be changed to an unknown phenotype that is novel to the host [35]. Additionally, once *Wolbachia* has successfully established a close relationship with its novel host, it may transfer a gene from its genome to the host genome over time [28]. This is known as lateral gene transfer (LGT) [36, 37], and LGT is thought to be responsible for the presence of *Wolbachia* genes in 70 % of arthropod and nematode genomes [36, 38, 39]. A recent study showed evidence of ancient LGT of *Enterococcus* bacteria in Lepidoptera [40].

In this study, we 1) analyzed all published multilocus sequence typing strains (MLST) of *Wolbachia* including those from lepidopteran hosts in order to explore potential instances of horizontal transmission events, 2) surveyed transinfection experiments in Lepidoptera, to detail the factors underlying the host phenotype after horizontal transmission has occurred, and 3) searched for evidence of LGT between *Wolbachia* and Lepidoptera genomes. Our analyses reflect the complex dynamics of transmission between *Wolbachia* and their lepidopteran hosts.

## Methods

### Data collection

We used multilocus sequence typing (MLST) strains based on five loci to identify and explore *Wolbachia* strain diversity. MLST provides a universal and unambiguous tool for strain typing, population genetics, and molecular evolutionary studies [41]. MLST was developed as a universal genotyping tool for *Wolbachia* and was found effective for detecting diversity among strains within a single host species, as well as for identifying closely related strains found in different arthropod hosts [41]. We downloaded and analyzed all 345 publically available strains of *Wolbachia* in arthropods and nematodes on March 31, 2014 from the PubMLST website (http://pubmlst.org/Wolbachia/) developed by Jolley and Maiden [42] (Additional file 1: Table S1). Approximately 26 % of these strains (90/345) were associated with lepidopteran hosts: 81 were strictly found in lepidopteran hosts whereas nine strains were found in both lepidopteran and non-lepidopteran arthropod hosts (Additional file 2: Table S2). Some of the strains from lepidopteran hosts (16/90) were unnamed and incomplete because not all five of the MLST loci were sequenced (*gatB, coxA hcpA, ftsZ,* and *fbpA*); these strains were designated as unassigned (UA) strains (Additional file 3: Table S3), and we included them in our analysis as such.

### Sequence alignment and datasets

For ingroups, we included 345 MLST strains based on five MLST loci (*gatB, coxA hcpA, ftsZ,* and *fbpA*) of *Wolbachia*. For outgroups, we included bacteria closely related to *Wolbachia*: *Anaplasma marginale* (NCBI Genome accession no. NC_022760), *Ehrlichia ruminantium* (NC_006831) and *Rickettsia slovaca* (NC_017065), and extracted the five MLST loci from these genomes. These three outgroups and 345 ingroups were downloaded and aligned with the GINS-I algorithm in MAFFT [43]. Geneious v8 [44] was used to trim, align, and concatenated the five MLST loci. The best model and partitioning scheme were chosen using the Bayesian Information Criterion (BIC) in PartitionFinder v1.0.1 [45] and resulted in two partitions (a combined first and second codon position [nt12]; and third codon positions only [nt3]).

### Phylogenetic analysis

Maximum likelihood (ML) phylogenetic analyses were conducted in RAxML v8 [46] using a GTR + G model for each partition. To estimate the best ML tree in RAxML, we used the “–f a” option to estimate 1000 bootstraps and perform a likelihood search, as well as 200 “–f d” searches that started from a randomly generated parsimony tree, following the general methods of Kawahara et al. [47]. We also estimated SH-like branch support [48] for the best topology in RAxML v8. We used the same method to construct a second ML tree for a smaller dataset of 51 strains found only in lepidopteran hosts, using three different outgroups: ID 37 from Supergroup D (host *Brugia malayi*, Nematoda), ID 505 from Supergroup C (host *Onchocerca cervipedis*, Nematoda) and ID 260 from Supergroup F (host *Odontotermes horni*, Isoptera).

A phylogeny of *Wolbachia* strains was also inferred with ClonalFrame v1.2 [49] without outgroups. ClonalFrame uses information of substitution as well as recombination events and is therefore suitable to reconstruct bacterial evolution based on multilocus data [49]. We performed ten separate runs, each with a burnin set to 250,000 generations and a sampling period of 750,000 generations, with a sampling frequency of 100. We chose the two runs with the highest mean log likelihood values and compared these to assess convergence of chains using the methods of Gelman and Rubin [50]. Trees of the posterior samples of the converged runs were then combined to compute a majority rule consensus. We also calculated the ratio of nucleotides to point mutations (r/m).

### Gene networks

Statistical parsimony network analysis has been shown to be useful for assessing species-level delimitation and to identify breaks in network connectivity [51–53]. Here we designated *Wolbachia* breaks in the network connectivity as identifying strains belonging to the *Wolbachia* species [54, 55]. In the present study, 90 strains were analyzed using a parsimony network approach [56] with TCS v.1.21 [57] using a 95 % cut-off [51]. The resulting networks identify both the relationships between the different haplotypes and the number of substitutions among connecting haplotypes [58].

### Mantel test

A Mantel test was used to compute the Pearson correlation coefficient R using XLSTAT 2014 (http://www.xlstat.com). The test was performed on the pairwise node distance matrix of lepidopteran families from Regier et al.’s lepidopteran tree [59] and *Wolbachia* strains to test for significant association between matrices [60, 61].

### Co-phylogenetic analysis

*Wolbachia* strains from eight families of Lepidoptera were tested for codivergence.

We mapped the *Wolbachia* ClonalFrame tree onto the Lepidoptera phylogeny of Regier et al. [59] using JANE v4 [62]. We reconstructed codivergence patterns with default cost values for cospeciation (0), duplication (1), duplication and host switch (2), loss (1), and failure to diverge (1). JANE analysis was performed using 500 generations and population sizes of 100. We selected an edge-based cost model and a node cost model; these models differ in counting the number events related to cospeciation, duplication and failure to diverge.

### Divergence time estimation

To compare the age of *Wolbachia* divergence to previously published Lepidoptera divergence time estimations, we dated the splits of all *Wolbachia* strains found in lepidopteran species. Divergence time estimation analyses were performed in BEAST v2.1.3 [63] and two independent calibrations were used to cross-validate our estimates [64]. We applied the following calibration approaches: 1) using a recently published evolutionary rate of *Wolbachia*, estimated from *Wolbachia* genomes [65] and 2) using the age of a monophyletic set of strains shown to have strictly cospeciated with their hosts (bees) [66]. We tested for the presence of a strict clock for nt12 and nt3 datasets using a likelihood ratio test (LRT) [67] in PAUP* v4.0 [68]. Since the LRT test can be affected by recombination, we also used the relative-rate test (RRT) of Posada [69] in HyPHY [70], which can discriminate between strict and relaxed clock models in the presence of recombination. Because RRT requires that the outgroup taxa are recombination free, we used 3SEQ [71], implementing the full run mode for each gene to assure that the outgroup taxa did not have any recombinant genes. RRT analyses included taxa with unique sequences and no missing MLST loci and used two different outgroup MLST strains (13_Ekue_A_Ephestia_Pyralidae, 22_Aenc_B_Ugardan_Acraea_Nymphalidae). For the RRT, an alpha of ≤ 0.05 with a Bonferroni correction was treated as significant, and if any test was significant, then the strict clock is rejected [56]. Since both the LRT and RRT rejected the strict clock, we estimated divergence times using a relaxed lognormal clock and applied one of the two calibrations to cross-validate estimates.

The first calibration scheme was based on the median rate (substitutions per site per generation) of the *Wolbachia* genome [65] reported in generations of *Drosophila melanogaster*, which was converted to year (10 generations per year) and scaled the rate to substitutions per site per million years (nt12 was 6.42× 10^−3^ [2.76 × 10^−3^ -1.29× 10^−2^, 95 % HPD] and nt3 was 6.87× 10^−3^ [2.88 × 10^−3^ -1.29× 10^−2^, 95 % HPD]). We set lognormal priors that spanned the 95 % HPD of the previous rate estimations (for nt12: lognormal M = 0.00642 and S = 0.45; for nt3: M = 0.00687 and S = 0.44). The second calibration scheme was based on the divergence time of MLST *Wolbachia* strains (*w*NLeu, *w*Fla, *w*NPan) from Gerth et al. [66]. The MRCA of these MLST strains is estimated at 1.7 mya (0.86–2.61, 95 % HPD) [72]. We included these three strains in our divergence time analysis and calibrated the age of this group with a lognormal prior set to span the estimated HPD (M = 1.6 S = 0.33).

For each calibration scheme, we ran two BEAST analyses for a total of 4 runs using default settings for the remaining priors. We ran the MCMC chains for 150,000,000 generations, sampling every 1000^th^ generation, and used Tracer [73] to ensure that the runs converged and had ESS values >200. For comparison with *Wolbachia* divergences, we applied the published divergence times of lepidopteran families [74, 75].

### Evidence of LGT

MUMmer [76] was used to align *Wolbachia* and Lepidoptera genomes to search for evidence of LGT events. We used the following nine *Wolbachia* genomes: *w*Bm (D) (host: Nematoda: *Brugia malayi*; AE017321) [77], *w*Bol (B) (Lepidoptera: Nymphalidae: *Hypolimnas bolina*; CAOH01000001-CAOH0100014) [78], *w*Mel (A) (Diptera: Drosophilidae: *Drosophila melanogaster*; NC_002978) [79], *w*Pip (B) (Diptera: Culicidae: *Culex quinquefasciatus*; NC_010981) [80], *w*Ri (A) (Diptera: Drosophilidae: *Drosophila simulans*; NC_012416) [81], *w*Alb (B) (Diptera: Culicidae: *Aedes albopictus*; CAGB01000001-CAGB01000165) [82], *w*Vit (B) (Hymenoptera: Pteromalidae: *Nasonia vitripennis*; AERW00000000) [83], *w*Ha (A) (Diptera: Drosophilidae: *Drosophila simulans*; CP003884) [84], and *w*No (B) (Diptera: Drosophilidae: *Drosophila simulans*; CP003883) [84]. At the time of this study, there were nine available Lepidoptera genomes that were used to search for possible LGT events: *Bombyx mori* [85], *Danaus plexippus* [86], *Heliconius melpomene* [87], *Manduca sexta* (http://agripestbase.org/manduca), *Melitaea cinxia* [88], *Papilio glaucus* [89], *P. polytes*, *P. xuthus* [90] and *Plutella xylostella* [91].

## Results

### MLST strain diversity in Lepidoptera

All *Wolbachia* strains with known associated lepidopteran hosts were grouped in either Supergroup A or B (Additional file 2: Table S2). The majority of lepidopteran strains (76 total representing 32 unique MLST strains) belong to Supergroup B; the remaining (14 total strains representing 6 unique MLST) strains belonging to Supergroup A.

### Phylogenetic analysis of MLST strains

**Fig. 1:**
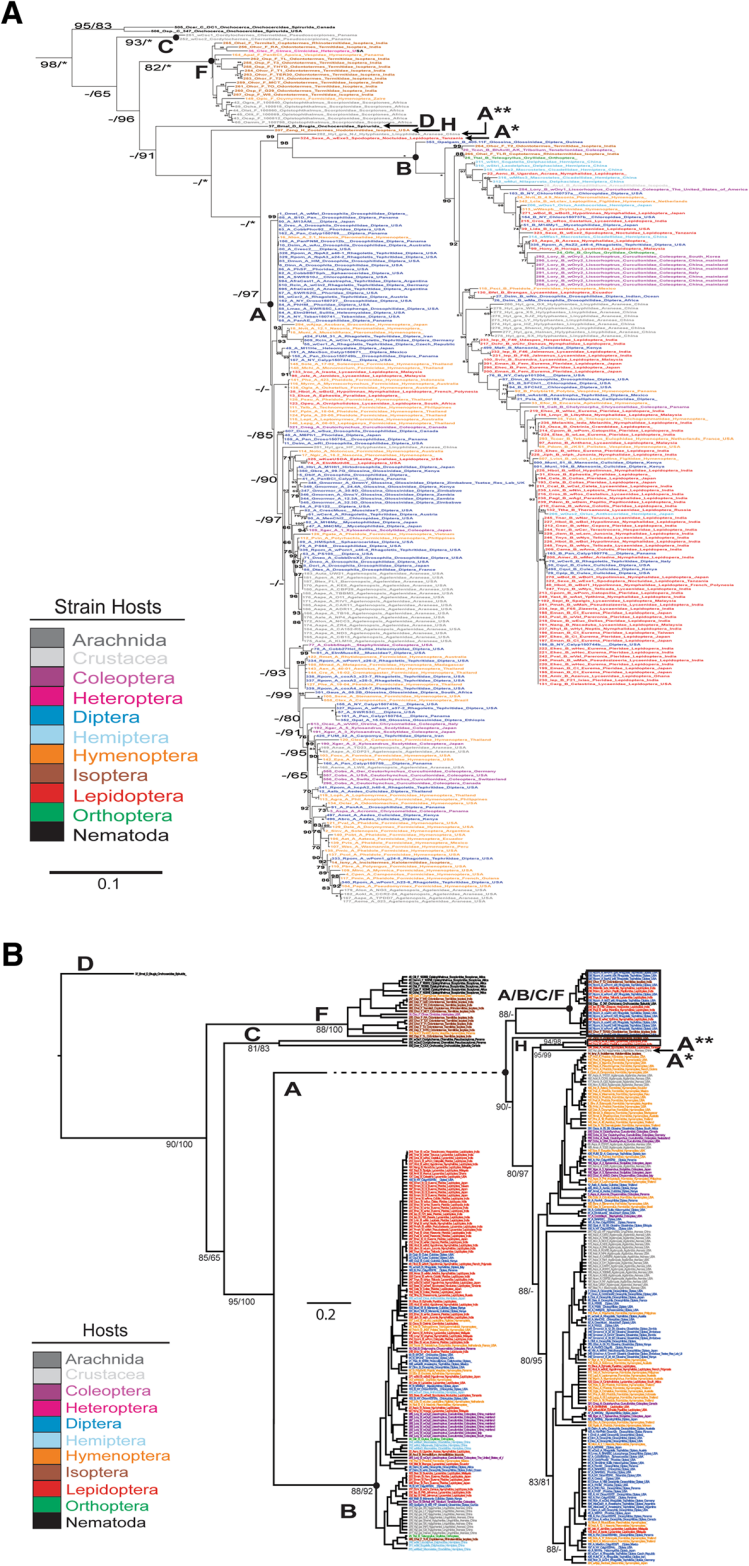
**a** Maximum likelihood (ML) tree based on the concatenated five *Wolbachia* MLST loci (2079 bp). ML boostrap values are placed to the *left* of the hyphen and SH-Like branch support values placed to the *right* of the hyphen. Bootstrap values >60 % are placed by nodes; 100 % bootstrap values indicated by an astrisks. Outgroups were removed for simplicity. A-H refer to Supergroups A-H. **b** Majority-rule ClonalFrame genealogy based on the concatenated, five *Wolbachia* MLST loci (2079 bp) from nematodes and arthropods. Labels correspond to *Wolbachia* strains and host species, families and geographic localities. Support values represent the percentage of trees from the posterior sample in which each node was present. Bootstrap values from ML analyses based on 1000 pseudoreplicates are shown

ClonalFrame and RAxML analyses both yielded similar topologies overall. The few differences in the trees might be due to recombination or difference in outgroup selection (Fig. 1a, b), and the chance of recombination is likely negligible. The ratio of nucleotide changes (from recombination) to nucleotides changes from point mutations (r/m) on average, was 1.48 (0.97–2.1, 95 % credibility region), which is considerably lower than the average (r/m = 3.5) seen in other *Wolbachia* MLST studies [92]. Some strongly supported clades in the ML analysis were also recovered in the ClonalFrame analysis of the dataset, including all currently available MLST profiles (Fig. 1a, b).

In total, 345 *Wolbachia* strains were analyzed from insect hosts (Coleoptera, Diptera, Hemiptera, Hymenoptera, Isoptera, Orthoptera, Lepidoptera) and distantly related invertebrates (Arachnida, Crustacea, and Nematoda). The ML and ClonalFrame phylogenetic trees were divided into six major clades (Supergroups A-D, F, and H). The ClonalFrame tree also contained an additional clade with strains in Supergroups A, B, C and F; this likely represents sequences that underwent the most recombination. Supergroup A is closely related to Supergroup B (Fig. 1a, b). The strain *w*Exe3, which has a lepidopteran host, was originally classified as A. However, it is basal to clade B with 98 % boostrap support in the ML tree, and it is denoted on Fig. 1 as “A*”. In addition, in the ML tree, strain *w*Hyl, which has an arachnid host, was highly supported (bootstrap = 99) as being basal to the strain *w*Exe3 (labeled “A**”, Fig. 1b). Supergroups A and B, along with A* and A**, were sister to a clade of strains previously classifed as Supergroup H, which further connects to Supergroup D and to Supergroup F. Supergroup C has high support (bootstrap = 85) as being a basal group near the outgroup (Fig. 1b). Most lepidopteran strains were classified in Supergroup B in both the ML and ClonalFrame trees (Fig. 1a). However, in the ClonalFrame tree, A* and A** were grouped in Supergroup A. In the ClonalFrame tree, Supergroup D has high support (bootstrap = 90) and is placed close to outgroups (Fig. 1a).

### Gene network analyses of unique *Wolbachia* strains in Lepidoptera

**Fig. 2:**
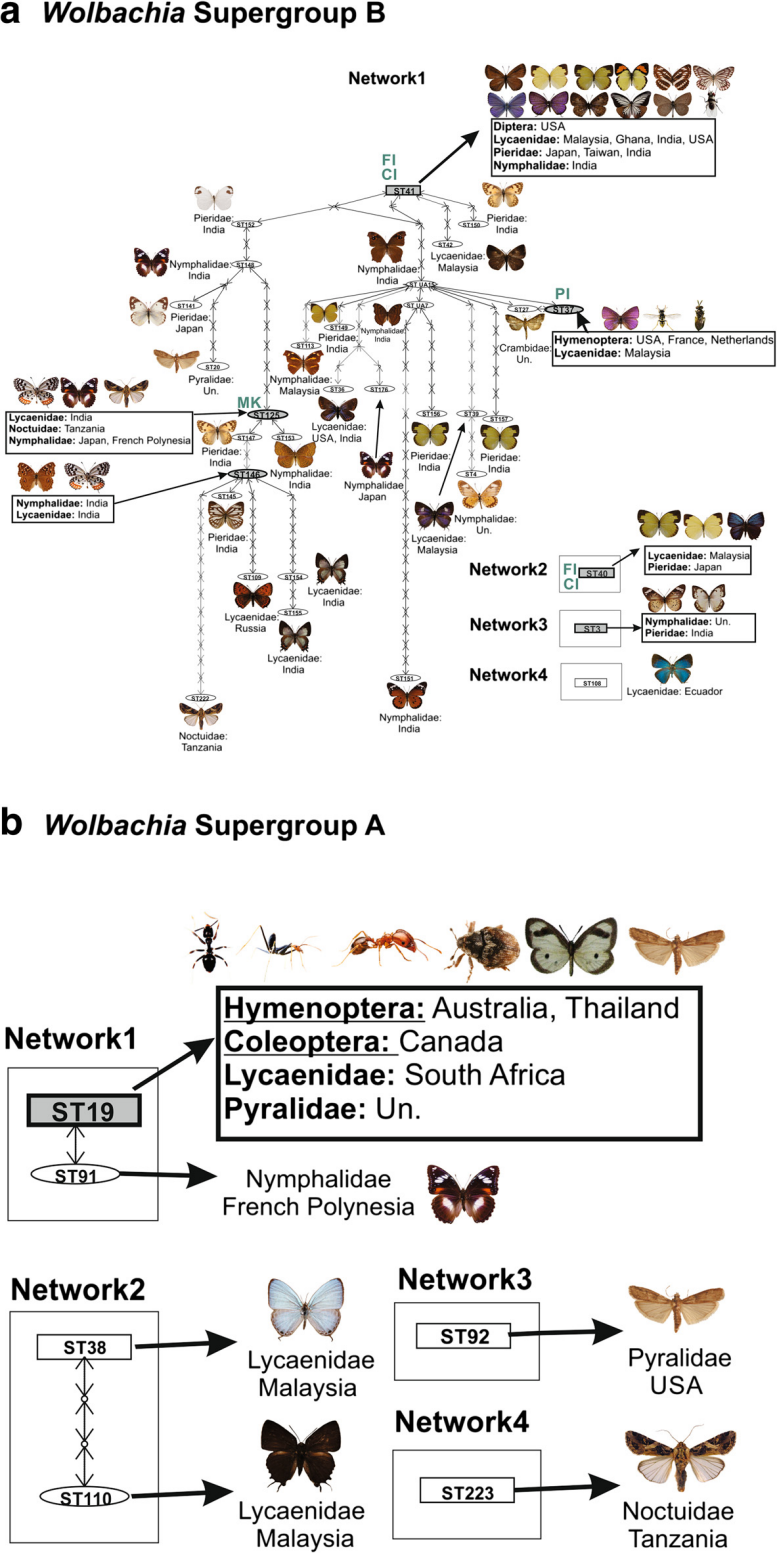
Statistical parsimony genetic network analysis (95 % confidence limit) showing genealogical relationships of *Wolbachia* strains in Lepidoptera. **a** Genetic network of *Wolbachia* Supergroup B strains in Lepidoptera. **b** Genetic network of *Wolbachia* Supergroup A strains in Lepidoptera. For (**a** and **b**), *letters in green* at the top of each strain name indicate known phenotypes for that strain; CI = Cytoplasmic Incompatibility, FI = Feminization Induction, MK = Male Killing. *Grey* indicates a strain that is inter-specific, inter-generic, inter-familial, or inter-ordinal. “Un” is used for unknown geographical locations

We performed genetic network analyses for 38 unique *Wolbachia* strains in Lepidoptera belonging to Supergroups A and B. Strains were divided into different networks based on a 95 % parsimony cut-off. Strains of Supergroup B were placed into four networks. Network 1 contained 29 strains; four of these strains were shared strains because they were found in multiple host species, and 25 strains were singletons because they were found only in single host species. These 29 strains were connected together in one network (Fig. 2a). Strain ST41 was found in 11 butterfly species (from three families) and was shared with a dipteran (Fig. 2a). Similarly, ST146 was found in two butterfly species from two different families, and ST125 was shared between two butterflies and one moth (Fig. 2a). ST37 was shared between one butterfly and two wasps: the egg parasitoid, *Tetrastichus coeruleus* (Eulophidae) and the social wasp, *Polistes dominula* (Vespidae) (Fig. 2a). Network 2 contained one shared strain, ST40, found to be present in *Eurema hecabe*, *E. mandarina,* and *Surendra vivarna*. Network 3 contained two strains from two butterflies in different families: *Acraea encedon* (Nymphalidae) and *Catopsilia pomona* (Pieridae). Network 4 contained one lepidopteran strain, found on the lycaenid butterfly *Brangas felderi* (Fig. 2a).

Strains in Supergroup A were grouped into four networks. Two networks contained only one strain: Network 3 had the lepidopteran strain ST92 (from *Ephestia kuehniella* [Pyralidae]) and Network 4 had the lepidopteran strain ST223 (from *Spodoptera exempta* [Erebidae]). Network 2 contained two strains, both with lycanenid butterfly hosts, that were separated by two mutations: ST38 (*Jamides alecto*) and ST110 (*Iraota rochana*; Fig. 2b). Network 1 contained nine strains. ST19 was found in eight strains from eight host species: *Ephestia kuehniella* (Lepidoptera: Pyralidae), *Ornipholidotos peucetia* (Lepidoptera: Lycaneidae), *Ceutorhynchus neglectus* (Coleoptera: Curculionidae), and five ant species (*Leptogenys* sp., *Leptomyrmex* sp., *Pheidole plagiara*, *P. planifrons*, *Technomyrmex albipes*). The ninth strain, ST91 occurred on the nymphalid butterfly *Hypolimnas bolina*, and was separated by just one mutation from strain ST19.

### Comparison of *Wolbachia* and Lepidoptera phylogenies

There was no strong congruence between the *Wolbachia* and lepidopteran phylogenies during mantel test. Analysis of the ML topologies for *Wolbachia* using JANE and the ML tree from Regier et al.’s [59] lepidopteran phylogeny at a *p-*value of 0.05 showed the reconstructions (cost = 92) with only 9 cospeciation events, 22 duplication, 19 duplication and host switching, 22 losses and 10 failure to diverge (Additional file 4: Figure S1).

The Mantel test analysis indicated that there were no significant correlations between the genetic distances of *Wolbachia* and host Lepidoptera (*r* = −0.072, *P* = 0.081 [indigenous]; *r* = 0.107, *P* = < 0.0001 [comparing the *Wolbachia* ClonalFrame tree with the ML tree of Regier et al. [59]]; *r* = 0.069, *P* = 0.019 [comparing the *Wolbachia* ML tree with the ML tree of Regier et al. [59]]).

**Fig. 3:**
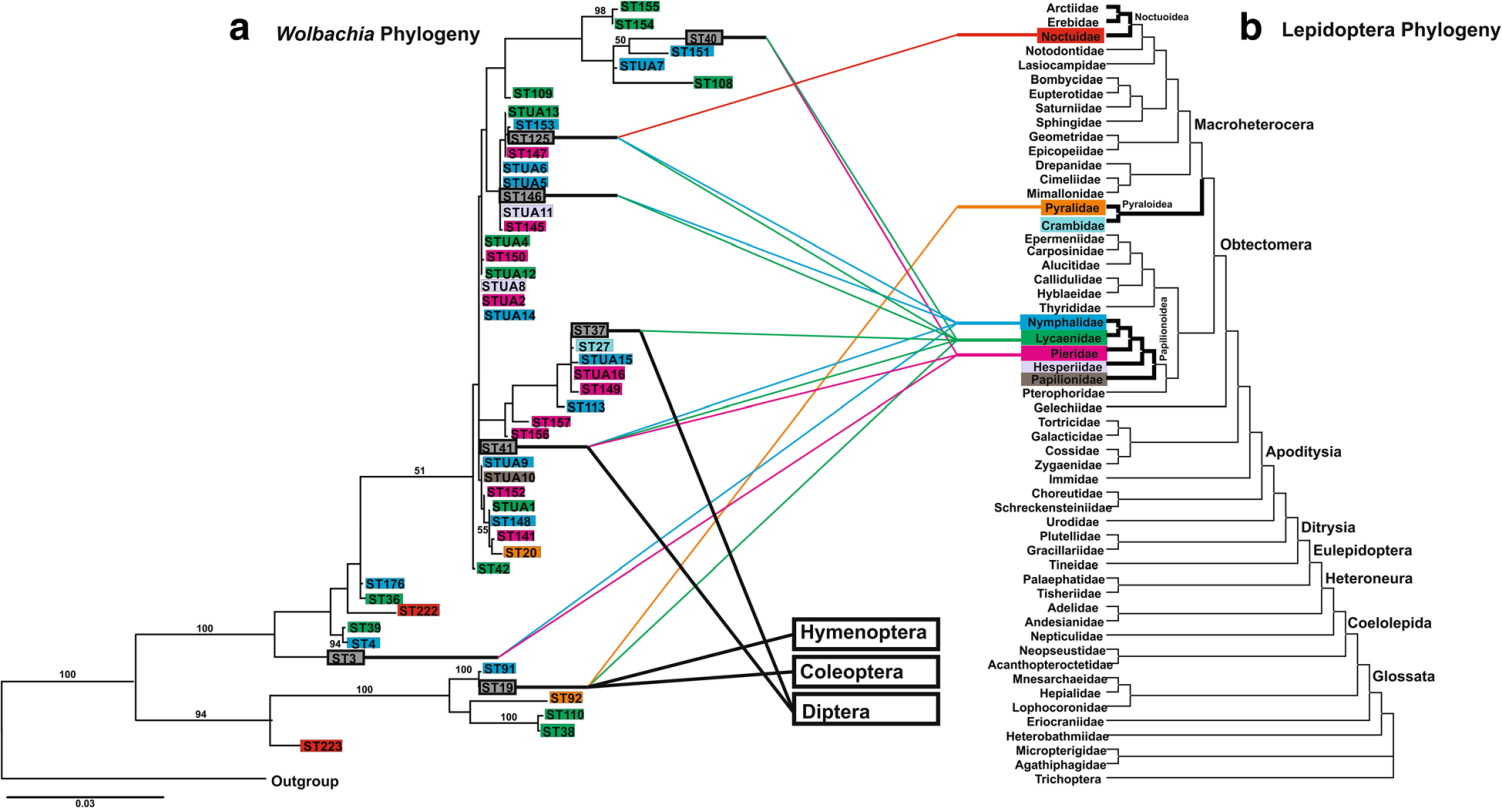
Comparison of phylogenies of *Wolbachia* their lepidopteran hosts. **a** ML tree based on the concatenated data of the five *Wolbachia* MLST loci. The tree was rooted with three strains from Supergroups C, D and F. ML bootstrap values ≥50 % shown on branches. **b** Phylogeny of Lepidoptera according to Regier et al. [59]. Colors correspond to Lepidoptera family names. *Grey* indicates a strain that is inter-familial or inter-ordinal

A phylogeny based only on unique strains of *Wolbachia* in lepidopteran hosts showed that distantly related strains were found in the same host family. Most of the *Wolbachia* strains were found in three butterfly families (Lycaenidae, Nymphalidae, Pieridae). These three were closely related [59, 93], yet they contain distantly related strains (Fig. 3). Strains ST3, ST40, ST41, and ST146 transferred horizontally across these three sister families of butterflies. Strain ST125 was found in both butterflies (Lycaenidae, Nymphalidae) and moths (Noctuidae). Strain ST19 was found in a lycaenid, pyralid, and in two non-Lepidopteran insect orders (Coleoptera, Hymenoptera), and strains ST37 and ST41 were found in multiple orders (Diptera, Lepidoptera) (Fig. 3, Additional file 4: Figure S1).

### Divergence time estimation

**Fig. 4:**
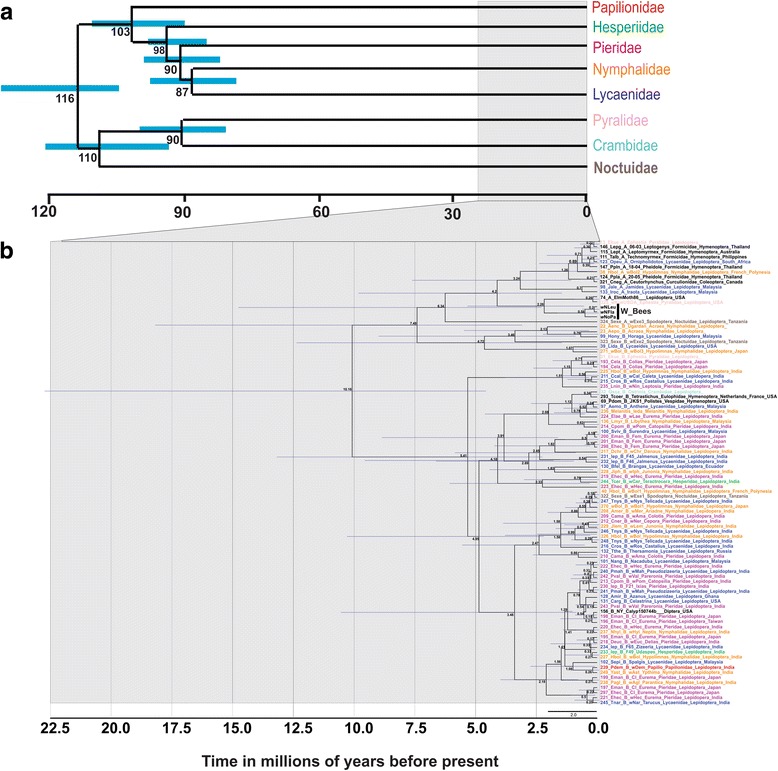
Estimated divergence times (**a**) of Lepidoptera based on Wahlberg et al. [74], and (**b**) the divergence time evolutionary rate of MLST genes [65] for *Wolbachia* Supergroups A and B Three samples (wNLeu,wNFla, wNPa) under W_Bees in (b) were taken from Gerth et al. [66] to calibrate and cross validate the divergence estimation

Both the LRT (nt12: df = 91, LRT value = 565.16, *P*-value = 0; nt3: df = 91, LRT = 1833.43, *P*-value = 0) and RRT (outgroup: *Ephestia* sp., nt12: 112/351, nt3: 140/428; outgroup: *Acraea* sp., nt12: 118/351, nt3: 272/428) rejected a strict clock. In BEAST, all run pairs converged and the ESS values were above 200. Analyses using different calibrations resulted in overlapping HPD divergence time intervals at the root with a mean of 12.67 mya (26.86–4.76 mya, 95 % HPD) using the clade calibration prior and a mean of 10.67 mya (22.6–4.7 mya, 95 % HPD) using the evolutionary rate of the *Wolbachia* as a prior. Both calibrations also provided overlapping HPDs for the age of the MRCA of (*w*NLeu, *w*Fla, *w*NPan) with the run that calibrated this clade at 0.55–1.89, 95 % HPD and the run using a rate prior at 0.0097–1.84, 95 % HPD. We compared divergence times of all lepidopteran *Wolbachia* strains (10.16–22.5-0 mya, 95 % HPD) with divergence times of lepidopteran families of Wahlberg et al. [74] that found the youngest divergence between families at 87 mya (76–98, 95 % HPD) and the oldest divergence between moths and butterflies at 116 mya (127–105 mya, 95 % HPD) (Fig. 4). In a more recent study of insect phylogenomics, the mean divergence time between butterflies and moths was much younger, estimated at ~58 mya [75] compared to 116 mya in a prior study [74]. Given either one of these Lepidoptera time estimates, if they are correct, they imply that all switches between lepidopteran families are likely to be due to horizontal transmission. Two identical *Wolbachia* strains, ST19 and ST125, between butterflies and moths are clear cases of a horizontal *Wolbachia* jump. *Wolbachia* strains ST37 and ST41 were identical in Diptera and Lepidoptera, their estimated divergence time is approximately 289.65 mya (328.62–244.11 mya, 95 % HPD) [75]. Coleoptera and Lepidoptera, with an estimated split of 326.69 mya (353.05–301.86 mya, 95 % HPD) [75], and Hymenoptera and Lepidoptera, with an estimated split of approximately 344.68 mya (372.43–317.79 mya, 95 % HPD), share the ST19 strain [75].

### Geography of shared strains

**Fig. 5:**
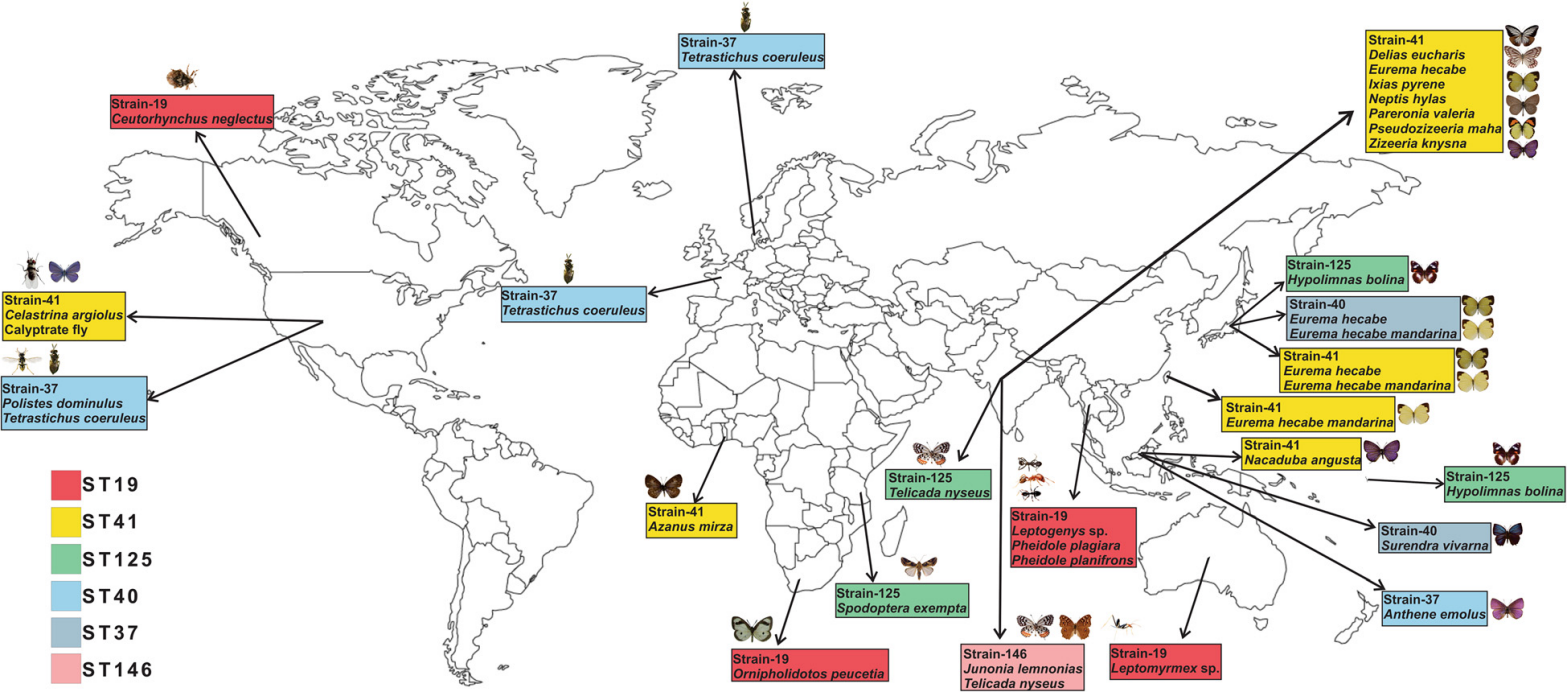
Geographical distribution of Lepidoptera-related *Wolbachia* strains. The six strains that were shared among lepidopteran and non-lepidopteran species are plotted. Each color represents one strain (Blank world map was taken from www.freeusandworldmaps.com)

Geographical distributions of six shared strains (ST19, ST37, ST40, ST41, ST125, ST146) were surveyed (Fig. 5). The seventh shared strain, ST3, was not included in this analysis due to the uncertainty of the sampling location of its host species. Strain ST41 was found in one unidentified species of calyptrate fly from the United States, and ten butterfly species from six countries: Lycaenidae: *Azanus mirza* (Ghana), *Celastrina argiolus* (United States), *Nacaduba angusta* (Malaysia), *Pseudozizeeria maha*, *Zizeeria knysna* (India); Pieridae: *Delias eucharis*, *Ixias pyrene, Pareronia valeria* (India), *Eurema hecabe* and its subspecies *E. h. mandarina* (India, Japan, Taiwan), Nymphalidae: *Neptis hylas* (India). Strain ST37 was found in one Malaysian butterfly species (*Anthene emolus*), the American wasp species *Polistes dominulus*, and the wasp *Tetrastichus coeruleus*, which was sampled in the United States, the Netherlands and France. Strain ST125 was found in a butterfly species from India (*Telicada nyseus*) and a butterfly species from French Polynesia and Japan (*Hypolimnas bolina*). ST125 was found in a butterfly species from French Polynesia and Japan (*H. bolina*) and a moth species in Tanzania (*Spodoptera exempta*). Strain ST146 was found in two different species in India (*Junonia lemnonias*, *T. nyseus*). Strain ST40 was found in one Japanese butterfly species (*E. hecabe*) and one Malaysian butterfly (*Surendra vivarna*). Strain ST19 of Supergroup A was found in four countries spanning four continents; this strain was present in one species of weevil from Canada (*Ceutorhynchus neglectus*), three species of ants from Thailand (*Leptogenys* sp., *Pheidole planifrons*, *P. plagiara*), one ant species from Australia (*Leptomyrmex* sp.) and one butterfly from South Africa (*Ornipholidotos peucetia*).

### Summary of previous transinfection studies in Lepidoptera

The horizontal transmission of *Wolbachia* can facilitate the induction of unknown phenotypes into the novel host. In the last two decades, there have been multiple transinfection studies reporting evidence of *Wolbachia* transmission between phylogenetically close and distant species [94–101]. In the present study, we surveyed previous studies on transinfection of *Wolbachia* in Lepidoptera and attempted to classify them according to the possible factors involved in the induction of phenotypes after the transinfection (Table 1). Our survey reveals that the stability of *Wolbachia* infection and induction of its phenotypes in novel hosts is determined by three factors: 1) type of strain, 2) type of host species/population, and 3) collective effects of both the host and the *Wolbachia* strain [94–101].

**Table 1:** Results of published transinfection experiments of *Wolbachia* strains performed on lepidopteran hosts

Natural Host	Strain ID	Phenotype in Natural Host	Transinfected host	Phenotype in Transinfected Host	References
1. Strain dependent Phenotype					
*Ostrinia scapulalis*	*w*Sca	MK	*E. kuehniella* (−*w*)	MK	[94, 95]
*E. kuehniella* (Yokohama)	*w*Kue	CI	*E. kuehniella* (Tsuhiura) (−*w*)	CI	[96]
2. Host dependent Phenotype					
(a) Transferable multi potent strain					
*Cadra cautella*	*w*Cau-A	CI	*E. kuehniella* (−*w*)	MK	[98]
(b) Non-transferable strain					
*Eurema hecabe*	*w*Hec	FI	*Bombyx mori* (−*w*)	no stable infection	[99]
(c) Population dependent phenotype^a^					
*Hypolimnas bolina* (Polynesia)	*w*Bol1	MK	*Hypolimnas bolina* (South Asian) (+*w*)	CI	[100, 101]
3. Strain/host dependent Phenotype					
*C. cautella*	*w*Cau-B	CI	*E. kuehniella* (−*w*)	Incomplete CI	[98]
*E. kuehniella*	*w*Kue	CI	*O. scapulalis* (−*w*)	Stronger CI	[97]

*MK* male killing, *FI* feminizaton induction, *CI* cytoplasmic incompatibility, ^a^ these observations were not based on transinfection experiments instead were based on observations in the field

### Lateral gene transfer (LGT)

We found one possible case of LGT between the *Wolbachia* strain *w*Ha of *Drosophila simulans* and the genome of the butterfly *Melitaea cinxia*. The portion of the *Wolbachia* gene found in the genome of *M. cinxia* was 350 bp with > 96 % identity. We trimmed that hit from the receptive scaffold 391 between 44,255 and 44,603 bp in the genome of *M. cinxia* and blasted and reconfirmed that it is the part of *Wolbachia* genome (between 662,982 and 663,331 bp) with 100 % query cover and > 96 % identity (337/350 bp) with a 4–160 e-value. While blasting, we found that the portion of this gene is a part of the locus *w*Ha_05420, and it is associated with a hypothetical protein (AGJ99989.1). We did not find any evidence of LGT in the other eight genomes of Lepidoptera aligned against available genomes of *Wolbachia*. However, we found four hits in *P. xylostella* ranging between 544 and 569 bp in length with 81–83 % similarity. We blasted those hits and found that they matched *Enterobacter* sp. with > 97 % identity (Table 2).

**Table 2:** Comparisons of genomes of *Wolbachia* and Lepidoptera to test for traces of LGT

Host	Total traces screened (mbp)	*Wolbachia* traces hits^a^	Hits length (bp)	Identity %	NCBI Blast matches	LGT	*Wolbachia* infection
*Plutella xylostella*	388	4	545–569	81–83	*Enterobacter* sp.	Yes^a^	Infected
*Bombyx mori*	466	0	0	0	NA	No	Unknown
*Danaus plexippus*	265	0	0	0	NA	No	Unknown
*Heliconius melpomene*	265	0	0	0	NA	No	Unknown
*Manduca sexta*	395	0	0	0	NA	No	Unknown
*Melitaea cinxia*	387	1	350	96	*Wolbachia* (*w*Ha-A)	Yes^a^	Unknown
*Papilio glaucus*	376	0	0	0	NA	No	Unknown
*Papilio polytes*	227	0	0	0	NA	No	Uninfected
*Papilio xuthus*	244	0	0	0	NA	No	Uninfected

^a^There is a possibility of LGT based on our genomes scanning results. Genomes of *Wolbachia* used in this study; *w*Bm (D), *w*Bol (B), *w*Mel (A), *w*Pip (B), *w*Ri (A), *w*Alb (B), *w*VitB, *w*Ha (A) and *w*No (B)

## Discussion

Previously, vector-mediated interspecific transmission was observed in *Wolbachia* through shared food sources [2, 102–105], ectoparasitic mites [106, 107], and parasitoids [4]. Our study revealed that inter-specific, inter-familial, and inter-ordinal horizontal transmission is also common in Lepidoptera. Using phylogenetic, co-phylogenetic and network analyses, we found at least seven probable cases of horizontal transmission among 31 host species, both within Lepidoptera and between Lepidoptera and other arthropods. Three strains (ST3, ST40, ST146) were shared among three butterfly families (Lycaenidae, Nymphalidae, Pieridae). One strain (ST125) was shared between two butterfly families (Lycaenidae, Nymphalidae), and the distantly related moth family Noctuidae. Since the majority of lepidopteran larvae feed on plant tissue, and adults obtain nectar from flowers or tree sap, the close association of Lepidoptera with plants might lead to increased infection through host plant mediation [105]. Strain 41 is the most widespread *Wolbachia* strain in butterflies; it was shared among eleven butterfly species in three families (Lycaenidae, Nymphalidae, Pieridae) and interestingly, it was also shared with one unidentified species of calyptrate fly. There are a number of known hymenopteran parasitoids that are found on both lepidopteran and dipteran hosts, and thus parasitoids may have mediated horizontal transfer [108].

Another strain, ST37, was found to be shared between the egg parasitoid *Tetrastichus coeruleus*, the social wasp *Polistes dominula*, and the lycaenid butterfly *Athene emolus. Tetrastichus coeruleus* is not known to parasitize lepidopterans. However, it parasitizes eggs of the common asparagus beetle, *Crioceris asparagi* [109], which shares a host plant with other Lepidoptera, such as the pest species *Spodoptera exigua* [110]. Perhaps *Wolbachia* was transferred into a lepidopteran host through this shared host plant. Larvae of *Polistes dominula* are parasitoids of *Chalcoela iphitalis* (Lepidoptera: Pyralidae) [111], could serve as a possible route of *Wolbachia* transfer to a lepidopteran host. The Malaysian lycaenid butterfly, *Athene emolus,* is symbiotic with the ant species *Oecophylla smaragdina*. These ants guard *A. emolus* larvae and protect them from predators and parasites [112]. We postulate that any one of these lepidopteran-hymenopteran interactions could potentially enable inter-ordinal transfer of ST37.

Strain ST19 also exhibits inter-ordinal transfer. It is shared among three different insect orders: Lepidoptera (the lycaenid butterfly *Ornipholidotos peucetia*, and the pyralid moth, *Ephestia kuehniella*), Hymenoptera (the ant species *Leptogenys* sp., *Leptomyrmex* sp., *Pheidole planifrons*, *P. plagiara*, and *Technomyrmex albipes*), and Coleoptera (the weevil *Ceutorhynchus neglectus*). Horizontal transmission of *Wolbachia* is also possible when an uninfected insect eats an infected one [113]. *Ceutorhynchus neglectus* is parasitized by multiple wasps [114]; weevils also feed on flower pollen and nectar [115]. It is thus possible that ST19 jumped across three insect orders either through shared host plants or via shared parasitoids.

The Mantel test revealed a weak correlation between genetic make-up of lepidopteran host and its endosymbiotic bacteria, *Wolbachia,* which further support horizontal transmission of *Wolbachia* within Lepidoptera. Co-phylogenetic analysis revealed common losses, duplication and host switches of *Wolbachia* strains within Lepidoptera.

We performed divergence time analyses on all available *Wolbachia* strains from Lepidoptera using two independent calibrations [65, 66]*.* Results from both calibrations cross-validate our divergence time estimates and suggest the conclusions are robust. Our analysis suggests that *Wolbachia* was recently introduced in Lepidoptera at a maximum age of ~23 mya. The *Wolbachia* divergence times, compared to the divergence times estimated by Wahlberg et al. [74], suggest lepidopteran families that are currently known to carry *Wolbachia* had already diversified before they became *Wolbachia* hosts. A recent study on insect evolution suggests the divergence between butterfly and moths and between Lepidoptera and other insect orders (Diptera, Coleoptera and Hymenoptera) took place between ~344-58 mya and the identical strains between them were acquired recently at a maximum of ~23 mya [75]. Our divergence time analysis, in light of the most comprehensive Lepidoptera calibrated phylogeny, suggests that *Wolbachia* strains ST3, ST19, ST40, ST41, ST125 and ST146, are likely inter-familial horizontal transmissions, and ST125 and ST19 are inter-superfamilial horizontal transmissions [74, 75]. We also found that ST19, ST37, ST41 are clear cases of inter-ordinal horizontal transmission. The cospeciation events predicted in the co-phylogenetic analysis seems to be invalidated, given the lepidopteran estimated divergence times of Wahlberg et al. [74].

Facultative endosymbionts have already been shown to change host fitness or biology; pea aphids (*Acyrthosiphon pisum*) have facultative symbionts that protect their hosts against entomopathogenic fungi and parasitoid wasps, ameliorate the detrimental effects of heat, and influence host plant suitability [2, 116–118]. One main consequence of horizontal transmission is induction of unknown phenotypes of *Wolbachia* into the novel host [28]. A recently discovered *Wolbachia* strain confers fitness benefits by increasing the resistance against natural pathogens in fruit flies [119]. All previously published transinfection experiments in lepidopteran hosts arrived at similar conclusions that the phenotype induction after transinfection is determined by two factors strains and the host types [94–101]. It is necessary to investigate each strain’s genotype and phenotype in its natural host, as well as other possible hosts in which it may have been transferred through shared resources. In some cases, suppressors against phenotype can lead toward loss of phenotype [100]. Therefore, some species that do not currently induce a phenotype may have done so in the past, implying that more species have had their biology affected by *Wolbachia* than previously estimated [100]. In other cases, novel hosts can suppress the *Wolbachia*-mediated phenotype and enable the appearance of hidden phenotypes [100, 101]. Together, these studies suggest that *Wolbachia* strains possess the genetic makeup to induce multiple phenotypes [28].

The spread of endosymbionts in field populations by horizontal transmission have received little attention. The mechanisms driving horizontal transmission have mostly remained unclear; even the effects induced by common cases of horizontal transmission are currently unknown [2, 3]. Since there is no way to control horizontal transmission in the field, routes of transmission must be thoroughly studied in order to investigate the genotypes and phenotypes of strains in both natural and novel hosts.

Recently, a complete copy of the *Wolbachia* genome was found within the genome of *Drosophila ananassae* and large segments were found in seven other *Drosophila* species [36]. During the original whole genome sequencing of the nematode, *Brugia malayi*, extensive levels of lateral gene transfer (LGT) were identified from its *Wolbachia* endosymbiont [36]. LGT from the *Wolbachia* genome to the nuclear genome of its eukaryotic hosts is widespread [38, 39]. In a search of sequence data archives, about 70 % of arthropods and nematodes have evidence for LGT from *Wolbachia* [36, 38, 39]. We found one instance of possible *Wolbachia* LGT between strain *w*Ha and the butterfly *Melitaea cinxia*. This result must be confirmed with PCR to rule out the possibility of a genome-sequencing error or contamination. We did not find any evidence of LGT from the *Wolbachia* genome to the other eight available genomes of Lepidoptera. Even *Plutella xylostella,* the only species known to have *Wolbachia* infection, did not yield any evidence of LGT in our analysis of its genome. For *M. cinxia*, the evidence we found of LGT transmission suggests it is or has been infected with *Wolbachia*. The method we used to search for possible LGT has previously been used effectively to trace LGT from *Wolbachia* [36] and from other bacterial species [40]. The lack of evidence of LGT also supports our inference of a recent introduction of *Wolbachia* in Lepidoptera. Though these results are sound based on current available data, they are not conclusive; future studies should examine additional genomes and methods to trace LGT in Lepidoptera. The genome assemblies of eukaryotes often filter out bacterial sequences as contaminants and there might be possibility that *Wolbachia* genes may be present in the original sequencing reads, but not in the finished genome assemblies [120]. We suggest future studies to examine the raw data read instead of assembled genomes to detect those genes, which might have filtered from the original sequencing reads.

Ahmed et al. [29] found geographic patterns in the infection status of *Wolbachia*, however, this survey did not find any such patterns in strain distribution. The study frequently found strains distributed across the continents, such as strains ST19, ST37, and ST41, which have been found in multiple hosts across Asia, Africa, Australia and North America. There is no generally accepted theory for how these strains were transferred between various hosts across continents, partially due to the difficulty in tracing the strains’ natural hosts. The comparison of phylogenies of *Wolbachia* and host Lepidoptera indicates that closely related strains have phylogenetically diverse hosts and vice versa. These examples of shared strains across distantly related families demonstrate that horizontal jumps might be result of recent acquisition of *Wolbachia*.

Currently, only eight families of Lepidoptera have published *Wolbachia* strain data. These include three moth families (Crambidae, Noctuidae, Pyralidae) five butterfly families (Hesperiidae, Lycaenidae, Nymphalidae, Papilionidae, Pieridae)that represent three Lepidoptera superfamilies (Noctuoidea, Pyraloidea, Papilionoidea), which contain about 50 % of described lepidopteran species [19]. It would be interesting to explore the *Wolbachia* strains from other butterfly and moth families, in order to get a comprehensive estimate of the full extent of *Wolbachia* diversity and mode of transmission within this order.

## Conclusions

We found evidence for several new instances of *Wolbachia* horizontal transmission in Lepidoptera. Our findings suggest that specific shared food sources and shared natural enemies are possible routes of horizontal transmission, but further studies are needed to conclusively determine these routes. We uncover evidence of *Wolbachia* inducing new phenotypes in novel hosts after horizontal transmission from natural hosts. However, *Wolbachia*-induced phenotypes have not been well studied for most natural hosts and potential novel hosts. Therefore, it is crucial to study additional *Wolbachia-*infected organisms in order to determine which species are natural hosts for each strain. It is also important to perform additional transinfection experiments to determine which species can sustain a stable infection. Data from these experiments will yield information about the phenotypes in both natural and novel hosts, revealing new insights into the mechanisms of *Wolbachia-*induced phenotypic change. Finally, further research into host genotypes should be conducted by analyzing additional genomes of potential hosts to search for the presence of inserted *Wolbachia* loci, in order to elucidate the function of these laterally transferred genes.

### Ethics

Not applicable.

### Consent to publish

Not applicable.

### Availability of data and materials

We provided the data at LabArchive (DOI: 10.6070/H48913W9).

## Additional files

Additional file 1: Table S1. All published publicly available MLST strains of *Wolbachia* used in this study (accessed March 2014 at http://pubmlst.org/Wolbachia/). (XLSX 80 kb) Additional file 2: Table S2. Description of available MLST strains of *Wolbachia* in Lepidoptera used in this study (accessed March 2014 at http://pubmlst.org/Wolbachia/). (XLSX 48 kb) Additional file 3: Table S3. Incomplete MLST strains of *Wolbachia* in Lepidoptera (accessed March 2014, at http://pubmlst.org/Wolbachia/). (XLSX 9 kb) Additional file 4: Figure S1.
*Wolbachia* ClonalFrame genealogy (in grey, not drawn to scale) based on five MLST genes mapped onto the host family phylogeny with JANE [62]. Host family phylogeny is redrawn from Regier et al. [59]. Small solid circles show duplicated strains; small open circles show co-speciation of strains; arrows show host switches; dotted lines show loss of strains and zigzag lines show failure in strain divergence. (JPG 2300 kb) Additional file 5: Tree file. Chronogram from a median consenus tree based on 10,000 trees from the posterior distbution of the rate [65] and clade calibrated [66] beast runs. (PDF 261 kb)

## References

[1] Cosmides LM, Tooby J (1981). Cytoplasmic inheritance and intragenomic conflict. J Theor Biol.

[2] Oliver KM, Degnan PH, Burke GR, Moran NA (2010). Facultative symbionts in aphids and the horizontal transfer of ecologically important traits. Annu Rev Entomol.

[3] Himler AG, Adachi-Hagimori T, Bergen JE, Kozuch A, Kelly SE, Tabashnik BE, Chiel E, Duckworth VE, Dennehy TJ, Zchori-Fein E, et al. Rapid spread of a bacterial symbiont in an invasive whitefly is driven by fitness benefits and female bias. Science. 2011;332(6026):254–6. 10.1126/science.1199410 21474763

[4] Ahmed MZ, Li S-J, Xue X, Yin X-J, Ren S-X, Jiggins FM, Greeff JM, Qiu B-L. The intracellular bacterium *Wolbachia* uses parasitoid wasps as phoretic vectors for efficient horizontal transmission. PLoS Pathog. 2015;10(2):e1004672–2. 10.1371/journal.ppat.1004672 PMC4347858 25675099

[5] Buchner P. Endosymbiosis of animals with plant microorganisms. New York: John Wiley and Sons Interscience; 1965. 332–338.

[6] Weinert LA, Araujo-Jnr EV, Ahmed MZ, Welch JJ (2015). The incidence of bacterial endosymbionts in terrestrial arthropods. Proc. R. Soc. B Biol. Sci..

[7] Hilgenboecker K, Hammerstein P, Schlattmann P, Telschow A, Werren JH (2008). How many species are infected with *Wolbachia*? - a statistical analysis of current data. FEMS Microbiol Lett.

[8] Russell JA, Latorre A, Sabater-Munoz B, Moya A, Moran NA (2003). Side-stepping secondary symbionts: widespread horizontal transfer across and beyond the Aphidoidea. Mol Ecol.

[9] Vavre F, Fleury F, Lepetit D, Fouillet P, Bouletreau M (1999). Phylogenetic evidence for horizontal transmission of *Wolbachia* in host-parasitoid associations. Mol Biol Evol.

[10] Noda H, Miyoshi T, Zhang Q, Watanabe K, Deng K, Hoshizaki S (2001). *Wolbachia* infection shared among planthoppers (Homoptera : Delphacidae) and their endoparasite (Strepsiptera : Elenchidae): a probable case of interspecies transmission. Mol Ecol.

[11] Shoemaker DD, Machado CA, Molbo D, Werren JH, Windsor DM, Herre EA (2002). The distribution of *Wolbachia* in fig wasps: correlations with host phylogeny, ecology and population structure. Proc. R. Soc. B Biol. Sci..

[12] Baldo L, Ayoub NA, Hayashi CY, Russell JA, Stahlhut JK, Werren JH (2008). Insight into the routes of *Wolbachia* invasion: high levels of horizontal transfer in the spider genus Agelenopsis revealed by *Wolbachia* strain and mitochondrial DNA diversity. Mol Ecol.

[13] Ahmed MZ, De Barro PJ, Ren S-X, Greeff JM, Qiu B-L (2013). Evidence for Horizontal Transmission of Secondary Endosymbionts in the Bemisia tabaci Cryptic Species Complex. PloS One.

[14] Boyle L, Oneill SL, Robertson HM, Karr TL (1993). Interspecific and intraspecific horizontal transfer of *Wolbachia* in Drosophila. Science.

[15] Heath BD, Butcher RDJ, Whitfield WGF, Hubbard SF (1999). Horizontal transfer of *Wolbachia* between phylogenetically distant insect species by a naturally occurring mechanism. Curr Biol.

[16] Kang L, Ma X, Cai L, Liao S, Sun L, Zhu H, Chen X, Shen D, Zhao S, Li C. Superinfection of Laodelphax striatellus with *Wolbachia* from *Drosophila simulans*. Heredity. 2003;90(1):71–6. 10.1038/sj.hdy.6800180 12522428

[17] Zabalou S, Riegler M, Theodorakopoulou M, Stauffer C, Savakis C, Bourtzis K (2004). *Wolbachia*-induced cytoplasmic incompatibility as a means for insect pest population control. Proc Natl Acad Sci U S A.

[18] Riegler M, Charlat S, Stauffer C, Mercot H (2004). *Wolbachia* transfer from *Rhagoletis cerasi* to *Drosophila simulans*: Investigating the outcomes of host-symbiont coevolution. Appl Environ Microbiol.

[19] Van Nieukerken EJ, Kaila L, Kitching IJ, Kristensen NP, Lees DC, Minet J, Mitter C, Mutanen M, Regier JC, Simonsen TJ et al. Order Lepidoptera Linnaeus, 1758. Zootaxa. 2011;3148:212–21.

[20] Banziger H (1992). Remarkable new cases of moths drinking human tears in Thailand (Lepidoptera: Thyatritridae, Sphingidae, Notodontidae). Nat Hist Bull Siam Soc.

[21] Pierce NE (1995). Predatory and parasitic Lepidoptera: Carnivores living on plants. J Lepid Soc.

[22] Zaspel JM, Weller SJ, Branham MA (2011). A comparative survey of proboscis morphology and associated structures in fruit-piercing, tear-feeding, and blood-feeding moths in Calpinae (Lepidoptera: Erebidae). Zoomorphology.

[23] Plotkin D, Goddard J (2013). Blood, sweat, and tears: a review of the hematophagous, sudophagous, and lachryphagous Lepidoptera. J Vector Ecol.

[24] Roe AD, Weller SJ, Baixeras J, Brown J, Cummings MP, Davis DR, Kawahara AY, Parr CS, Regier JC, Rubinoff D (2010). Evolutionary framework for Lepidoptera model systems.

[25] Feener DH, Brown BV (1997). Diptera as parasitoids. Annu Rev Entomol.

[26] Whitfield JB (1998). Phylogeny and evolution of host-parasitoid interactions in hymenoptera. Annu Rev Entomol.

[27] Pennacchio F, Strand MR (2006). Evolution of developmental strategies in parasitic hymenoptera. Annu Rev Entomol.

[28] Werren JH, Baldo L, Clark ME (2008). *Wolbachia*: master manipulators of invertebrate biology. Nat Rev Microbiol.

[29] Ahmed MZ, Araujo-Jnr EV, Welch JJ, Kawahara AY (2015). *Wolbachia* in butterflies and moths: geographic structure in infection frequency. Front Zool.

[30] Ahmed MZ, Greyvenstein OFC, Erasmus C, Welch JJ, Greeff JM (2013). Consistently high incidence of *Wolbachia* in global fig wasp communities. Ecol Entomol.

[31] Sasaki T, Ishikawa H (1999). *Wolbachia* infections and cytoplasmic incompatibility in the almond moth and the mediterranean flour moth. Zool Sci.

[32] Kageyama D, Nishimura G, Hoshizaki S, Ishikawa Y (2002). Feminizing *Wolbachia* in an insect, *Ostrinia furnacalis* (Lepidoptera : Crambidae). Heredity.

[33] Dyson EA, Hurst GDD (2004). Persistence of an extreme sex-ratio bias in a natural population. Proc Natl Acad Sci U S A.

[34] Graham RI, Grzywacz D, Mushobozi WL, Wilson K (2012). *Wolbachia* in a major African crop pest increases susceptibility to viral disease rather than protects. Ecol Lett.

[35] Hughes GL, Rasgon JL (2014). Transinfection: a method to investigate *Wolbachia*-host interactions and control arthropod-borne disease. Insect Mol Biol.

[36] Hotopp JCD, Clark ME, Oliveira DCSG, Foster JM, Fischer P, Munoz Torres MC, Giebel JD, Kumar N, Ishmael N, Wang S (2007). Widespread lateral gene transfer from intracellular bacteria to multicellular eukaryotes. Science.

[37] Gyles C, Boerlin P (2014). Horizontally transferred genetic elements and their role in pathogenesis of bacterial disease. Vet Pathol.

[38] Hotopp JCD (2011). Horizontal gene transfer between bacteria and animals. Trends Genet.

[39] Robinson KM, Sieber KB, Hotopp JCD (2013). A review of bacteria-animal lateral gene transfer may inform our understanding of diseases like cancer. PloS Genet.

[40] Wheeler D, Redding AJ, Werren JH (2013). Characterization of an ancient lepidopteran lateral gene transfer. PloS One.

[41] Baldo L, Hotopp JCD, Jolley KA, Bordenstein SR, Biber SA, Choudhury RR, Hayashi C, Maiden MCJ, Tettelin H, Werren JH (2006). Multilocus sequence typing system for the endosymbiont *Wolbachia pipientis*. Appl Environ Microbiol.

[42] Jolley KA, Maiden MCJ (2010). BIGSdb: Scalable analysis of bacterial genome variation at the population level. BMC Bioinf.

[43] Katoh K, Kuma K, Toh H, Miyata T (2005). MAFFT version 5: improvement in accuracy of multiple sequence alignment. Nucleic Acids Res.

[44] Kearse M, Moir R, Wilson A, Stones-Havas S, Cheung M, Sturrock S, Buxton S, Cooper A, Markowitz S, Duran C (2012). Geneious Basic: An integrated and extendable desktop software platform for the organization and analysis of sequence data. Bioinformatics.

[45] Lanfear R, Calcott B, Ho SYW, Guindon S (2012). PartitionFinder: Combined selection of partitioning schemes and substitution models for phylogenetic analyses. Mol Biol Evol.

[46] Stamatakis A (2014). RAxML version 8: a tool for phylogenetic analysis and post-analysis of large phylogenies. Bioinformatics.

[47] Kawahara AY, Breinholt JW, Ponce FV, Haxaire J, Xiao L, Lamarre GPA, Rubinoff D, Kitching IJ (2013). Evolution of Manduca sexta hornworms and relatives: Biogeographical analysis reveals an ancestral diversification in Central America. Mol Phylogenet Evol.

[48] Guindon S, Dufayard J-F, Lefort V, Anisimova M, Hordijk W, Gascuel O (2010). New Algorithms and Methods to Estimate Maximum-Likelihood Phylogenies: Assessing the Performance of PhyML 3.0. Syst Biol.

[49] Didelot X, Falush D (2007). Inference of bacterial microevolution using multilocus sequence data. Genetics.

[50] Gelman A, DB R (1992). Inference from iterative simulation using multiple sequences. Stat Sci.

[51] Hart MW, Sunday J (2007). Things fall apart: biological species form unconnected parsimony networks. Biol Lett.

[52] Chen H, Strand M, Norenburg JL, Sun S, Kajihara H, Chernyshev AV, Maslakova SA, Sundberg P (2010). Statistical parsimony networks and species assemblages in Cephalotrichid Nemerteans (Nemertea). PloS One.

[53] De Barro P, Ahmed MZ (2011). Genetic networking of the *Bemisia tabaci* cryptic species complex reveals pattern of biological invasions. PloS One.

[54] Pfarr K, Foster J, Slatko B, Hoerauf A, Eisen JA (2007). On the taxonomic status of the intracellular bacterium *Wolbachia pipientis*: should this species name include the intracellular bacteria of filarial nematodes?. Int J Syst Evol Microbiol.

[55] Lo N, Paraskevopoulos C, Bourtzis K, O’Neill SL, Werren JH, Bordenstein SR, Bandi C (2007). Taxonomic status of the intracellular bacterium *Wolbachia pipientis*. Int J Syst Evol Microbiol.

[56] Posada D, Crandall KA (2001). Intraspecific gene genealogies: trees grafting into networks. Trends Ecol Evol.

[57] Clement M, Posada D, Crandall KA (2000). TCS: a computer program to estimate gene genealogies. Mol Ecol.

[58] Templeton AR, Crandall KA, Sing CF (1992). A Cladistic Analysis of phenotypic associations with haplotypes inferred from restriction endonuclease mapping and DNA sequence data. III. cladogram estimation. Genetics.

[59] Regier JC, Mitter C, Zwick A, Bazinet AL, Cummings MP, Kawahara AY, Sohn J-C, Zwickl DJ, Cho S, Davis DR (2013). A large-scale, higher-level, molecular phylogenetic study of the insect order lepidoptera (Moths and Butterflies). PLoS ONE.

[60] Smouse PE, Long JC, Sokal RR (1986). Multiple regression and correlation extensions of the mantel test of matrix correspondence. Syst Zool.

[61] Maddison WP MD. Mesquite: A modular system for evolutionary analysis. Version 3.04. Available from http://mesquiteproject.org. 2015.

[62] Conow C, Fielder D, Ovadia Y, Libeskind-Hadas R (2010). Jane: a new tool for the cophylogeny reconstruction problem. Algorithms Mol Biol.

[63] Bouckaert R, Heled J, Kuehnert D, Vaughan T, Wu C-H, Xie D, Suchard MA, Rambaut A, Drummond AJ (2014). BEAST 2: A Software Platform for Bayesian Evolutionary Analysis. PloS Comput Biol.

[64] Drummond AJ, Suchard MA, Xie D, Rambaut A (2012). Bayesian Phylogenetics with BEAUti and the BEAST 1.7.. Mol Biol Evol.

[65] Richardson MF, Weinert LA, Welch JJ, Linheiro RS, Magwire MM, Jiggins FM, Bergman CM (2012). Population genomics of the *Wolbachia* endosymbiont in *Drosophila melanogaster*. PLoS Genet.

[66] Gerth M, Roethe J, Bleidorn C (2013). Tracing horizontal *Wolbachia* movements among bees (Anthophila): a combined approach using multilocus sequence typing data and host phylogeny. Mol Ecol.

[67] Felsenstein J (1981). Evolutionary trees from DNA sequences: a maximum likelihood approach. J Mol Evol.

[68] Swofford DL, Sullivan J (2009). Phylogeny inference based on parsimony and other methods using PAUP*.

[69] Posada D (2001). Unveiling the molecular clock in the presence of recombination. Mol Biol Evol.

[70] Pond SLK, Frost SDW, Muse SV (2005). HyPhy: hypothesis testing using phylogenies. Bioinformatics.

[71] Boni MF, Posada D, Feldman MW (2007). An exact nonparametric method for inferring mosaic structure in sequence triplets. Genetics.

[72] Gerth M, Bleidorn C (2013). A multilocus sequence typing (MLST) approach to diminish the problems that are associated with DNA barcoding: A reply to Stahlhut etal. 2012. Syst Biodivers.

[73] Rambaut A, Suchard M, Xie D, Drummond A. Tracer v1.6, Available from http://beast.bio.ed.ac.uk/Tracer. 2014. Accessed 23 Apr. 2016.

[74] Wahlberg N, Wheat CW, Pena C (2013). Timing and patterns in the taxonomic diversification of lepidoptera (Butterflies and Moths). PloS One.

[75] Misof B, Liu S, Meusemann K, Peters RS, Donath A, Mayer C, Frandsen PB, Ware J, Flouri T, Beutel RG (2014). Phylogenomics resolves the timing and pattern of insect evolution. Science.

[76] Delcher AL, Phillippy A, Carlton J, Salzberg SL (2002). Fast algorithms for large-scale genome alignment and comparison. Nucleic Acids Res.

[77] Foster J, Ganatra M, Kamal I, Ware J, Makarova K, Ivanova N, Bhattacharyya A, Kapatral V, Kumar S, Posfai J (2005). The *Wolbachia* genome of *Brugia malayi*: Endosymbiont evolution within a human pathogenic nematode. PloS Biol.

[78] Duplouy A, Iturbe-Ormaetxe I, Beatson SA, Szubert JM, Brownlie JC, McMeniman CJ, McGraw EA, Hurst GDD, Charlat S, O'Neill SL et al. Draft genome sequence of the male-killing *Wolbachia* strain wBol1 reveals recent horizontal gene transfers from diverse sources. BMC Genomics. 2013;14:20. 10.1186/1471-2164-14-20 PMC3639933 23324387

[79] Wu M, Sun LV, Vamathevan J, Riegler M, Deboy R, Brownlie JC, McGraw EA, Martin W, Esser C, Ahmadinejad N (2004). Phylogenomics of the reproductive parasite *Wolbachia pipientis* wMel: A streamlined genome overrun by mobile genetic elements. PloS Biol.

[80] Klasson L, Walker T, Sebaihia M, Sanders MJ, Quail MA, Lord A, Sanders S, Earl J, O'Neill SL, Thomson N et al. Genome evolution of *Wolbachia* strain wPip from the *Culex pipiens* group. Mol Biol Evol. 2008;25(9):1877–87. 10.1093/molbev/msn133 PMC2515876 18550617

[81] Klasson L, Westberg J, Sapountzis P, Naesiund K, Lutnaes Y, Darby AC, Veneti Z, Chen L, Braig HR, Garrett R (2009). The mosaic genome structure of the *Wolbachia* wRi strain infecting *Drosophila simulans*. Proc Natl Acad Sci U S A.

[82] Mavingui P, Moro CV, Van T-V, Wisniewski-Dye F, Raquin V, Minard G, Tran F-H, Voronin D, Rouy Z, Bustos P et al. Whole-genome sequence of *Wolbachia* strain wAlbB, an endosymbiont of tiger mosquito vector *Aedes albopictus*. J Bacteriol. 2012;194(7):1840–0. 10.1128/JB.00036-12 PMC3302458 22408242

[83] Kent BN, Salichos L, Gibbons JG, Rokas A, Newton ILG, Clark ME, Bordenstein SR (2011). Complete bacteriophage transfer in a bacterial endosymbiont (*Wolbachia*) determined by targeted genome capture. Genome Biol Evol.

[84] Ellegaard KM, Klasson L, Naslund K, Bourtzis K, Andersson SGE (2013). Comparative genomics of *Wolbachia* and the bacterial species concept. PloS Genet.

[85] Mita K, Kasahara M, Sasaki S, Nagayasu Y, Yamada T, Kanamori H, Namiki N, Kitagawa M, Yamashita H, Yasukochi Y (2004). The genome sequence of silkworm, Bombyx mori. DNA Res.

[86] Zhan S, Merlin C, Boore JL, Reppert SM (2011). The monarch butterfly genome yields insights into long-distance migration. Cell.

[87] Dasmahapatra KK, Walters JR, Briscoe AD, Davey JW, Whibley A, Nadeau NJ, Zimin AV, Hughes DST, Ferguson LC, Martin SH (2012). Butterfly genome reveals promiscuous exchange of mimicry adaptations among species. Nature (London).

[88] Ahola V, Lehtonen R, Somervuo P, Salmela L, Koskinen P, Rastas P, Välimäki N, Paulin L, Kvist J, Wahlberg N et al.: The Glanville fritillary genome retains an ancient karyotype and reveals selective chromosomal fusions in Lepidoptera. Nature Communications 2014:in press. 10.1038/ncomms5737 PMC4164777 25189940

[89] Cong Q, Borek D, Otwinowski Z, Grishin NV (2015). Tiger Swallowtail Genome Reveals Mechanisms for Speciation and Caterpillar Chemical Defense. Cell Rep.

[90] Nishikawa H, Iijima T, Kajitani R, Yamaguchi J, Ando T, Suzuki Y, Sugano S, Fujiyama A, Kosugi S, Hirakawa H (2015). A genetic mechanism for female-limited Batesian mimicry in Papilio butterfly. Nat Genet.

[91] Tang W, Yu L, He W, Yang G, Ke F, Baxter SW, You S, Douglas CJ, You M (2014). DBM-DB: the diamondback moth genome database. Database.

[92] Vos M, Didelot X (2009). A comparison of homologous recombination rates in bacteria and archaea. ISME J.

[93] Kawahara AY, Breinholt JW (2014). Phylogenomics provides strong evidence for relationships of butterflies and moths. Proc. R. Soc. B Biol. Sci..

[94] Fujii Y, Kageyama D, Hoshizaki S, Ishikawa H, Sasaki T (2001). Transfection of *Wolbachia* in Lepidoptera: the feminizer of the adzuki bean borer Ostrinia scapulalis causes male killing in the Mediterranean flour moth *Ephestia kuehniella*. Proc R Soc B Biol Sci.

[95] Kageyama D, Traut W (2004). Opposite sex-specific effects of *Wolbachia* and interference with the sex determination of its host *Ostrinia scapulalis*. Proc R Soc B Biol Sci.

[96] Sasaki T, Ishikawa H (2000). Transinfection of *Wolbachia* in the Mediterranean flour moth, *Ephestia kuehniella*, by embryonic microinjection. Heredity.

[97] Sakamoto H, Ishikawa Y, Sasaki T, Kikuyama S, Tatsuki S, Hoshizaki S (2005). Transinfection reveals the crucial importance of *Wolbachia* genotypes in determining the type of reproductive alteration in the host. Genet Res.

[98] Sasaki T, Kubo T, Ishikawa H (2002). Interspecific transfer of *Wolbachia* between two lepidopteran insects expressing cytoplasmic incompatibility: A *Wolbachia* variant naturally infecting *Cadra cautella* causes male killing in *Ephestia kuehniella*. Genetics.

[99] Kageyama D, Narita S, Noda H (2008). Transfection of Feminizing *Wolbachia* Endosymbionts of the Butterfly, *Eurema hecabe*, into the Cell Culture and Various Immature Stages of the Silkmoth, Bombyx mori. Microb Ecol.

[100] Hornett EA, Charlat S, Duplouy AMR, Davies N, Roderick GK, Wedell N, Hurst GDD (2006). Evolution of male-killer suppression in a natural population. PLoS Biol.

[101] Hornett EA, Duplouy AMR, Davies N, Roderick GK, Wedell N, Hurst GDD, Charlat S (2008). You can’t keep a good parasite down: Evolution of a male-killer suppressor uncovers cytoplasmic incompatibility. Evolution.

[102] Huigens ME, Luck RF, Klaassen RHG, Maas M, Timmermans M, Stouthamer R (2000). Infectious parthenogenesis. Nature.

[103] Huigens ME, de Almeida RP, Boons PAH, Luck RF, Stouthamer R (2004). Natural interspecific and intraspecific horizontal transfer of parthenogenesis-inducing *Wolbachia* in Trichogramma wasps. Proc R Soc B Biol Sci.

[104] Rigaud T, Juchault P (1995). Success and failure of horizontal transfers of feminizing *Wolbachia* endosymbionts in woodlice. J Evol Biol.

[105] Sintupachee S, Milne JR, Poonchaisri S, Baimai V, Kittayapong P (2006). Closely related *Wolbachia* strains within the pumpkin arthropod community and the potential for horizontal transmission via the plant. Microb Ecol.

[106] Gehrer L, Vorburger C (2012). Parasitoids as vectors of facultative bacterial endosymbionts in aphids. Biol Lett.

[107] Jaenike J, Polak M, Fiskin A, Helou M, Minhas M (2007). Interspecific transmission of endosymbiotic Spiroplasma by mites. Biol Lett.

[108] Apiwathnasorn C (2012). Literature review of parasitoids of filth flies in Thailand: a list of species with brief notes on bionomics of common species. Southeast Asian J Trop Med Public Health.

[109] Poll JTK, Alphen JJM, Driessen GJJ (1998). Biological control of the asparagus beetle (Crioceris asparagi) using *Tetrastichus asparagi*. Proc Sect Exp Appl Entomol Neth Entomol Soc.

[110] Jha RK, Tuan S, Chi H, Tang L (2014). Life table and consumption capacity of corn earworm, Helicoverpa armigera, fed asparagus, Asparagus officinalis. J Insect Sci.

[111] Madden AA, Davis MM, Starks PT (2010). First detailed report of brood parasitoidism in the invasive population of the paper wasp Polistes dominulus (Hymenoptera, Vespidae) in North America. Insect Soc.

[112] Waldbauer G (2003). What good are bugs? Insects in the web of life.

[113] Le Clec’h W, Chevalier FD, Genty L, Bertaux J, Bouchon D, Sicard M (2013). Cannibalism and predation as paths for horizontal passage of *Wolbachia* between terrestrial isopods. PloS One.

[114] Mason PG, Miall JH, Bouchard P, Brauner A, Gillespie DR, Gibson GAP (2014). The parasitoid communities associated with Ceutorhynchus species (Coleoptera: Curculionidae) in Ontario and Quebec, Canada. Can Entomol.

[115] Juran I, Culjak TG, Grubisic D (2011). Rape stem weevil (Ceutorhynchus napi Gyll. 1837) and cabbage stem weevil (Ceutorhynchus pallidactylus Marsh. 1802) (Coleoptera: Curculionidae) - Important oilseed rape pests. Agric Conspec Sci.

[116] Oliver KM, Moran NA, Hunter MS (2005). Variation in resistance to parasitism in aphids is due to symbionts not host genotype. Proc Natl Acad Sci U S A.

[117] Scarborough CL, Ferrari J, Godfray HCJ. Aphid protected from pathogen by endosymbiont. Science. 2005;310(5755):1781–1. 10.1126/science.1120180 16357252

[118] Xue X, Li S-J, Ahmed MZ, De Barro PJ, Ren S-X, Qiu B-L (2012). Inactivation of *Wolbachia* Reveals Its Biological Roles in Whitefly Host. Plos One.

[119] Teixeira L, Ferreira A, Ashburner M (2008). The bacterial symbiont *Wolbachia* induces resistance to RNA viral infections in Drosophila melanogaster. Plos Biology.

[120] Derks MFL, Smit S, Salis L, Schijlen E, Bossers A, Mateman C, Pijl AS, de Ridder D, Groenen MAM, Visser ME (2015). The Genome of Winter Moth (Operophtera brumata) Provides a Genomic Perspective on Sexual Dimorphism and Phenology. Genome Biology and Evolution.

