# Supplementary figures and images for: Evidence for common horizontal transmission of Wolbachia among butterflies and moths
Source: BMC Evol Biol. 2016 May 27;16:118. doi: 10.1186/s12862-016-0660-x (PMC4882834; doi:10.1186/s12862-016-0660-x)

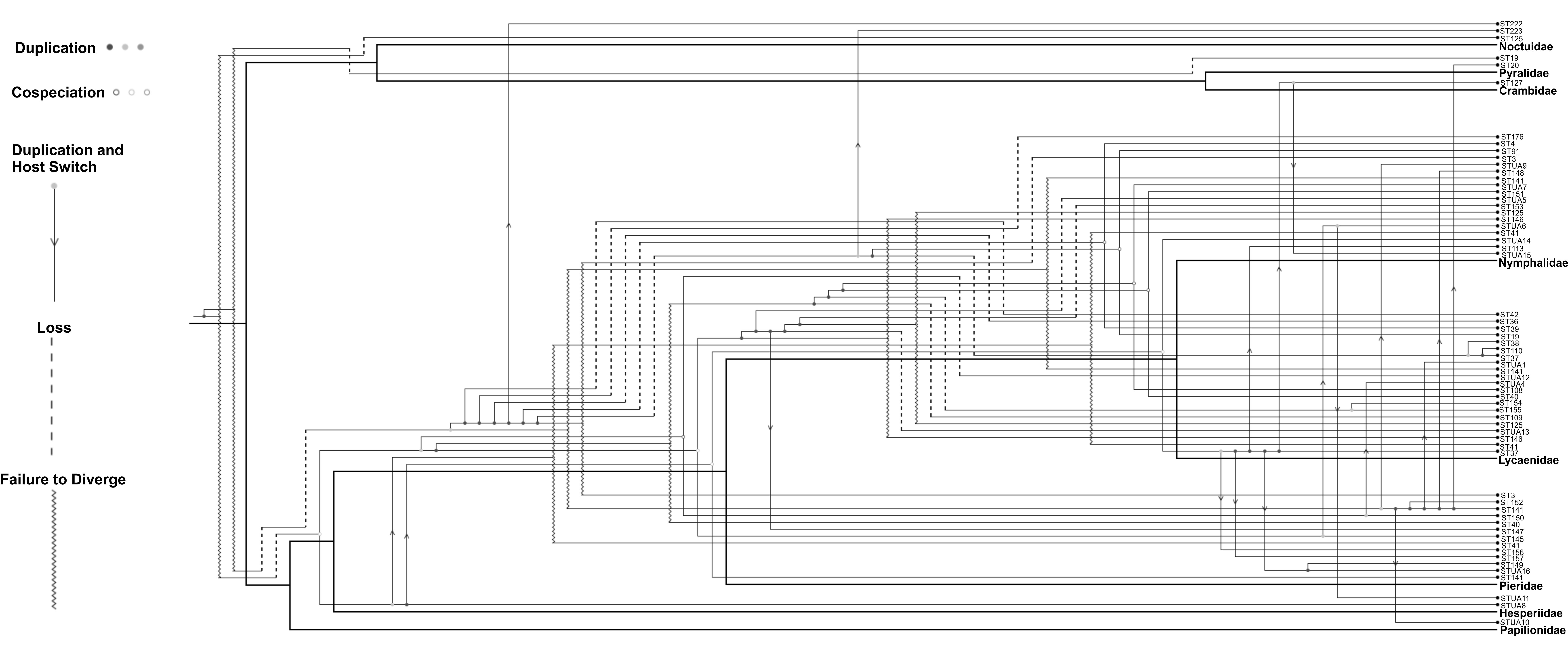

Supplement: Additional file 4: Figure S1. — Wolbachia ClonalFrame genealogy (in grey, not drawn to scale) based on five MLST genes mapped onto the host family phylogeny with JANE [62]. Host family phylogeny is redrawn from Regier et al. [59]. Small solid circles show duplicated strains; small open circles show co-speciation of strains; arrows show host switches; dotted lines show loss of strains and zigzag lines show failure in strain divergence. (JPG 2300 kb) [file 12862_2016_660_MOESM4_ESM.jpg]
