# Supplementary material for: Evidence for common horizontal transmission of Wolbachia among butterflies and moths
Source: BMC Evol Biol. 2016 May 27;16:118. doi: 10.1186/s12862-016-0660-x (PMC4882834; doi:10.1186/s12862-016-0660-x)
Supplement: Additional file 5: — Tree file. Chronogram from a median consenus tree based on 10,000 trees from the posterior distbution of the rate [65] and clade calibrated [66] beast runs. (PDF 261 kb) [file 12862_2016_660_MOESM5_ESM.pdf]

#NEXUS

Begin taxa;

Dimensions ntax=93;

Taxlabels

13_Ekue_A_Ephestia_Pyralidae_Lepidoptera_

22_Aenc_B_Ugardan_Acraea_Nymphalidae_Lepidoptera_

23_Aepo_B_Acraea_Nymphalidae_Lepidoptera_

31_Ekue_B_Ephestia_Pyralidae_Lepidoptera_

32_Osca_B_Ostrinia_Crambidae_Lepidoptera_

38_Hbol_A_wBol2_Hypolimnas_Nymphalidae_Lepidoptera_French_Polynesia

39_Lida_B_Lycaeides_Lycaenidae_Lepidoptera_USA

40_Hbol_B_wBol1_Hypolimnas_Nymphalidae_Lepidoptera_French_Polynesia

69_Pdom_B_JKS1_Polistes_Vespidae_Hymenoptera_USA

74_A_ElmMoth86___Lepidoptera_USA

97_Aemo_B_Anthene_Lycaenidae_Lepidoptera_Malaysia

98_Jale_A_Jamides_Lycaenidae_Lepidoptera_Malaysia

99_Hony_B_Horaga_Lycaenidae_Lepidoptera_Malaysia

100_Sviv_B_Surendra_Lycaenidae_Lepidoptera_Malaysia

101_Nang_B_Nacaduba_Lycaenidae_Lepidoptera_Malaysia

102_Sepi_B_Spalgis_Lycaenidae_Lepidoptera_Malaysia

111_Talb_A_Technomyrmex_Formicidae_Hymenoptera_Philippines

115_Lept_A_Leptomyrmex_Formicidae_Hymenoptera_Australia

123_Opeu_A_Ornipholidotos_Lycaenidae_Lepidoptera_South_Africa

124_Ppla_A_20-05_Pheidole_Formicidae_Hymenoptera_Thailand

128_Amir_B_Azanus_Lycaenidae_Lepidoptera_Ghana

130_Bfel_B_Brangas_Lycaenidae_Lepidoptera_Ecuador

131_Carg_B_Celastrina_Lycaenidae_Lepidoptera_USA

132_Tthe_B_Thersamonia_Lycaenidae_Lepidoptera_Russia

133_Iroc_A_Iraota_Lycaenidae_Lepidoptera_Malaysia

136_Lmyr_B_Libythea_Nymphalidae_Lepidoptera_Malaysia

146_Lepg_A_06-03_Leptogenys_Formicidae_Hymenoptera_Thailand

147_Ppln_A_18-04_Pheidole_Formicidae_Hymenoptera_Thailand

156_B_NY_Calyp150744b___Diptera_USA

193_Cela_B_Colias_Pieridae_Lepidoptera_Japan

194_Cela_B_Colias_Pieridae_Lepidoptera_Japan

195_Eman_B_CI_Eurema_Pieridae_Lepidoptera_Japan

196_Eman_B_CI_Eurema_Pieridae_Lepidoptera_Taiwan

197_Eman_B_CI_Eurema_Pieridae_Lepidoptera_Japan

198_Eman_B_CI_Eurema_Pieridae_Lepidoptera_Japan

199_Eman_B_CI_Eurema_Pieridae_Lepidoptera_Japan

200_Eman_B_Fem_Eurema_Pieridae_Lepidoptera_Japan

201_Eman_B_Fem_Eurema_Pieridae_Lepidoptera_Japan

208_Amer_B_wMer_Ariadne_Nymphalidae_Lepidopera_India

209_Cama_B_wAma_Colotis_Pieridae_Lepidopera_India

210_Cama_B_wAma_Colotis_Pieridae_Lepidopera_India

212_Cner_B_wNer_Cepora_Pieridae_Lepidopera_India

216_Cros_B_wRos_Castalius_Lycaenidae_Lepidopera_India

217_Dchr_B_wChr_Danaus_Nymphalidae_Lepidopera_India

218_Deuc_B_wEuc_Delias_Pieridae_Lepidopera_India

219_Ehec_B_wHec_Eurema_Pieridae_Lepidopera_India

220_Ehec_B_wHec_Eurema_Pieridae_Lepidopera_India

221_Ehec_B_wHec_Eurema_Pieridae_Lepidopera_India

222_Ehec_B_wHec_Eurema_Pieridae_Lepidopera_India

223_Ehec_B_wHec_Eurema_Pieridae_Lepidopera_India

224_Elae_B_wLae_Eurema_Pieridae_Lepidoptera_India

225_Hbol_B_wBol_Hypolimnas_Nymphalidae_Lepidoptera_India

228_Jiph_B_wIph_Junonia_Nymphalidae_Lepidoptera_India

229_Jlem_B_wLem_Junonia_Nymphalidae_Lepidoptera_India

230_lep_B_F21_Ixias_Pieridae_Lepidoptera_India

231_lep_B_F45_Jalmenus_Lycaenidae_Lepidoptera_India

232_lep_B_F46_Jalmenus_Lycaenidae_Lepidoptera_India

233_lep_B_F49_Udaspes_Hesperidae_Lepidoptera_India

234_lep_B_F65_Zizeeria_Lycaenidae_Lepidoptera_India

235_Lnin_B_wNin_Leptosia_Pieridae_Lepidoptera_India

237_Nhyl_B_wHyl_Neptis_Nymphalidae_Lepidoptera_India

238_Pagl_B_wAgl_Parantica_Nymphalidae_Lepidoptera_India

240_Pmah_B_wMah_Pseudozizeeria_Lycaenidae_Lepidoptera_India

241_Pmah_B_wMah_Pseudozizeeria_Lycaenidae_Lepidoptera_India

242_Pval_B_wVal_Pareronia_Pieridae_Lepidoptera_India

243_Pval_B_wVal_Pareronia_Pieridae_Lepidoptera_India

245_Tnar_B_wNar_Tarucus_Lycaenidae_Lepidoptera_India

246_Tnys_B_wNys_Telicada_Lycaenidae_Lepidoptera_India

247_Tnys_B_wNys_Telicada_Lycaenidae_Lepidoptera_India

270_wBol_B_wBol1_Hypolimnas_Nymphalidae_Lepidoptera_Japan

271_wBol_B_wBol3_Hypolimnas_Nymphalidae_Lepidoptera_Japan

293_Tcoer_B_Tetrastichus_Eulophidae_Hymenoptera_Netherlands_France_USA

297_Ehec_B_CI_Eurema_Pieridae_Lepidoptera_Japan

298_Ehec_B_Fem_Eurema_Pieridae_Lepidoptera_Japan

321_Cneg_A_Ceutorhynchus_Curculionidae_Coleoptera_Canada

322_Sexe_B_wExe1_Spodoptera_Noctuidae_Lepidoptera_Tanzania

323_Sexe_B_wExe2_Spodoptera_Noctuidae_Lepidoptera_Tanzania

324_Sexe_A_wExe3_Spodoptera_Noctuidae_Lepidoptera_Tanzania

325_wKueUSDA_Ephestia_Pyralidae_Lepidoptera_USA

NLeu

NFa

NoPa

211_Ccal_B_wCal_Caleta_Lycaenidae_Lepidopera_India

215_Cros_B_wRos_Castalius_Lycaenidae_Lepidopera_India

226_Hbol_B_wBol_Hypolimnas_Nymphalidae_Lepidoptera_India

248_Tnys_B_wNys_Telicada_Lycaenidae_Lepidoptera_India

213_Cpom_B_wPom_Catopsilia_Pieridae_Lepidopera_India

227_Hbol_B_wBol_Hypolimnas_Nymphalidae_Lepidoptera_India

236_Melanitis_leda_Melanitis_Nymphalidae_Lepidoptera_India

214_Cpom_B_wPom_Catopsilia_Pieridae_Lepidopera_India

239_Pdem_B_wDem_Papilio_Papilionidae_Lepidoptera_India

249_Yast_B_wAst_Ypthima_Nymphalidae_Lepidoptera_India

244_Tcer_B_wCer_Teractrocera_Hesperidae_Lepidoptera_India

;

End;

Begin trees;

Translate

1 13_Ekue_A_Ephestia_Pyralidae_Lepidoptera_,

2 22_Aenc_B_Ugardan_Acraea_Nymphalidae_Lepidoptera_,

3 23_Aepo_B_Acraea_Nymphalidae_Lepidoptera_,

4 31_Ekue_B_Ephestia_Pyralidae_Lepidoptera_,

5 32_Osca_B_Ostrinia_Crambidae_Lepidoptera_,

6 38_Hbol_A_wBol2_Hypolimnas_Nymphalidae_Lepidoptera_French_Polynesia,

7 39_Lida_B_Lycaeides_Lycaenidae_Lepidoptera_USA,

8 40_Hbol_B_wBol1_Hypolimnas_Nymphalidae_Lepidoptera_French_Polynesia,

9 69_Pdom_B_JKS1_Polistes_Vespidae_Hymenoptera_USA,

10 74_A_ElmMoth86___Lepidoptera_USA,

11 97_Aemo_B_Anthene_Lycaenidae_Lepidoptera_Malaysia,

12 98_Jale_A_Jamides_Lycaenidae_Lepidoptera_Malaysia,

13 99_Hony_B_Horaga_Lycaenidae_Lepidoptera_Malaysia,

14 100_Sviv_B_Surendra_Lycaenidae_Lepidoptera_Malaysia,

15 101_Nang_B_Nacaduba_Lycaenidae_Lepidoptera_Malaysia,

16 102_Sepi_B_Spalgis_Lycaenidae_Lepidoptera_Malaysia,

17 111_Talb_A_Technomyrmex_Formicidae_Hymenoptera_Philippines,

18 115_Lept_A_Leptomyrmex_Formicidae_Hymenoptera_Australia,

19 123_Opeu_A_Ornipholidotos_Lycaenidae_Lepidoptera_South_Africa,

20 124_Ppla_A_20-05_Pheidole_Formicidae_Hymenoptera_Thailand,

21 128_Amir_B_Azanus_Lycaenidae_Lepidoptera_Ghana,

22 130_Bfel_B_Brangas_Lycaenidae_Lepidoptera_Ecuador,

23 131_Carg_B_Celastrina_Lycaenidae_Lepidoptera_USA,

24 132_Tthe_B_Thersamonia_Lycaenidae_Lepidoptera_Russia,

25 133_Iroc_A_Iraota_Lycaenidae_Lepidoptera_Malaysia,

26 136_Lmyr_B_Libythea_Nymphalidae_Lepidoptera_Malaysia,

27 146_Lepg_A_06-03_Leptogenys_Formicidae_Hymenoptera_Thailand,

28 147_Ppln_A_18-04_Pheidole_Formicidae_Hymenoptera_Thailand,

29 156_B_NY_Calyp150744b___Diptera_USA,

30 193_Cela_B_Colias_Pieridae_Lepidoptera_Japan,

31 194_Cela_B_Colias_Pieridae_Lepidoptera_Japan,

32 195_Eman_B_CI_Eurema_Pieridae_Lepidoptera_Japan,

33 196_Eman_B_CI_Eurema_Pieridae_Lepidoptera_Taiwan,

34 197_Eman_B_CI_Eurema_Pieridae_Lepidoptera_Japan,

35 198_Eman_B_CI_Eurema_Pieridae_Lepidoptera_Japan,

36 199_Eman_B_CI_Eurema_Pieridae_Lepidoptera_Japan,

37 200_Eman_B_Fem_Eurema_Pieridae_Lepidoptera_Japan,

38 201_Eman_B_Fem_Eurema_Pieridae_Lepidoptera_Japan,

39 208_Amer_B_wMer_Ariadne_Nymphalidae_Lepidopera_India,

40 209_Cama_B_wAma_Colotis_Pieridae_Lepidopera_India,

41 210_Cama_B_wAma_Colotis_Pieridae_Lepidopera_India,

42 212_Cner_B_wNer_Cepora_Pieridae_Lepidopera_India,

43 216_Cros_B_wRos_Castalius_Lycaenidae_Lepidopera_India,

44 217_Dchr_B_wChr_Danaus_Nymphalidae_Lepidopera_India,

45 218_Deuc_B_wEuc_Delias_Pieridae_Lepidopera_India,

46 219_Ehec_B_wHec_Eurema_Pieridae_Lepidopera_India,

47 220_Ehec_B_wHec_Eurema_Pieridae_Lepidopera_India,

48 221_Ehec_B_wHec_Eurema_Pieridae_Lepidopera_India,

49 222_Ehec_B_wHec_Eurema_Pieridae_Lepidopera_India,

50 223_Ehec_B_wHec_Eurema_Pieridae_Lepidopera_India,

51 224_Elae_B_wLae_Eurema_Pieridae_Lepidoptera_India,

52 225_Hbol_B_wBol_Hypolimnas_Nymphalidae_Lepidoptera_India,

53 228_Jiph_B_wIph_Junonia_Nymphalidae_Lepidoptera_India,

54 229_Jlem_B_wLem_Junonia_Nymphalidae_Lepidoptera_India,

55 230_lep_B_F21_Ixias_Pieridae_Lepidoptera_India,

56 231_lep_B_F45_Jalmenus_Lycaenidae_Lepidoptera_India,

57 232_lep_B_F46_Jalmenus_Lycaenidae_Lepidoptera_India,

58 233_lep_B_F49_Udaspes_Hesperidae_Lepidoptera_India,

59 234_lep_B_F65_Zizeeria_Lycaenidae_Lepidoptera_India,

60 235_Lnin_B_wNin_Leptosia_Pieridae_Lepidoptera_India,

61 237_Nhyl_B_wHyl_Neptis_Nymphalidae_Lepidoptera_India,

62 238_Pagl_B_wAgl_Parantica_Nymphalidae_Lepidoptera_India,

63 240_Pmah_B_wMah_Pseudozizeeria_Lycaenidae_Lepidoptera_India,

64 241_Pmah_B_wMah_Pseudozizeeria_Lycaenidae_Lepidoptera_India,

65 242_Pval_B_wVal_Pareronia_Pieridae_Lepidoptera_India,

66 243_Pval_B_wVal_Pareronia_Pieridae_Lepidoptera_India,

67 245_Tnar_B_wNar_Tarucus_Lycaenidae_Lepidoptera_India,

68 246_Tnys_B_wNys_Telicada_Lycaenidae_Lepidoptera_India,

69 247_Tnys_B_wNys_Telicada_Lycaenidae_Lepidoptera_India,

70 270_wBol_B_wBol1_Hypolimnas_Nymphalidae_Lepidoptera_Japan,

71 271_wBol_B_wBol3_Hypolimnas_Nymphalidae_Lepidoptera_Japan,

72 293_Tcoer_B_Tetrastichus_Eulophidae_Hymenoptera_Netherlands_France_USA,

73 297_Ehec_B_CI_Eurema_Pieridae_Lepidoptera_Japan,

74 298_Ehec_B_Fem_Eurema_Pieridae_Lepidoptera_Japan,

75 321_Cneg_A_Ceutorhynchus_Curculionidae_Coleoptera_Canada,

76 322_Sexe_B_wExe1_Spodoptera_Noctuidae_Lepidoptera_Tanzania,

77 323_Sexe_B_wExe2_Spodoptera_Noctuidae_Lepidoptera_Tanzania,

78 324_Sexe_A_wExe3_Spodoptera_Noctuidae_Lepidoptera_Tanzania,

79 325_wKueUSDA_Ephestia_Pyralidae_Lepidoptera_USA,

80 NLeu,

81 NFa,

82 NoPa,

83 211_Ccal_B_wCal_Caleta_Lycaenidae_Lepidopera_India,

84 215_Cros_B_wRos_Castalius_Lycaenidae_Lepidopera_India,

85 226_Hbol_B_wBol_Hypolimnas_Nymphalidae_Lepidoptera_India,

86 248_Tnys_B_wNys_Telicada_Lycaenidae_Lepidoptera_India,

87 213_Cpom_B_wPom_Catopsilia_Pieridae_Lepidopera_India,

88 227_Hbol_B_wBol_Hypolimnas_Nymphalidae_Lepidoptera_India,

89 236_Melanitis_leda_Melanitis_Nymphalidae_Lepidoptera_India,

90 214_Cpom_B_wPom_Catopsilia_Pieridae_Lepidopera_India,

91 239_Pdem_B_wDem_Papilio_Papilionidae_Lepidoptera_India,

92 249_Yast_B_wAst_Ypthima_Nymphalidae_Lepidoptera_India,

93 244_Tcer_B_wCer_Teractrocera_Hesperidae_Lepidoptera_India

;

tree TREE1 = (((((((((1[&height=2.481984897757702E-7,height_95%_HPD={0.0,6.209593266248703E-7},height_median=2.0116567611694336E-7,height_range={0.0,4.410743713378906E-6},length=0.3720256113504136,length_95%_HPD={7.744747563265264E-7,1.2661073207855225},length_median=0.21480675041675568,length_range={7.744747563265264E-7,9.607625007629395},rate=0.002581789593246424,rate_95%_HPD={2.5295512411539186E-8,0.01024033836934},rate_median=8.879253646857156E-4,rate_range={2.5295512411539186E-8,0.2393840789665}]:0.13364414125680923,27[&height=2.481373658334704E-7,height_95%_HPD={0.0,6.221234798431396E-7},height_median=2.0116567611694336E-7,height_range={0.0,4.410743713378906E-

6},length=0.373347415294198,length_95%_HPD={4.533557330432814E-
6,1.266972541809082},length_median=0.21219883114099503,length_range={4.533557330432814E-
6,12.513703346252441},rate=0.0025242305455089866,rate_95%_HPD={1.8626985974987647E-
7,0.009831286706524},rate_median=8.872163950246034E-4,rate_range={7.171121369016126E-
8,0.3085218877393}]:0.13364414125680923)[&height=0.23238310535681891,height_95%_HPD={1.668
184995651245E-
5,0.7735995650291443},height_median=0.13364434242248535,height_range={1.668184995651245E-
5,4.032810002565384},length=0.3905650306951744,length_95%_HPD={6.959072925383225E-
5,1.3428927659988403},length_median=0.22186706960201263,length_range={6.959072925383225E-
5,6.623865127563477},posterior=0.0838235294117647,rate=0.002301060958742246,rate_95%_HPD={
4.296839883890575E-7,0.009563398540306},rate_median=8.343226803763246E-
4,rate_range={4.296839883890575E-
7,0.07299970079793}]:0.14139168057590723,18[&height=2.48001801555445E-
7,height_95%_HPD={0.0,6.221234798431396E-7},height_median=2.0116567611694336E-
7,height_range={0.0,4.351139068603516E-
6},length=0.3726095324376981,length_95%_HPD={4.533557330432814E-
6,1.2487729787826538},length_median=0.21419573575258255,length_range={4.533557330432814E-
6,8.6224946975708},rate=0.0025225400329982337,rate_95%_HPD={4.406476245968789E-
8,0.009753865922181},rate_median=8.983223086534943E-4,rate_range={4.406476245968789E-
8,0.2045866309587}]:0.27503582183271646)[&height=0.38164240700786534,height_95%_HPD={0.007
832358591258526,1.0227124392986298},height_median=0.2750360229983926,height_range={0.0050
728097558021545,3.4177278727293015},length=0.3353890457379348,length_95%_HPD={2.40871566
34777784E-
4,1.036973476409912},length_median=0.1980551779270172,length_range={2.4087156634777784E-
4,2.5510189533233643},posterior=0.018549019607843137,rate=0.0023090134296861863,rate_95%_H
PD={9.421368052578065E-7,0.008306352142438},rate_median=9.202231706906625E-
4,rate_range={9.421368052578065E-
7,0.0647309314099}]:0.34787246445193887,((17[&height=2.4812872301231884E-
7,height_95%_HPD={0.0,6.221234798431396E-7},height_median=2.0116567611694336E-
7,height_range={0.0,5.0067901611328125E-
6},length=0.3786557788397194,length_95%_HPD={8.201088348869234E-
6,1.2720715999603271},length_median=0.21881157904863358,length_range={8.201088348869234E-
6,18.295167922973633},rate=0.002513934185857845,rate_95%_HPD={6.23721107215349E-
8,0.009776330948269},rate_median=8.585236992487988E-4,rate_range={6.23721107215349E-
8,0.1947836758711}]:0.12795722857117653,19[&height=2.48127444028707E-
7,height_95%_HPD={0.0,6.183981895446777E-7},height_median=2.0116567611694336E-
7,height_range={0.0,4.351139068603516E-
6},length=0.3660756972108475,length_95%_HPD={7.744747563265264E-
7,1.2433395385742188},length_median=0.20655599981546402,length_range={7.744747563265264E-
7,7.993826866149902},rate=0.002527059964837103,rate_95%_HPD={2.5295512411539186E-
8,0.009636209486884},rate_median=8.706260870134032E-4,rate_range={2.5295512411539186E-
8,0.3295222158187}]:0.12795722857117653)[&height=0.2209591327870559,height_95%_HPD={1.0967
76541089639E-
4,0.7361962110735476},height_median=0.12795742973685265,height_range={1.096776541089639E-

4,3.9404796361923218},length=0.37358616108864423,length_95%_HPD={6.204182864166796E-
5,1.253169059753418},length_median=0.21653392910957336,length_range={6.204182864166796E-
5,5.791028022766113},posterior=0.08705882352941176,rate=0.002513476309857249,rate_95%_HPD=
{5.315562376507108E-7,0.009844768714432},rate_median=8.633307785791172E-
4,rate_range={5.315562376507108E-
7,0.1349343672806}]:0.17750508058816195,28[&height=2.481126340867109E-
7,height_95%_HPD={0.0,6.211921572685242E-7},height_median=2.0116567611694336E-
7,height_range={0.0,4.387460649013519E-
6},length=0.3685874287380953,length_95%_HPD={1.0992262104991823E-
6,1.2399537563323975},length_median=0.21315009891986847,length_range={1.0992262104991823E-
6,10.01587200164795},rate=0.002527257409817238,rate_95%_HPD={7.316730975067135E-
8,0.009552652261473},rate_median=8.775669215702322E-4,rate_range={7.316730975067135E-
8,0.4522084323742}]:0.3054623091593385)[&height=0.39897282541431467,height_95%_HPD={0.0112
59992606937885,1.068051652982831},height_median=0.3054625103250146,height_range={0.011259
992606937885,3.725874423980713},length=0.39581616348271165,length_95%_HPD={6.45121908746
6598E-
5,1.4223301410675049},length_median=0.21907378733158112,length_range={6.451219087466598E-
5,4.3259148597717285},posterior=0.02003921568627451,rate=0.0025276262443628423,rate_95%_HP
D={7.880518409613637E-7,0.009671363617662},rate_median=8.450511287906215E-
4,rate_range={7.880518409613637E-
7,0.2067988655842}]:0.31744597712531686)[&height=0.8268323654676063,height_95%_HPD={0.0883
0651640892029,2.14949868619442},height_median=0.6229084874503314,height_range={0.08830651
640892029,10.16006949916482},length=0.5130611781520867,length_95%_HPD={4.220182745484635
E-
5,1.5265942811965942},length_median=0.24274428188800812,length_range={4.220182745484635E-
5,17.400501251220703},posterior=0.00792156862745098,rate=0.0024499474697916622,rate_95%_HP
D={4.228167152616236E-6,0.01109755947317},rate_median=8.137418609655903E-
4,rate_range={4.228167152616236E-
6,0.06056199407146}]:0.18542920099571347,6[&height=2.478255124430564E-
7,height_95%_HPD={0.0,6.189802661538124E-7},height_median=2.0116567611694336E-
7,height_range={0.0,5.0067901611328125E-
6},length=0.6050827582435301,length_95%_HPD={0.0013820516178384423,1.7644885778427124},len
gth_median=0.41084733605384827,length_range={8.246956276707351E-
4,15.586605072021484},rate=0.007498735398158815,rate_95%_HPD={4.46724073342719E-
6,0.02657799898881},rate_median=0.003473083647185,rate_range={4.46724073342719E-
6,0.3187553243831}]:0.8083374872803688)[&height=0.9934299234300852,height_95%_HPD={0.20325
550436973572,2.4658044576644897},height_median=0.8083376884460449,height_range={0.1198071
1668729782,10.499233365058899},length=0.3726827382294709,length_95%_HPD={2.9599078698083
76E-
4,1.257155418395996},length_median=0.20905300974845886,length_range={2.959907869808376E-
4,7.336693286895752},posterior=0.008764705882352942,rate=0.0019779751632252435,rate_95%_HP
D={3.036929200495198E-6,0.008409147758761},rate_median=8.271717932246941E-
4,rate_range={3.036929200495198E-
6,0.04792299421878}]:0.36862173676490784,(20[&height=2.4809689479939953E-

7,height_95%_HPD={0.0,6.221234798431396E-7},height_median=2.0116567611694336E-
7,height_range={0.0,4.76837158203125E-
6},length=0.37610656098429734,length_95%_HPD={6.618725365115097E-
6,1.2476232051849365},length_median=0.21474819630384445,length_range={6.618725365115097E-
6,15.610797882080078},rate=0.002476182639341265,rate_95%_HPD={4.35321794867005E-
8,0.009604277438693},rate_median=8.895177212395425E-4,rate_range={4.35321794867005E-
8,0.3113797568273}]:0.12795530632138252,75[&height=2.481440502115933E-
7,height_95%_HPD={0.0,6.253831088542938E-7},height_median=2.0116567611694336E-
7,height_range={0.0,4.76837158203125E-
6},length=0.37079615682236494,length_95%_HPD={4.748151695821434E-
6,1.2512214183807373},length_median=0.21271832287311554,length_range={4.748151695821434E-
6,12.13351821899414},rate=0.0025830970208110273,rate_95%_HPD={2.6187788932056647E-
8,0.01011497606522},rate_median=8.920315700561316E-4,rate_range={2.6187788932056647E-
8,0.2136715526992}]:0.12795530632138252)[&height=0.23480429350349655,height_95%_HPD={8.168
816566467285E-
5,0.7861650586128235},height_median=0.12795550748705864,height_range={8.168816566467285E-
5,7.849257469177246},length=0.3884917646677116,length_95%_HPD={5.7507095334585756E-
5,1.2435414791107178},length_median=0.22295740246772766,length_range={5.7507095334585756E-
5,15.248601913452148},posterior=0.08803921568627451,rate=0.0024919323699332157,rate_95%_HP
D={1.501803678757216E-7,0.009644815433402},rate_median=8.555751091516827E-
4,rate_range={1.501803678757216E-
7,0.1803809680839}]:1.0490039177238941)[&height=1.438511865433784,height_95%_HPD={0.249286
51750087738,3.2699816823005676},height_median=1.1769594252109528,height_range={0.13598519
563674927,21.22415941953659},length=2.7941876388697646,length_95%_HPD={0.352234959602355
96,6.259051322937012},length_median=2.3384063243865967,length_range={0.15744569897651672,2
9.731178283691406},posterior=1.0,rate=0.010781505128975181,rate_95%_HPD={4.439987630968685
5E-4,0.02881837475316},rate_median=0.008000797160812,rate_range={1.7329965399035996E-
4,0.1652510362992}]:1.9838065914809704,(12[&height=2.477088232474082E-
7,height_95%_HPD={0.0,6.258487701416016E-7},height_median=2.086162567138672E-
7,height_range={0.0,5.245208740234375E-
6},length=0.8543073785926453,length_95%_HPD={0.022796986624598503,2.34661865234375},length
_median=0.620943158864975,length_range={0.010370258241891861,15.188563346862793},rate=0.00
1696485174230335,rate_95%_HPD={6.420444782087745E-
8,0.006466211391383},rate_median=6.995076255344442E-4,rate_range={6.420444782087745E-
8,0.1326462159516}]:0.6209432482719421,25[&height=2.477088232474082E-
7,height_95%_HPD={0.0,6.258487701416016E-7},height_median=2.086162567138672E-
7,height_range={0.0,5.245208740234375E-
6},length=0.8543073785926453,length_95%_HPD={0.022796986624598503,2.34661865234375},length
_median=0.620943158864975,length_range={0.010370258241891861,15.188563346862793},rate=0.00
16675055444012924,rate_95%_HPD={2.951221139123222E-
8,0.006404308580028},rate_median=6.800688728801801E-4,rate_range={2.951221139123222E-
8,0.07204603495531}]:0.6209432482719421)[&height=0.8543076263014684,height_95%_HPD={0.0227
9728651046753,2.346618890762329},height_median=0.6209434568881989,height_range={0.0103704
03528213501,15.188565492630005},length=3.436917218515394,length_95%_HPD={0.5731741189956

665,7.44284725189209},length_median=2.911140203475952,length_range={0.17867818474769592,41.69877624511719},posterior=1.0,rate=0.00568503994123862,rate_95%_HPD={1.5087321088459113E-6,0.01589889612464},rate_median=0.004006056231594,rate_range={1.5087321088459113E-6,0.1204921137975}]:2.5398225598037243)[&height=3.701923563573397,height_95%_HPD={1.149337649345398,7.69398307800293},height_median=3.160766016691923,height_range={0.8516888618469238,32.63961229566485},length=0.9203577732213682,length_95%_HPD={6.180434866109863E-5,2.5774409770965576},length_median=0.6747718751430511,length_range={6.180434866109863E-5,9.82507610321045},posterior=0.2745490196078431,rate=0.0024727120431044538,rate_95%_HPD={2.2507349486960792E-7,0.009577750898076},rate_median=9.17866722545605E-4,rate_range={2.2507349486960792E-7,0.1887078166038}]:0.9516604747623205,((10[&height=2.476563435127936E-7,height_95%_HPD={0.0,6.221234798431396E-7},height_median=2.0302832126617432E-7,height_range={0.0,4.76837158203125E-6},length=0.3134705148431028,length_95%_HPD={2.1206446035648696E-5,1.0424941778182983},length_median=0.17544088512659073,length_range={2.1206446035648696E-5,20.398164749145508},rate=0.0026770300640333857,rate_95%_HPD={4.609296499187707E-8,0.01036631310529},rate_median=9.076041711662175E-4,rate_range={4.609296499187707E-8,0.4945578777257}]:0.1754408087581396,79[&height=2.476563435127936E-7,height_95%_HPD={0.0,6.221234798431396E-7},height_median=2.0302832126617432E-7,height_range={0.0,4.76837158203125E-6},length=0.3134705148431028,length_95%_HPD={2.1206446035648696E-5,1.0424941778182983},length_median=0.17544088512659073,length_range={2.1206446035648696E-5,20.398164749145508},rate=0.0026521906057339756,rate_95%_HPD={5.579228666291059E-8,0.01051762276915},rate_median=9.117431324022368E-4,rate_range={5.579228666291059E-8,0.3230493447418}]:0.1754408087581396)[&height=0.31347076249944633,height_95%_HPD={2.139061689376831E-5,1.0424943268299103},height_median=0.17544101178646088,height_range={2.139061689376831E-5,20.39816951751709},length=2.266865069382331,length_95%_HPD={0.4291127622127533,4.93349027633667},length_median=1.9099991917610168,length_range={0.15140365064144135,41.824851989746094},posterior=1.0,rate=0.0106725123713549,rate_95%_HPD={2.0506156262290734E-4,0.02925874711586},rate_median=0.007977456869442,rate_range={2.0355857458762475E-5,0.1446435211187}]:2.000310741364956,((80[&height=2.4785449372801866E-7,height_95%_HPD={0.0,6.118789315223694E-7},height_median=2.0116567611694336E-7,height_range={0.0,5.632638931274414E-6},length=0.2315947622799087,length_95%_HPD={8.130115816129546E-7,0.7846171259880066},length_median=0.12974879890680313,length_range={8.130115816129546E-7,7.141116619110107},rate=0.003019057747068267,rate_95%_HPD={2.4459730052418645E-7,0.01168817157267},rate_median=9.904414088060766E-4,rate_range={2.4459730052418645E-7,0.3791351234248}]:0.11738472431898117,81[&height=2.478578212659676E-7,height_95%_HPD={0.0,6.118789315223694E-7},height_median=2.0116567611694336E-7,height_range={0.0,5.632638931274414E-6},length=0.23145293946858755,length_95%_HPD={8.130115816129546E-7,0.7845568060874939},length_median=0.13003724068403244,length_range={8.130115816129546E-7,6.3609395027160645},rate=0.0030789962324198976,rate_95%_HPD={5.461303983957452E-

9,0.01200435455985},rate_median=0.0010207060109834998,rate_range={5.461303983957452E-
9,0.5836258117982}]:0.11738472431898117)[&height=0.20584928151762114,height_95%_HPD={9.695
068001747131E-
7,0.6912043616175652},height_median=0.11738492548465729,height_range={9.695068001747131E-
7,4.221751362085342},length=0.4461779417337763,length_95%_HPD={1.2224697275087237E-
4,1.2997515201568604},length_median=0.311947837471962,length_range={4.455096132005565E-
5,10.792860984802246},posterior=0.908235294117647,rate=0.0024435909386503027,rate_95%_HPD=
{1.944483704171486E-8,0.009496859124297},rate_median=9.15462228298949E-
4,rate_range={1.944483704171486E-
8,0.2961448825031}]:0.380086962133646,82[&height=2.4797410252203384E-
7,height_95%_HPD={0.0,6.034970283508301E-7},height_median=2.086162567138672E-
7,height_range={0.0,5.7220458984375E-
6},length=0.6224975757339705,length_95%_HPD={0.002106989733874798,1.649294734001159 7},leng
th_median=0.4713602215051651,length_range={0.0014296448789536953,11.766324043273926},rate=
0.0020361143665717 18,rate_95%_HPD={1.2250571820941557E-
8,0.008106462361012},rate_median=7.929121802767983E-4,rate_range={1.2250571820941557E-
8,0.08405890337575}]:0.4974716790020466)[&height=0.6510194820315036,height_95%_HPD={0.0182
466059923172,1.709570288658142},height_median=0.4974718876183033,height_range={0.00513331
21955394745,11.766328573226929},length=1.9293163498502737,length_95%_HPD={0.318642139434
81445,4.301111698150635},length_median=1.5988324284553528,length_range={0.184117317199707
03,39.055389404296875},posterior=1.0,rate=0.020985166553485438,rate_95%_HPD={0.001254169056
521,0.05563839427759},rate_median=0.015636036811655,rate_range={3.9283203088776833E-
4,0.2508688901194}]:1.6782798655331135)[&height=2.580335831881778,height_95%_HPD={0.618634
9634081125,5.415437459945679},height_median=2.175751753151417,height_range={0.39023640751
838684,42.84776496887207},length=1.6173713484182954,length_95%_HPD={0.1767803430557251,3.
903653860092163},length_median=1.312515139579773,length_range={0.0685066431760788,17.9539
20364379883},posterior=1.0,rate=0.011967687128650005,rate_95%_HPD={9.097024467851147E-
7,0.03412926749139},rate_median=0.0082810297339965,rate_range={9.097024467851147E-
7,0.2756651147644}]:1.9366747383028269)[&height=4.831583817473106,height_95%_HPD={1.535300
0462055206,9.902404576539993},height_median=4.112426491454244,height_range={1.11130691319
70406,53.44737672805786},length=2.7163359796428215,length_95%_HPD={0.3401557207107544,6.4
61299896240234},length_median=2.1718218326568604,length_range={0.17191514372825623,29.439
973831176758},posterior=1.0,rate=0.051334422273249886,rate_95%_HPD={2.050504467997688E-
6,0.1348602004719},rate_median=0.03897610468184,rate_range={2.050504467997688E-
6,0.5256331460135}]:2.140341190621257,78[&height=2.5092692558647444E-
7,height_95%_HPD={0.0,7.152557373046875E-7},height_median=2.0116567611694336E-
7,height_range={0.0,4.76837158203125E-
6},length=7.4846276313230105,length_95%_HPD={2.353759527206421,15.331561088562012},length_
median=6.4120612144470215,length_range={1.6307320594787598,68.15059661865234},rate=0.00126
95754130285872,rate_95%_HPD={1.5908891506502784E-
7,0.004508460543418},rate_median=6.606874056281468E-4,rate_range={1.5908891506502784E-
7,0.02757525641486}]:6.252767480909824)[&height=7.277516051103041,height_95%_HPD={2.375291
518867016,14.840328216552734},height_median=6.2527676820755005,height_range={1.6307321432
977915,68.15059995651245},length=3.768239491592959,length_95%_HPD={0.011225888505578041,8

.534764289855957},length_median=3.0905487537384033,length_range={3.51060793036595E-4,60.882118225097656},posterior=0.9408823529411765,rate=0.05692789953522447,rate_95%_HPD={2.603421695094568E-7,0.1681783603686},rate_median=0.04270863749038,rate_range={2.603421695094568E-7,0.6083910657935}]:1.155592154711485,(((2[&height=2.472600951107904E-7,height_95%_HPD={0.0,6.258487701416016E-7},height_median=2.086162567138672E-7,height_range={0.0,5.0067901611328125E-6},length=2.627073870131426,length_95%_HPD={4.766031343024224E-4,6.633846282958984},length_median=2.025168538093567,length_range={4.766031343024224E-4,38.13777160644531},rate=0.0130834262237087,rate_95%_HPD={5.769980870614288E-7,0.03400550917863},rate_median=0.010000487122111,rate_range={5.769980870614288E-7,0.2186084606909}]:2.0473087653517723,(3[&height=2.473154557322892E-7,height_95%_HPD={0.0,6.109476089477539E-7},height_median=2.086162567138672E-7,height_range={0.0,5.4836273193359375E-6},length=0.88461133392598,length_95%_HPD={0.03317786753177643,2.3152647018432617},length_median=0.6756069958209991,length_range={0.006834103260189295,14.325352668762207},rate=0.010237864609592234,rate_95%_HPD={2.226264412141887E-5,0.03342774227683},rate_median=0.006005222786823,rate_range={2.226264412141887E-5,0.2381718940348}]:0.6755395010113716,13[&height=2.4731558356087783E-7,height_95%_HPD={0.0,6.109476089477539E-7},height_median=2.086162567138672E-7,height_range={0.0,5.4836273193359375E-6},length=0.8845264604504074,length_95%_HPD={0.03317786753177643,2.3152647018432617},length_median=0.6754208505153656,length_range={0.006834103260189295,14.325352668762207},rate=0.0016390789724092846,rate_95%_HPD={1.3575153566623855E-8,0.006183242652163},rate_median=6.863549781844834E-4,rate_range={1.3575153566623855E-8,0.1337990573725}]:0.6755395010113716)[&height=0.8846391827491183,height_95%_HPD={0.03317804075777531,2.3154779225587845},height_median=0.6755397096276283,height_range={0.006834298372268677,14.325354218482971},length=1.7367843476557012,length_95%_HPD={0.05114508047699928,4.5072021484375},length_median=1.337901532649994,length_range={0.014507967047393322,31.75271224975586},posterior=0.9995686274509804,rate=0.0101429723483462,rate_95%_HPD={8.991772221019428E-5,0.03049203278277},rate_median=0.006166908002719,rate_range={6.3491693593455476E-6,0.6077097860636}]:1.3717692643404007)[&height=2.4686650052254473,height_95%_HPD={0.41720379889011383,5.55898916721344},height_median=2.047308973968029,height_range={0.17340460792183876,19.807071696966887},length=1.7180163592430495,length_95%_HPD={0.0406881645321846,4.600032806396484},length_median=1.274156928062439,length_range={5.764577072113752E-4,29.65154457092285},posterior=0.885,rate=0.005294465275433049,rate_95%_HPD={9.626128820276336E-7,0.01731707787016},rate_median=0.00291260125148,rate_range={9.626128820276336E-7,0.3094014677275}]:1.3331962078809738,77[&height=2.467662179451865E-7,height_95%_HPD={0.0,6.556510925292969E-7},height_median=2.086162567138672E-7,height_range={0.0,5.9604644775390625E-6},length=4.007253109917921,length_95%_HPD={0.8047580718994141,9.096822738647461},length_median=3.341024875640869,length_range={0.1633726954460144,35.40099334716797},rate=0.005966210593081358,rate_95%_HPD={3.616915646148872E-

4,0.01517895789733},rate_median=0.004629526371527,rate_range={1.806445038586136E-
5,0.0753542391431}]:3.380504973232746)[&height=4.06737661100757,height_95%_HPD={0.96253052
72638798,9.075209736824036},height_median=3.380505181849003,height_range={0.5985716432332
993,35.400999307632446},length=1.808410822607463,length_95%_HPD={0.0501081757247448,4.860
810279846191},length_median=1.333584189414978,length_range={0.002245865762233734,41.76479
3395996094},posterior=0.8054313725490196,rate=0.0032247073643578744,rate_95%_HPD={4.606750
9264664957E-
7,0.01108444334629},rate_median=0.001679259953238,rate_range={4.6067509264664957E-
7,0.1734374233236}]:1.2548903338611126,(7[&height=2.4834221470677683E-
7,height_95%_HPD={0.0,6.556510925292969E-7},height_median=2.0489096641540527E-
7,height_range={0.0,7.033348083496094E-
6},length=0.9120443022655457,length_95%_HPD={1.813485869206488E-
5,3.2963316440582275},length_median=0.4851963222026825,length_range={1.813485869206488E-
5,20.518386840820312},rate=0.0019893953501093596,rate_95%_HPD={3.916974113327786E-
8,0.007620427575936},rate_median=7.133400220773926E-4,rate_range={3.916974113327786E-
8,0.2461465342392}]:0.9884092472493649,71[&height=2.478858604214182E-
7,height_95%_HPD={0.0,6.556510925292969E-7},height_median=2.086162567138672E-
7,height_range={0.0,6.9141387939453125E-
6},length=2.146167635839356,length_95%_HPD={5.303378566168249E-
4,5.925455093383789},length_median=1.5381299257278442,length_range={5.303378566168249E-
4,30.336811065673828},rate=0.0011488055432969436,rate_95%_HPD={1.4259338049493113E-
7,0.004304640123593},rate_median=4.942739253954657E-4,rate_range={1.4259338049493113E-
7,0.1163259731357}]:0.9884092435240746)[&height=1.4294104983182607,height_95%_HPD={0.02034
8548889160156,4.094044625759125},height_median=0.9884094521403313,height_range={0.0203485
48889160156,19.54615639243275},length=3.688003228867628,length_95%_HPD={4.32289583841338
75E-
4,9.67350959777832},length_median=2.9519991874694824,length_range={4.3228958384133875E-
4,42.81769943237305},posterior=0.29523529411764704,rate=0.0012355161649944357,rate_95%_HPD
={3.34932215682107E-7,0.004909425501952},rate_median=4.823208505458048E-
4,rate_range={3.34932215682107E-
7,0.1804618938974}]:3.646986063569784)[&height=5.5317289085010755,height_95%_HPD={1.537586
3276422024,12.448891282081604},height_median=4.635395515710115,height_range={0.9786159768
7006,33.4296680688858},length=2.91993617114976,length_95%_HPD={5.723611684516072E-
4,7.2337727546691895},length_median=2.4194142818450928,length_range={5.723611684516072E-
4,20.619081497192383},posterior=0.21552941176470589,rate=0.0015861514863785614,rate_95%_HP
D={2.0675730126060028E-7,0.005717906602157},rate_median=7.167881241832684E-
4,rate_range={2.0675730126060028E-
7,0.0792168185069}]:2.77296432107687)[&height=8.77353745049771,height_95%_HPD={3.165382117
0330048,18.273379802703857},height_median=7.408359836786985,height_range={3.1653821170330
048,42.9178729057312},length=1.7151768462339738,length_95%_HPD={5.94095909036696E-
4,4.135293960571289},length_median=1.3015629053115845,length_range={5.94095909036696E-
4,29.821317672729492},posterior=0.05290196078431372,rate=0.014741947612424026,rate_95%_HPD
={6.636185082029756E-
7,0.05208914639792},rate_median=0.0064498039307820006,rate_range={6.636185082029756E-

7,0.2791221992724}]:2.6708997322712094,((((4[&height=2.4686708855361026E-
7,height_95%_HPD={0.0,5.960464477539062E-7},height_median=2.0116567611694336E-
7,height_range={0.0,4.451721906661987E-
6},length=0.8313853623612599,length_95%_HPD={0.04412950947880745,2.0688130855560303},lengt
h_median=0.6590483486652374,length_range={6.217922200448811E-
4,9.909845352172852},rate=0.009648888943137481,rate_95%_HPD={4.064496242936832E-
6,0.02957880894813},rate_median=0.0060765305241185005,rate_range={4.064496242936832E-
6,0.2159648350954}]:0.6272792220115662,(30[&height=2.470064940501859E-
7,height_95%_HPD={0.0,5.960464477539062E-7},height_median=2.0116567611694336E-
7,height_range={0.0,4.563480615615845E-
6},length=0.24205972331873402,length_95%_HPD={5.502769454324152E-
6,0.789577066898346},length_median=0.14503267407417297,length_range={5.502769454324152E-
6,7.424071788787842},rate=0.002739317546440796,rate_95%_HPD={1.3913495667950246E-
7,0.01065834866842},rate_median=9.408411875794266E-4,rate_range={1.3913495667950246E-
7,0.2188811818137}]:0.1444222703576088,31[&height=2.4700314739501504E-
7,height_95%_HPD={0.0,5.960464477539062E-7},height_median=2.0116567611694336E-
7,height_range={0.0,4.563480615615845E-
6},length=0.24213719294323222,length_95%_HPD={5.502769454324152E-
6,0.7914441227912903},length_median=0.14518719911575317,length_range={5.502769454324152E-
6,7.424071788787842},rate=0.002883110123475722,rate_95%_HPD={3.334273850086967E-
8,0.01098128599832},rate_median=9.962678865575658E-4,rate_range={3.334273850086967E-
8,0.4083208050404}]:0.1444222703576088)[&height=0.24081176664125856,height_95%_HPD={5.6345
01576423645E-
6,0.7867723256349564},height_median=0.1444224715232849,height_range={5.634501576423645E-
6,7.424072265625},length=0.589268310496245,length_95%_HPD={1.0383327025920153E-
4,1.593956708908081},length_median=0.44014137983322144,length_range={1.0383327025920153E-
4,8.446978569030762},posterior=0.9855098039215686,rate=0.0021864160672594854,rate_95%_HPD=
{1.337841687211683E-7,0.008617058247319},rate_median=8.54340486404453E-
4,rate_range={1.337841687211683E-
7,0.09484453931153}]:0.48285695165395737)[&height=0.788159968506124,height_95%_HPD={0.0697
4060833454132,1.9111697226762772},height_median=0.6272794231772423,height_range={0.051077
58194208145,9.745632871985435},length=0.47168338233916024,length_95%_HPD={2.649489397299
476E-
5,1.4203613996505737},length_median=0.3149830996990204,length_range={2.649489397299476E-
5,12.299864768981934},posterior=0.7597450980392156,rate=0.007678735056802983,rate_95%_HPD=
{2.6755148347227947E-
6,0.0278654488111},rate_median=0.003443898609485,rate_range={2.6755148347227947E-
6,0.4509057365537}]:0.43546850234270096,(52[&height=2.4721074696148495E-
7,height_95%_HPD={0.0,5.960464477539062E-7},height_median=2.0116567611694336E-
7,height_range={0.0,4.451721906661987E-
6},length=0.8073241453248915,length_95%_HPD={1.7965949155041017E-
5,2.254528284072876},length_median=0.606083333492279,length_range={1.7965949155041017E-
5,12.281233787536621},rate=0.002185796805840096,rate_95%_HPD={1.0806042517381204E-
7,0.008244122938569},rate_median=8.193086938461485E-4,rate_range={1.0806042517381204E-

7,0.2341439537461}]:0.47974107414484024,(83[&height=2.4763872038331065E-
7,height_95%_HPD={0.0,6.258487701416016E-7},height_median=2.0116567611694336E-
7,height_range={0.0,4.451721906661987E-
6},length=0.6482717275471973,length_95%_HPD={2.6966165478370385E-
7,2.164388656616211},length_median=0.3743433803319931,length_range={2.6966165478370385E-
7,13.522167205810547},rate=0.002708559926226166,rate_95%_HPD={7.876867876853693E-
8,0.0104832955415},rate_median=9.248259903928218E-4,rate_range={7.876867876853693E-
8,0.3894294707639}]:0.2279028408229351,84[&height=2.4830390487063684E-
7,height_95%_HPD={0.0,6.258487701416016E-7},height_median=2.0302832126617432E-
7,height_range={0.0,7.033348083496094E-
6},length=0.6716179232010213,length_95%_HPD={2.6966165478370385E-
7,2.281940221786499},length_median=0.39048829674720764,length_range={2.6966165478370385E-
7,25.434600830078125},rate=0.0027022537359390054,rate_95%_HPD={1.6543030374190867E-
7,0.01046551261843},rate_median=9.395807509336216E-4,rate_range={1.6543030374190867E-
7,0.1855306335312}]:0.22790283896028996)[&height=0.3916868637742596,height_95%_HPD={2.9802
32238769531E-
7,1.2978090271353722},height_median=0.22790304198861122,height_range={2.980232238769531E-
7,6.4459967613220215},length=0.7055985357113485,length_95%_HPD={3.244776598876342E-
5,2.423671007156372},length_median=0.3974562883377075,length_range={3.244776598876342E-
5,13.535701751708984},posterior=0.1845686274509804,rate=0.00269170235565633,rate_95%_HPD={
4.9214668266926E-7,0.01060059803995},rate_median=9.474686846405114E-
4,rate_range={4.9214668266926E-
7,0.1488688353348}]:0.25183823332190514)[&height=0.6010240675242283,height_95%_HPD={0.0072
45197892189026,1.5189574360847473},height_median=0.47974127531051636,height_range={0.0072
45197892189026,5.891970187425613},length=0.6025434968922116,length_95%_HPD={2.7727803171
728738E-
5,1.6427913904190063},length_median=0.44980067014694214,length_range={2.7727803171728738E-
5,11.322325706481934},posterior=0.12594117647058822,rate=0.002737575703247961,rate_95%_HPD
={2.3862814541036037E-
7,0.01072895244046},rate_median=0.001074439260421,rate_range={2.3862814541036037E-
7,0.09740761549281}]:0.5830066502094269)[&height=1.2539525094500343,height_95%_HPD={0.2861
9564697146416,2.705846130847931},height_median=1.0627479255199432,height_range={0.0855507
3104798794,12.84169340133667},length=0.6813175010660505,length_95%_HPD={0.00294249108992
517,2.0658230781555176},length_median=0.43931037187576294,length_range={9.22666396945715E-
4,19.139440536499023},posterior=0.18998039215686274,rate=0.001811885960095173,rate_95%_HPD
={2.3456213301104275E-7,0.007199443025528},rate_median=7.463503488070004E-
4,rate_range={2.3456213301104275E-
7,0.06190651906612}]:0.362250910140574,60[&height=2.466700045827155E-
7,height_95%_HPD={0.0,5.960464477539062E-7},height_median=2.0116567611694336E-
7,height_range={0.0,4.451721906661987E-
6},length=1.6362279107519546,length_95%_HPD={2.8909562388435006E-
5,4.3683271408081055},length_median=1.2742029428482056,length_range={2.8909562388435006E-
5,24.25295066833496},rate=0.0013079273547278035,rate_95%_HPD={1.0600771855290261E-
7,0.0048816562759},rate_median=5.293465955171939E-4,rate_range={1.0600771855290261E-

7,0.09752577670164}]:1.424998634494841)[&height=1.7145363559072893,height_95%_HPD={0.41305963695049286,3.8610999584198},height_median=1.4249988356605172,height_range={0.16875039786100388,15.098479330539703},length=0.9832965623538209,length_95%_HPD={7.532590534538031E-4,3.1528186798095703},length_median=0.6093067228794098,length_range={7.532590534538031E-4,10.41134262084961},posterior=0.1168235294117647,rate=0.00162129682384497 3,rate_95%_HPD={4.8674637720947E-7,0.006056436261546},rate_median=6.361674327698228E-4,rate_range={4.8674637720947E-7,0.07498338592229}]:3.9005387565121055,((((((5[&height=2.4717273766095875E-7,height_95%_HPD={0.0,5.960464477539062E-7},height_median=2.0116567611694336E-7,height_range={0.0,4.649162292480469E-6},length=0.543965588116099,length_95%_HPD={8.600176079198718E-4,1.51902174949646},length_median=0.3961044251918793,length_range={3.520053287502378E-4,9.630008697509766},rate=0.002070205748383476,rate_95%_HPD={5.098980076433578E-8,0.007993870549142},rate_median=7.932780313937729E-4,rate_range={5.098980076433578E-8,0.2036443632653}]:0.25613532215356827,72[&height=2.47130661727633E-7,height_95%_HPD={0.0,5.960464477539062E-7},height_median=2.0116567611694336E-7,height_range={0.0,4.410743713378906E-6},length=0.43953725158565865,length_95%_HPD={2.440322896291036E-5,1.3192352056503296},length_median=0.294712632894516,length_range={2.440322896291036E-5,10.572267532348633},rate=0.002369657858007188,rate_95%_HPD={7.530627029690806E-8,0.009089204577366},rate_median=8.398538566642869E-4,rate_range={7.530627029690806E-8,0.5734331662661}]:0.25613532215356827)[&height=0.35999269535465606,height_95%_HPD={8.60169529914856E-4,1.0397337973117828},height_median=0.2561355233192444,height_range={8.60169529914856E-4,7.538656383752823},length=0.36271556513167796,length_95%_HPD={2.2300459022517316E-5,1.1507341861724854},length_median=0.23600617051124573,length_range={2.2300459022517316E-5,4.079501152038574},posterior=0.11680392156862746,rate=0.0026633179373423163,rate_95%_HPD={3.851153969350472E-7,0.0102816830842},rate_median=9.109858021347861E-4,rate_range={3.851153969350472E-7,0.2303494449812}]:0.7821806818246841,(((9[&height=2.471384511551935E-7,height_95%_HPD={0.0,5.960464477539062E-7},height_median=2.0116567611694336E-7,height_range={0.0,4.351139068603516E-6},length=0.44226448769034743,length_95%_HPD={2.440322896291036E-5,1.3349884748458862},length_median=0.2974151372909546,length_range={2.440322896291036E-5,10.714654922485352},rate=0.002350473924295527,rate_95%_HPD={1.5325665618718396E-7,0.009097416606719},rate_median=8.454331524683225E-4,rate_range={1.5325665618718396E-7,0.2743817365333}]:0.15268422290682793,11[&height=2.471417600875112E-7,height_95%_HPD={0.0,5.960464477539062E-7},height_median=2.0116567611694336E-7,height_range={0.0,4.351139068603516E-6},length=0.4416585357097175,length_95%_HPD={3.642965020844713E-5,1.3334578275680542},length_median=0.29663312435150146,length_range={3.642965020844713E-5,12.283110618591309},rate=0.0022675800145767425,rate_95%_HPD={2.1712057440246402E-7,0.008712083548566},rate_median=8.384679687041738E-4,rate_range={2.1712057440246402E-7,0.2805423938407}]:0.15268422290682793)[&height=0.2520296073410448,height_95%_HPD={3.6474

3173122406E-
5,0.8211547397077084},height_median=0.15268442407250404,height_range={3.64743173122406E-
5,6.640637308359146},length=0.41680722851409124,length_95%_HPD={1.3792949857815984E-
6,1.2920185327529907},length_median=0.26806357502937317,length_range={1.3792949857815984E-
6,7.570865631103516},posterior=0.18805882352941178,rate=0.002359129156172557,rate_95%_HPD=
{1.3733250199885108E-7,0.009178473583436},rate_median=8.266124724786548E-
4,rate_range={1.3733250199885108E-
7,0.1897292697089}]:0.2814148375764489,89[&height=2.4714042307011244E-
7,height_95%_HPD={0.0,5.960464477539062E-7},height_median=2.0116567611694336E-
7,height_range={0.0,4.649162292480469E-
6},length=0.7125455508431621,length_95%_HPD={0.0018676234176382422,2.0128183364868164},len
gth_median=0.5097618103027344,length_range={9.779445827007294E-
4,13.505781173706055},rate=0.002564591707888752,rate_95%_HPD={3.9544381839264615E-
8,0.01005724137207},rate_median=9.194867666078274E-4,rate_range={3.9544381839264615E-
8,0.205338316533}]:0.43409906048327684)[&height=0.5605732666146411,height_95%_HPD={0.01189
2572045326233,1.462842596694827},height_median=0.43409926164895296,height_range={0.011892
572045326233,6.327918857336044},length=0.3266370156221266,length_95%_HPD={1.326109922956
6753E-
4,1.006722092628479},length_median=0.21090225130319595,length_range={1.3261099229566753E-
4,3.720400094985962},posterior=0.04062745098039216,rate=0.0026355379406164093,rate_95%_HPD
={5.156074008303956E-7,0.01021521899863},rate_median=9.132397068642663E-
4,rate_range={5.156074008303956E-
7,0.08996097818984}]:0.25954196066595614,51[&height=2.47152298298965E-
7,height_95%_HPD={0.0,5.960464477539062E-7},height_median=2.0116567611694336E-
7,height_range={0.0,4.827976226806641E-
6},length=0.7238300964053334,length_95%_HPD={0.005428623408079147,1.863000750541687},lengt
h_median=0.560649186372757,length_range={1.5209068078547716E-
4,11.477882385253906},rate=0.020137262558376254,rate_95%_HPD={2.4166917406078815E-
7,0.06682237752367},rate_median=0.011364745293415,rate_range={2.4166917406078815E-
7,0.4629598004592}]:0.693641021149233)[&height=0.8196712009047828,height_95%_HPD={0.086842
1345949173,1.8501830101013184},height_median=0.6936412223149091,height_range={0.078524588
25800568,6.492647767066956},length=0.296475336272231,length_95%_HPD={5.656351568177342E-
4,0.9833303093910217},length_median=0.17990366369485855,length_range={5.656351568177342E-
4,3.742929697036743},posterior=0.021333333333333333,rate=0.0027330914876841673,rate_95%_HP
D={2.508049965354067E-6,0.009171738359812},rate_median=9.080758176061221E-
4,rate_range={2.508049965354067E-
6,0.2077893139246}]:0.3446749828290194)[&height=1.252900297473333,height_95%_HPD={0.209477
82881557941,2.797183394432068},height_median=1.0383162051439285,height_range={0.113865256
30950928,14.183157205581665},length=1.0248869504328162,length_95%_HPD={0.004325037356466
055,2.703247547149658},length_median=0.7917295694351196,length_range={4.713477028417401E-
5,10.899117469787598},posterior=0.7155294117647059,rate=0.004097629078256124,rate_95%_HPD=
{1.3007031365432633E-
6,0.01388390997418},rate_median=0.0021177052965160002,rate_range={1.3007031365432633E-
6,0.1806874364217}]:0.9577625077217817,(26[&height=2.4725470656868116E-

7,height_95%_HPD={0.0,5.960464477539062E-7},height_median=2.086162567138672E-
7,height_range={0.0,4.0531158447265625E-
6},length=2.1074125519912057,length_95%_HPD={8.562034781789407E-
5,4.776116847991943},length_median=1.812217116355896,length_range={8.562034781789407E-
5,16.553251266479492},rate=0.0012181783378842643,rate_95%_HPD={2.916402482267245E-
8,0.004449855035096},rate_median=5.64392325594493E-4,rate_range={2.916402482267245E-
8,0.1266503969846}]:0.536111444234848,90[&height=2.477436641096993E-
7,height_95%_HPD={0.0,6.032641977071762E-7},height_median=2.0116567611694336E-
7,height_range={0.0,4.76837158203125E-
6},length=0.864008680173369,length_95%_HPD={8.562034781789407E-
5,2.8168208599090576},length_median=0.5191584229469299,length_range={8.562034781789407E-
5,20.971757888793945},rate=0.003141914190447185,rate_95%_HPD={8.16429491291081E-
8,0.01204344448516},rate_median=0.0010184754405085,rate_range={8.16429491291081E-
8,0.3589614058276}]:0.5361114516854286)[&height=0.7320660638708097,height_95%_HPD={8.57710
8383178711E-
5,2.0512136220932007},height_median=0.5361116528511047,height_range={8.577108383178711E-
5,6.317795179784298},length=1.4542520263246748,length_95%_HPD={7.850311521906406E-
5,3.3066816329956055},length_median=1.275156855583191,length_range={7.850311521906406E-
5,9.361625671386719},posterior=0.1338235294117647,rate=0.0014148794741342201,rate_95%_HPD=
{1.751785081814366E-7,0.005114695095742},rate_median=5.924432684969106E-
4,rate_range={1.751785081814366E-
7,0.1045454900989}]:1.4599670600146055)[&height=2.2774208717163953,height_95%_HPD={0.58792
00398921967,4.698449518531561},height_median=1.9960787128657103,height_range={0.290704071
52175903,15.341421484947205},length=2.2262381559891664,length_95%_HPD={0.236165612936019
9,5.108704090118408},length_median=1.8632516264915466,length_range={0.0027185450308024883,
18.23973274230957},posterior=0.46098039215686276,rate=0.011742874247402063,rate_95%_HPD={2
.187823762466656E-
6,0.03385878673919},rate_median=0.007976704462142999,rate_range={2.187823762466656E-
6,0.1876075025661}]:1.8331551102455705,(((((14[&height=2.47515440217209E-
7,height_95%_HPD={0.0,5.960464477539062E-7},height_median=2.0116567611694336E-
7,height_range={0.0,5.360692739486694E-
6},length=0.2564363936360699,length_95%_HPD={2.238323759229388E-
6,0.8496420383453369},length_median=0.15308238565921783,length_range={2.238323759229388E-
6,5.331845283508301},rate=0.002789231641588076,rate_95%_HPD={9.095542711955178E-
8,0.01099809638295},rate_median=9.497324453927568E-4,rate_range={9.095542711955178E-
8,0.2650166814483}]:0.09767816215753555,37[&height=2.474566953408469E-
7,height_95%_HPD={0.0,5.960464477539062E-7},height_median=2.0116567611694336E-
7,height_range={0.0,5.360692739486694E-
6},length=0.2593391355980234,length_95%_HPD={2.9597326829389203E-
6,0.8511673212051392},length_median=0.155746191740036,length_range={2.9597326829389203E-
6,6.198563575744629},rate=0.0027078575248913692,rate_95%_HPD={1.4614345782763677E-
7,0.01050340869241},rate_median=9.505214921181116E-4,rate_range={1.4614345782763677E-
7,0.2962410747876}]:0.09767816215753555)[&height=0.1718240928144052,height_95%_HPD={3.3378
60107421875E-

6,0.5853101909160614},height_median=0.09767836332321167,height_range={3.337860107421875E-
6,2.8579303938895464},length=0.2554383045429391,length_95%_HPD={1.2102433174732141E-
5,0.8392080664634705},length_median=0.14880649745464325,length_range={1.2102433174732141E-
5,5.5938496589660645},posterior=0.2198235294117647,rate=0.0026776199974308836,rate_95%_HPD
={3.069387418740521E-7,0.01063006520767},rate_median=9.138894988219287E-
4,rate_range={3.069387418740521E-
7,0.2335210473887}]:0.32332874834537506,(38[&height=2.4750370952055095E-
7,height_95%_HPD={0.0,5.960464477539062E-7},height_median=2.0116567611694336E-
7,height_range={0.0,5.364418029785156E-
6},length=0.2589744251213709,length_95%_HPD={1.0524116987653542E-
5,0.864109218120575},length_median=0.153166763484478,length_range={8.001720743777696E-
6,6.30653190612793},rate=0.002906131324389374,rate_95%_HPD={2.1304560768081972E-
8,0.01099258246791},rate_median=9.280641986572828E-4,rate_range={2.1304560768081972E-
8,0.2565476437427}]:0.10286343097686768,74[&height=2.474801214617071E-
7,height_95%_HPD={0.0,5.960464477539062E-7},height_median=2.0116567611694336E-
7,height_range={0.0,5.364418029785156E-
6},length=0.2597063019346807,length_95%_HPD={2.238323759229388E-
6,0.85463547706604},length_median=0.15571413189172745,length_range={2.238323759229388E-
6,6.167153358459473},rate=0.002733227419830982,rate_95%_HPD={3.2852193835147626E-
8,0.01060246683919},rate_median=9.337755430989675E-4,rate_range={3.2852193835147626E-
8,0.2995346985538}]:0.10286343097686768)[&height=0.1739004616219489,height_95%_HPD={1.1505
791917443275E-
5,0.5829939320683479},height_median=0.10286363214254379,height_range={1.1505791917443275E-
5,3.0189179703593254},length=0.25005969713238935,length_95%_HPD={6.861974725325126E-
6,0.8286697268486023},length_median=0.15043598413467407,length_range={6.861974725325126E-
6,5.460632801055908},posterior=0.22111764705882353,rate=0.0027776002230805396,rate_95%_HPD
={5.608309698689119E-8,0.01083966848396},rate_median=9.438770360990138E-
4,rate_range={5.608309698689119E-
8,0.1671458404329}]:0.31814347952604294)[&height=0.5494168556493971,height_95%_HPD={0.0239
8321032524109,1.4072664976119995},height_median=0.42100711166858673,height_range={0.00331
39586448669434,9.644647657871246},length=1.5661930315471606,length_95%_HPD={0.1518407911
0622406,3.4655232429504395},length_median=1.3137047290802002,length_range={0.065470919013
02338,18.249868392944336},posterior=0.9999803921568627,rate=0.02345689803729355,rate_95%_H
PD={0.001251419440464,0.06455187775389},rate_median=0.01701795098548,rate_range={6.6350155
06457776E-5,0.3995724262743}]:1.3163223769515753,44[&height=2.474729758471669E-
7,height_95%_HPD={0.0,5.960464477539062E-7},height_median=2.0349398255348206E-
7,height_range={0.0,5.0067901611328125E-
6},length=1.3859125825369583,length_95%_HPD={5.369993232307024E-
6,3.6135141849517822},length_median=1.1426255702972412,length_range={5.369993232307024E-
6,21.13933563232422},rate=0.008079995488658212,rate_95%_HPD={3.416162411693773E-
8,0.02560660087076},rate_median=0.0049766453659755,rate_range={3.416162411693773E-
8,0.3442892518192}]:1.7373292851261795)[&height=2.0395714604813464,height_95%_HPD={0.37484
992668032646,4.313235878944397},height_median=1.737329488620162,height_range={0.227369621
39606476,21.13933753967285},length=0.99386624302544,length_95%_HPD={0.05075152590870857,2

.4793505668640137},length_median=0.7978019416332245,length_range={8.376411278732121E-
4,9.919349670410156},posterior=0.5112156862745098,rate=0.013308253414669787,rate_95%_HPD={
3.1415583129839055E-
6,0.03907978409117},rate_median=0.008305781922452501,rate_range={3.1415583129839055E-
6,0.5933757286049}]:0.6289730325806886,(56[&height=2.4794410008573374E-
7,height_95%_HPD={0.0,5.960464477539062E-7},height_median=2.0489096641540527E-
7,height_range={0.0,5.364418029785156E-
6},length=0.6050982622762265,length_95%_HPD={0.013226974755525589,1.6014004945755005},leng
th_median=0.45414818823337555,length_range={0.0037338323891162872,9.330904006958008},rate=
0.006381097651785964,rate_95%_HPD={1.9886644830393194E-
6,0.02271267467206},rate_median=0.0032594764523835,rate_range={1.9886644830393194E-
6,0.2436170714206}]:0.4544670321047306,57[&height=2.4792364066358603E-
7,height_95%_HPD={0.0,5.960464477539062E-7},height_median=2.0489096641540527E-
7,height_range={0.0,5.364418029785156E-
6},length=0.6027487688098975,length_95%_HPD={0.007275617681443691,1.5972654819488525},leng
th_median=0.4522750675678253,length_range={5.326837999746203E-
4,9.330904006958008},rate=0.001988878916359385,rate_95%_HPD={1.337841687211683E-
7,0.00773402493944},rate_median=7.818880853425294E-4,rate_range={1.337841687211683E-
7,0.1498048279548}]:0.4544670321047306)[&height=0.6055816215450962,height_95%_HPD={0.01586
3515436649323,1.6041916012763977},height_median=0.454467236995697,height_range={0.0037339
92576599121,9.330904722213745},length=1.8979137751827149,length_95%_HPD={0.2304726243019
104,4.363652229309082},length_median=1.6018496751785278,length_range={0.00345502211712300
78,22.465864181518555},posterior=0.9778823529411764,rate=0.014715661417927602,rate_95%_HPD
={2.1521745689132844E-
5,0.03837630585438},rate_median=0.010976785920675,rate_range={9.418597095079594E-
6,0.3269714090974}]:1.9118352842051536)[&height=2.817036132079689,height_95%_HPD={0.957322
6235806942,5.9356926046311855},height_median=2.3663025212008506,height_range={0.799035776
4065266,18.57369899749756},length=0.3255083422524833,length_95%_HPD={6.267844582907856E-
5,1.0523951053619385},length_median=0.19726307690143585,length_range={6.267844582907856E-
5,4.384806156158447},posterior=0.0476078431372549,rate=0.002842987930060116,rate_95%_HPD={
5.999681349371142E-7,0.01186872431863},rate_median=9.607485299230765E-
4,rate_range={5.999681349371142E-
7,0.09047240298356}]:0.4450459803920239,(22[&height=2.473018317334886E-
7,height_95%_HPD={0.0,5.960464477539062E-7},height_median=2.0489096641540527E-
7,height_range={0.0,5.0067901611328125E-
6},length=2.602735303280656,length_95%_HPD={0.29451677203178406,5.629993438720703},length_
median=2.2277963161468506,length_range={0.025125183165073395,25.579315185546875},rate=0.02
0720652632745355,rate_95%_HPD={3.343021365098674E-
6,0.05069454382834},rate_median=0.01639204398743,rate_range={3.343021365098674E-
6,0.3363266581085}]:1.7493967693299055,53[&height=2.472913137304275E-
7,height_95%_HPD={0.0,5.960464477539062E-7},height_median=2.0489096641540527E-
7,height_range={0.0,4.76837158203125E-
6},length=2.2170830595356192,length_95%_HPD={2.5937717873603106E-
4,4.944002628326416},length_median=1.9220168590545654,length_range={3.0177443477441557E-

5,22.009851455688477},rate=0.0025612029129568987,rate_95%_HPD={4.569276555057894E-
7,0.008197117530008},rate_median=0.0015155027333285,rate_range={4.569276555057894E-
7,0.2099592153468}]:1.7493967693299055)[&height=2.06064874826291,height_95%_HPD={0.2501718
8489437103,4.515459388494492},height_median=1.749396974220872,height_range={0.15013083815
574646,18.785772770643234},length=0.7519058597569622,length_95%_HPD={9.392816718900576E-
5,2.202728271484375},length_median=0.5178750157356262,length_range={9.392816718900576E-
5,9.76276969909668},posterior=0.23527450980392156,rate=0.0020794800523854426,rate_95%_HPD=
{7.358161812319412E-8,0.0081542426149},rate_median=7.129241572274625E-
4,rate_range={7.358161812319412E-
8,0.166163038679}]:1.0619515273720026)[&height=3.280631804538904,height_95%_HPD={1.0441577
13651657,6.710281729698181},height_median=2.8113485015928745,height_range={0.767734110355
3772,26.476551055908203},length=1.257823956438364,length_95%_HPD={0.13104909658432007,2.9
396779537200928},length_median=1.0466036200523376,length_range={0.001881119329482317,10.7
19432830810547},posterior=0.3935294117647059,rate=0.018265623784765785,rate_95%_HPD={1.792
5906046307609E-
6,0.05372721067673},rate_median=0.01238849372843,rate_range={1.7925906046307609E-
6,0.4366922057202}]:1.0178853215184063)[&height=4.430751315329789,height_95%_HPD={1.688028
6075174809,8.939947575330734},height_median=3.829233823111281,height_range={1.10777105391
02554,29.657969247549772},length=0.5766357804167335,length_95%_HPD={0.001577679991726875
5,1.5683830976486206},length_median=0.41558024287223816,length_range={1.9136694027110934E-
4,7.087251663208008},posterior=0.24790196078431373,rate=0.0024188972045725184,rate_95%_HPD
={4.92053787783499E-7,0.009640599160078},rate_median=8.777557496028033E-
4,rate_range={4.92053787783499E-
7,0.2464582761632}]:0.2638172230217606,((46[&height=2.4691911140534475E-
7,height_95%_HPD={0.0,6.034970283508301E-7},height_median=2.086162567138672E-
7,height_range={0.0,4.410743713378906E-
6},length=2.382553284076941,length_95%_HPD={8.701130282133818E-
4,5.820878982543945},length_median=1.977493941783905,length_range={8.701130282133818E-
4,27.949522018432617},rate=0.011931387923089435,rate_95%_HPD={1.4383232856520592E-
6,0.03449785385007},rate_median=0.0079803268651215,rate_range={1.4383232856520592E-
6,0.3930809722806}]:0.6803480591624975,93[&height=2.469654239566986E-
7,height_95%_HPD={0.0,5.960464477539062E-7},height_median=2.0116567611694336E-
7,height_range={0.0,4.738569259643555E-
6},length=0.689621540384596,length_95%_HPD={6.750145530531881E-
6,2.3329131603240967},length_median=0.39337001740932465,length_range={6.750145530531881E-
6,19.862882614135742},rate=0.003431248998334136,rate_95%_HPD={9.800194735773744E-
8,0.01335288601381},rate_median=0.0010755041247265,rate_range={9.800194735773744E-
8,0.2523352471706}]:0.6803480666130781)[&height=1.0202177740180047,height_95%_HPD={8.70406
6276550293E-
4,3.087731420993805},height_median=0.6803482677787542,height_range={8.704066276550293E-
4,10.771133244037628},length=2.005802470068685,length_95%_HPD={0.0016455637523904443,5.38
2798194885254},length_median=1.58112633228302,length_range={0.0016455637523904443,16.7568
98880004883},posterior=0.07262745098039215,rate=0.009162005965066656,rate_95%_HPD={6.27507
3876111687E-

7,0.0316228759727},rate_median=0.004985521040337,rate_range={6.275073876111687E-7,0.09838649110419}]:1.5681195901706815,50[&height=2.474535031002661E-7,height_95%_HPD={0.0,6.109476089477539E-7},height_median=2.086162567138672E-7,height_range={0.0,5.841255187988281E-6},length=2.4574040041749745,length_95%_HPD={4.620492982212454E-4,6.035974979400635},length_median=2.0298688411712646,length_range={4.620492982212454E-4,27.55666732788086},rate=0.002599482583590988,rate_95%_HPD={3.0627634152880074E-7,0.008497992183986},rate_median=0.0014490538682325,rate_range={3.0627634152880074E-7,0.1513222707677}]:2.248467649333179)[&height=2.6674752941187423,height_95%_HPD={0.38481971621513367,6.1503764390945435},height_median=2.2484678579494357,height_range={0.21021205931901932,15.423914402723312},length=2.3364298094589455,length_95%_HPD={7.362384931184351E-4,5.786710262298584},length_median=1.95863276720047,length_range={7.362384931184351E-4,15.342400550842285},posterior=0.08913725490196078,rate=0.003014739804552187,rate_95%_HPD={1.2784555429508112E-6,0.009299036121431},rate_median=0.0013755488687475,rate_range={1.2784555429508112E-6,0.1404317519328}]:1.8445831881836057)[&height=4.729853274498662,height_95%_HPD={1.8758399486541748,9.638536185026169},height_median=4.093051046133041,height_range={1.6492122411727905,19.097019970417023},length=1.1476313998611882,length_95%_HPD={0.017127107828855515,2.965177297592163},length_median=0.9067465662956238,length_range={0.017127107828855515,8.977766036987305},posterior=0.04782352941176471,rate=0.00785240460715268,rate_95%_HPD={2.6878405481916512E-5,0.0259194849763},rate_median=0.004512104369597,rate_range={2.6878405481916512E-5,0.1472740025418}]:0.76890879124403,((((((((8[&height=2.4652866878840557E-7,height_95%_HPD={0.0,5.885958671569824E-7},height_median=2.0116567611694336E-7,height_range={0.0,4.351139068603516E-6},length=0.252552863403564,length_95%_HPD={5.745497674070066E-6,0.8006139397621155},length_median=0.15785431116819382,length_range={5.745497674070066E-6,5.691987991333008},rate=0.0027934844582238635,rate_95%_HPD={5.191344849384201E-8,0.01098719420925},rate_median=9.472657876703722E-4,rate_range={5.191344849384201E-8,0.4871485922359}]:0.09674682654440403,76[&height=2.465188549538153E-7,height_95%_HPD={0.0,5.923211574554443E-7},height_median=2.0116567611694336E-7,height_range={0.0,4.500150680541992E-6},length=0.253745079569076,length_95%_HPD={5.745497674070066E-6,0.8057297468185425},length_median=0.15919361263513565,length_range={5.745497674070066E-6,7.735491752624512},rate=0.0027414077280819974,rate_95%_HPD={4.9920590198430666E-8,0.01078461546654},rate_median=9.563797942902919E-4,rate_range={4.9920590198430666E-8,0.3518017189826}]:0.09674682654440403)[&height=0.16010636652186763,height_95%_HPD={5.891546607017517E-6,0.5190066695213318},height_median=0.09674702771008015,height_range={5.891546607017517E-6,4.677127093076706},length=0.23207756906818974,length_95%_HPD={1.2032434142383863E-6,0.742656409740448},length_median=0.1434306800365448,length_range={1.2032434142383863E-6,6.042505741119385},posterior=0.16680392156862744,rate=0.002903775436123753,rate_95%_HPD={1.9716407592069555E-8,0.01169366104664},rate_median=9.524251866248995E-4,rate_range={1.9716407592069555E-

8,0.2019942488171}]:0.1115134209394455,69[&height=2.465844767821003E-
7,height_95%_HPD={0.0,5.904585123062134E-7},height_median=2.0116567611694336E-
7,height_range={0.0,4.351139068603516E-
6},length=0.25565996613578235,length_95%_HPD={1.3280669008963741E-
6,0.8051460981369019},length_median=0.1621181219816208,length_range={1.3280669008963741E-
6,7.0010833740234375},rate=0.002766511777004112,rate_95%_HPD={8.77143031425375E-
8,0.01073777436762},rate_median=9.172850439954502E-4,rate_range={8.77143031425375E-
8,0.3358352200481}]:0.20826024748384953)[&height=0.27892994031832624,height_95%_HPD={0.002
074768766760826,0.7759727835655212},height_median=0.20826044864952564,height_range={5.852
263420820236E-
4,2.470942199230194},length=0.2189720643856338,length_95%_HPD={3.097074295510538E-
5,0.6940807104110718},length_median=0.13763795793056488,length_range={3.097074295510538E-
5,5.420262336730957},posterior=0.08449019607843138,rate=0.0029142353385463054,rate_95%_HPD
={2.9875871046028733E-7,0.01035295450108},rate_median=9.380909601608554E-
4,rate_range={2.9875871046028733E-
7,0.213668392026}]:0.08717027492821217,70[&height=2.464507860319524E-
7,height_95%_HPD={0.0,5.923211574554443E-7},height_median=2.0116567611694336E-
7,height_range={0.0,4.425644874572754E-
6},length=0.2555628014419752,length_95%_HPD={1.3280669008963741E-
6,0.8106386065483093},length_median=0.1617959588766098,length_range={1.3280669008963741E-
6,7.070281982421875},rate=0.0027992933992602963,rate_95%_HPD={2.120677086866677E-
8,0.01081112863369},rate_median=9.630386121868889E-4,rate_range={2.120677086866677E-
8,0.329932467544}]:0.2954305224120617)[&height=0.3775709104755852,height_95%_HPD={0.010857
291519641876,0.9598474726080894},height_median=0.2954307235777378,height_range={0.0016177
669167518616,4.120108485221863},length=0.21864609540504576,length_95%_HPD={4.70301420136
8205E-
5,0.7171390056610107},length_median=0.13467150926589966,length_range={4.703014201368205E-
5,2.723543882369995},posterior=0.12809803921568627,rate=0.002974846599280329,rate_95%_HPD=
{3.951086929289541E-
7,0.01151429861577},rate_median=0.001041052224241,rate_range={3.951086929289541E-
7,0.1957955745641}]:0.17184560000896454,39[&height=2.4655520338103763E-
7,height_95%_HPD={0.0,5.923211574554443E-7},height_median=2.0116567611694336E-
7,height_range={0.0,4.500150680541992E-
6},length=0.34621004499001234,length_95%_HPD={0.0016022301279008389,0.990752637386322},len
gth_median=0.24585731327533722,length_range={4.6273457701317966E-
4,7.287601470947266},rate=0.002455657065442192,rate_95%_HPD={2.951221139123222E-
8,0.009863547154229},rate_median=8.797659601762504E-4,rate_range={2.951221139123222E-
8,0.1294036363847}]:0.46727612242102623)[&height=0.5791847077884036,height_95%_HPD={0.0489
9035394191742,1.3923905938863754},height_median=0.46727632358670235,height_range={0.01618
303544819355,7.735492169857025},length=0.36801241555145464,length_95%_HPD={5.95473567955
1959E-
4,1.0541983842849731},length_median=0.25659000873565674,length_range={5.5307787988567725E-
5,7.901201248168945},posterior=0.6013725490196078,rate=0.0023949608091513967,rate_95%_HPD=
{1.4614345782763677E-7,0.009378550061814},rate_median=8.784490763281371E-

4,rate_range={4.923438724055647E-
8,0.2570893297058}]:0.3096619211137295,40[&height=2.462527028183995E-
7,height_95%_HPD={0.0,5.960464477539062E-7},height_median=2.0116567611694336E-
7,height_range={0.0,4.291534423828125E-
6},length=0.8818069604629799,length_95%_HPD={6.342322012642398E-
5,2.096796751022339},length_median=0.7273845076560974,length_range={6.342322012642398E-
5,14.004534721374512},rate=0.0016098701114645668,rate_95%_HPD={5.148120407103668E-
8,0.006023523104232},rate_median=6.777235975244564E-4,rate_range={5.148120407103668E-
8,0.1083076202436}]:0.7769380435347557)[&height=0.9324145131987429,height_95%_HPD={0.12298
812717199326,2.113194741308689},height_median=0.7769382447004318,height_range={0.04776825
01077652,10.10206152126193},length=0.5698845700037103,length_95%_HPD={2.5583163369446993
E-
4,1.6025536060333252},length_median=0.40468844771385193,length_range={1.1946109589189291E-
4,13.053903579711914},posterior=0.465,rate=0.002106114442416963,rate_95%_HPD={1.97990044298
4039E-7,0.007974886289671},rate_median=7.796630945891092E-
4,rate_range={1.979900442984039E-
7,0.4045805701179}]:0.7218180410563946,(((42[&height=2.4666299893673494E-
7,height_95%_HPD={0.0,5.960464477539062E-7},height_median=2.0116567611694336E-
7,height_range={0.0,3.814697265625E-
6},length=0.7524822418888707,length_95%_HPD={5.934493674430996E-
5,2.1142313480377197},length_median=0.5369911193847656,length_range={5.934493674430996E-
5,26.550485610961914},rate=0.0059616654622895995,rate_95%_HPD={7.684839306116805E-
7,0.02122633892871},rate_median=0.0028536481101885,rate_range={7.684839306116805E-
7,0.2951832707617}]:0.3540215853136033,54[&height=2.467800030493819E-
7,height_95%_HPD={0.0,5.960464477539062E-7},height_median=2.0116567611694336E-
7,height_range={0.0,4.291534423828125E-
6},length=0.5350291716711661,length_95%_HPD={2.0137913452344947E-
5,1.7082120180130005},length_median=0.32575367391109467,length_range={2.0137913452344947E-
5,27.30671501159668},rate=0.0022848144982171997,rate_95%_HPD={7.944176254915269E-
8,0.008844262277156},rate_median=8.13971208278438E-4,rate_range={7.944176254915269E-
8,0.2332027732435}]:0.3540215853136033)[&height=0.5006739508496306,height_95%_HPD={0.00325
04722476005554,1.4303722381591797},height_median=0.3540217864792794,height_range={2.74658
203125E-
4,23.086284399032593},length=0.49241054138939155,length_95%_HPD={2.9098733648424968E-
5,1.5956183671951294},length_median=0.30254779756069183,length_range={2.9098733648424968E-
5,6.664549827575684},posterior=0.12611764705882353,rate=0.002166101117797774,rate_95%_HPD
={7.0402731482196E-8,0.008540894165366},rate_median=8.012029747230764E-
4,rate_range={7.0402731482196E-
8,0.08696126973229}]:0.1797858497593552,68[&height=2.4672344344190945E-
7,height_95%_HPD={0.0,5.960464477539062E-7},height_median=2.0116567611694336E-
7,height_range={0.0,4.649162292480469E-
6},length=0.530593673676812,length_95%_HPD={1.373563372908393E-
5,1.6967010498046875},length_median=0.32305824756622314,length_range={1.373563372908393E-
5,37.385597229003906},rate=0.0022791097839760686,rate_95%_HPD={8.089686270905001E-

9,0.008625082153381},rate_median=8.261766345530478E-4,rate_range={8.089686270905001E-
9,0.3639332399039}]:0.5338074350729585)[&height=0.7007011855331433,height_95%_HPD={0.02371
343970298767,1.7834302634000778},height_median=0.5338076362386346,height_range={0.0140093
80713105202,13.47170677781105},length=0.5224784626417546,length_95%_HPD={2.7999692247249
186E-
4,1.6673506498336792},length_median=0.33391548693180084,length_range={2.7999692247249186E-
4,13.428997039794922},posterior=0.0852156862745098,rate=0.0021817111997430843,rate_95%_HPD
={5.891820613004768E-7,0.007790302863018},rate_median=8.187007381106198E-
4,rate_range={5.891820613004768E-
7,0.1418403175719}]:0.37693881522864103,(85[&height=2.468967899399193E-
7,height_95%_HPD={0.0,5.960464477539062E-7},height_median=2.0116567611694336E-
7,height_range={0.0,3.814697265625E-
6},length=0.5684401008221499,length_95%_HPD={1.373563372908393E-
5,1.8064314126968384},length_median=0.3452030271291733,length_range={1.373563372908393E-
5,24.619707107543945},rate=0.0025347073846866152,rate_95%_HPD={5.6779625651347636E-
8,0.009964455885336},rate_median=9.147859112053227E-4,rate_range={5.6779625651347636E-
8,0.2209414171158}]:0.21077315136790276,86[&height=2.468156777026439E-
7,height_95%_HPD={0.0,5.960464477539062E-7},height_median=2.0116567611694336E-
7,height_range={0.0,4.425644874572754E-
6},length=0.46572360432656795,length_95%_HPD={1.207115565193817E-
5,1.512920618057251},length_median=0.28016260266304016,length_range={9.903304089675657E-
6,15.552508354187012},rate=0.002918081906543219,rate_95%_HPD={2.6516381461437156E-
8,0.01166341324898},rate_median=9.676135323822344E-4,rate_range={2.6516381461437156E-
8,0.226700502853}]:0.21077315136790276)[&height=0.3417160158329466,height_95%_HPD={5.85876
4052391052E-
5,1.145268201828003},height_median=0.21077335253357887,height_range={5.858764052391052E-
5,3.839891256764531},length=0.5295343508912115,length_95%_HPD={1.6196852084249258E-
4,1.674331784248352},length_median=0.34184837341308594,length_range={1.6196852084249258E-
4,10.678772926330566},posterior=0.08147058823529411,rate=0.002680174765216862,rate_95%_HPD
={1.6792367955017123E-7,0.01058555223965},rate_median=8.94459116991335E-
4,rate_range={1.6792367955017123E-
7,0.1964400744042}]:0.6999730989336967)[&height=1.1066151863198912,height_95%_HPD={0.09115
918725728989,2.5368222892284393},height_median=0.9107464514672756,height_range={0.0683667
3244833946,12.62490525841713},length=0.6377723567552077,length_95%_HPD={2.09746518521569
67E-
4,1.8736157417297363},length_median=0.4356033205986023,length_range={2.0974651852156967E-
4,10.595480918884277},posterior=0.14349019607843136,rate=0.0020167583605464738,rate_95%_HP
D={3.334273850086967E-8,0.007599156310886},rate_median=7.207461932355039E-
4,rate_range={3.334273850086967E-
8,0.1556931138696}]:0.5880098342895508)[&height=1.8096696083610702,height_95%_HPD={0.40517
04928278923,3.941213011741638},height_median=1.4987562857568264,height_range={0.193782918
15519333,37.3855984210968},length=1.401995036283326,length_95%_HPD={6.637330516241491E-
4,3.7280404567718506},length_median=1.048846185207367,length_range={6.637330516241491E-
4,25.97447395324707},posterior=0.5844705882352941,rate=0.003831817791021074,rate_95%_HPD={

1.781778067418175E-
7,0.01301551759396},rate_median=0.0020314357595115,rate_range={1.781778067418175E-
7,0.1741076635443}]:-0.0031576864421367645,43[&height=2.47308733376075E-
7,height_95%_HPD={0.0,5.960464477539062E-7},height_median=2.0279549062252045E-
7,height_range={0.0,5.841255187988281E-
6},length=0.6836648863320143,length_95%_HPD={5.369993232307024E-
6,2.2926759719848633},length_median=0.38211779296398163,length_range={5.369993232307024E-
6,23.696331024169922},rate=0.00312971358323962,rate_95%_HPD={6.116312087757834E-
9,0.01236226775378},rate_median=0.001012596952512,rate_range={6.116312087757834E-
9,0.4481429714472}]:1.495598396519199)[&height=1.7642185800717942,height_95%_HPD={0.373683
1471323967,3.870253473520279},height_median=1.4955985993146896,height_range={0.2895988239
906728,10.572630882263184},length=1.2825578993685436,length_95%_HPD={0.00618731277063489,
3.424485206604004},length_median=0.967104971408844,length_range={0.00618731277063489,15.04
0656089782715},posterior=0.11884313725490196,rate=0.003801020063408786,rate_95%_HPD={9.648
167725413007E-
6,0.01292435979252},rate_median=0.001825322423451,rate_range={9.648167725413007E-
6,0.1878205550839}]:0.8922093182482058,(24[&height=2.4701256845049763E-
7,height_95%_HPD={0.0,5.960464477539062E-7},height_median=2.0116567611694336E-
7,height_range={0.0,4.291534423828125E-
6},length=1.1046667067778295,length_95%_HPD={4.982004757039249E-
4,3.0620851516723633},length_median=0.8090022504329681,length_range={4.982004757039249E-
4,16.57581901550293},rate=0.004183381889352922,rate_95%_HPD={5.139121296528549E-
7,0.01422760283777},rate_median=0.0023034943523379996,rate_range={5.139121296528549E-
7,0.1849660087848}]:0.7735686376690865,41[&height=2.4688667536909134E-
7,height_95%_HPD={0.0,5.960464477539062E-7},height_median=2.0116567611694336E-
7,height_range={0.0,4.291534423828125E-
6},length=1.0862287371780288,length_95%_HPD={2.0550515910144895E-
4,2.920142412185669},length_median=0.8159583210945129,length_range={2.0550515910144895E-
4,22.424211502075195},rate=0.0016476957994285349,rate_95%_HPD={2.0695422626376435E-
7,0.006114166817322},rate_median=6.85639642764296E-4,rate_range={2.0695422626376435E-
7,0.06874708215802}]:0.7735686376690865)[&height=1.024336637008819,height_95%_HPD={0.05976
6883961856365,2.718460887670517},height_median=0.7735688388347626,height_range={0.0332989
3946647644,12.90456673502922},length=1.13591062378651,length_95%_HPD={5.816250268253498E-
5,3.170011520385742},length_median=0.8125146627426147,length_range={5.816250268253498E-
5,21.6226806640625},posterior=0.6775882352941176,rate=0.00425994847574652,rate_95%_HPD={3.6
591433921074867E-
7,0.01528151472168},rate_median=0.002042017447213,rate_range={3.6591433921074867E-
7,0.1927781633813}]:1.6142390787281329)[&height=2.727962314532123,height_95%_HPD={0.594239
7490143776,5.630384474992752},height_median=2.3878079175628955,height_range={0.4417550414
800644,17.678239226341248},length=0.7823831670919876,length_95%_HPD={8.421457605436444E-
4,2.520306348800659},length_median=0.5038408041000366,length_range={8.421457605436444E-
4,7.655479907989502},posterior=0.029705882352941176,rate=0.0014775229956160408,rate_95%_HP
D={1.4373647173760749E-7,0.005976864626562},rate_median=5.770656290299257E-
4,rate_range={1.4373647173760749E-

7,0.0215351043428}]:1.0080256939254468,((((((((15[&height=2.471335966461373E-
7,height_95%_HPD={0.0,5.960464477539062E-7},height_median=2.0116567611694336E-
7,height_range={0.0,4.76837158203125E-
6},length=0.461915625656087,length_95%_HPD={1.6045287338783965E-
5,1.6416144371032715},length_median=0.241468645632267,length_range={1.6045287338783965E-
5,18.02826690673828},rate=0.002321793249287187,rate_95%_HPD={8.233513716399821E-
8,0.009017385257252},rate_median=8.300965345458635E-4,rate_range={8.233513716399821E-
8,0.1997641636024}]:0.15136631950736046,49[&height=2.472818980700541E-
7,height_95%_HPD={0.0,5.960464477539062E-7},height_median=2.0116567611694336E-
7,height_range={0.0,4.541128873825073E-
6},length=0.45489734155979217,length_95%_HPD={5.062662694399478E-
6,1.5957683324813843},length_median=0.23783309757709503,length_range={5.062662694399478E-
6,15.170856475830078},rate=0.002419778582146907,rate_95%_HPD={1.3289393649852225E-
7,0.009430400970661},rate_median=8.498243569136376E-4,rate_range={1.3289393649852225E-
7,0.3159503381102}]:0.15136631950736046)[&height=0.2659306034966247,height_95%_HPD={3.4436
583518981934E-
5,0.9519895426928997},height_median=0.15136652067303658,height_range={3.4436583518981934E-
5,2.989564925432205},length=0.47981899908941805,length_95%_HPD={3.730057505890727E-
4,1.7264866828918457},length_median=0.26237577199935913,length_range={3.730057505890727E-
4,6.179946422576904},posterior=0.02464705882352941,rate=0.002499576382073246,rate_95%_HPD=
{4.293748068116274E-6,0.009591946105444},rate_median=8.359981950386365E-
4,rate_range={4.293748068116274E-
6,0.08953374855251}]:0.10477911401540041,((63[&height=2.471475302788425E-
7,height_95%_HPD={0.0,5.960464477539062E-7},height_median=2.0116567611694336E-
7,height_range={0.0,4.850327968597412E-
6},length=0.45046524369616364,length_95%_HPD={2.936471901193727E-
6,1.573369026184082},length_median=0.2379777655005455,length_range={2.936471901193727E-
6,12.188590049743652},rate=0.0024488492205538255,rate_95%_HPD={7.705882390458714E-
8,0.009543090291302},rate_median=8.589563262435646E-4,rate_range={7.705882390458714E-
8,0.4073398910986}]:0.14765238901600242,65[&height=2.4707449168427137E-
7,height_95%_HPD={0.0,5.960464477539062E-7},height_median=2.0116567611694336E-
7,height_range={0.0,4.69014048576355E-
6},length=0.45625117485413835,length_95%_HPD={1.0528462553338613E-
5,1.6315877437591553},length_median=0.23501334339380264,length_range={1.0528462553338613E-
5,12.400381088256836},rate=0.0024826084294857246,rate_95%_HPD={7.153042737944895E-
9,0.009604309232628},rate_median=8.537590724952628E-4,rate_range={7.153042737944895E-
9,0.2656993023351}]:0.14765238901600242)[&height=0.29561296337002135,height_95%_HPD={2.362
7281188964844E-
4,1.031901404261589},height_median=0.14765259018167853,height_range={2.3627281188964844E-
4,8.236421763896942},length=0.5574720969414779,length_95%_HPD={2.0669717923738062E-
4,1.994941234588623},length_median=0.2904479205608368,length_range={2.0669717923738062E-
4,7.216686248779297},posterior=0.02456862745098039,rate=0.0021952042837172247,rate_95%_HPD
={3.845753897564462E-7,0.008858270710634},rate_median=8.089172173625519E-
4,rate_range={3.845753897564462E-

7,0.0853419291706}]:0.13357472280040383,87[&height=2.468959711628557E-
7,height_95%_HPD={0.0,5.960464477539062E-7},height_median=2.0116567611694336E-
7,height_range={0.0,3.814697265625E-
6},length=0.5366053014304168,length_95%_HPD={3.4602844607434236E-
6,1.876078724861145},length_median=0.2818083316087723,length_range={3.4602844607434236E-
6,27.022178649902344},rate=0.0028933641054995425,rate_95%_HPD={9.959200596419862E-
8,0.01119774207952},rate_median=9.385528613775261E-4,rate_range={9.959200596419862E-
8,0.2818893188236}]:0.28122711181640625)[&height=0.3726972454375672,height_95%_HPD={0.0237
37464100122452,1.1380467265844345},height_median=0.28122731298208237,height_range={0.0237
37464100122452,1.542750533670187},length=0.26945865222845566,length_95%_HPD={0.001093592
9603874683,0.7761330604553223},length_median=0.16173489391803741,length_range={0.00109359
29603874683,1.2083734273910522},posterior=0.0010784313725490195,rate=0.003577340481250932,
rate_95%_HPD={7.359166709516624E-5,0.01496411912245},rate_median=9.85943849280543E-
4,rate_range={7.359166709516624E-5,0.0278976720199}]:-
0.025081678293645382)[&height=0.256145634688437,length=0.013247518800199032,posterior=1.96
07843137254903E-
5,rate=0.04262653489237]:0.07505239453166723,(55[&height=2.466287417816789E-
7,height_95%_HPD={0.0,5.960464477539062E-7},height_median=2.0116567611694336E-
7,height_range={0.0,3.974884748458862E-
6},length=0.45453179229239027,length_95%_HPD={6.975719770707656E-
7,1.5953375101089478},length_median=0.24092742800712585,length_range={6.975719770707656E-
7,22.774282455444336},rate=0.002496085445468919,rate_95%_HPD={5.264377985856696E-
8,0.00957183292345},rate_median=8.470761225851202E-4,rate_range={5.264377985856696E-
8,0.3268591133674}]:0.1509309932589531,64[&height=2.4707919894797436E-
7,height_95%_HPD={0.0,5.960464477539062E-7},height_median=2.0116567611694336E-
7,height_range={0.0,4.541128873825073E-
6},length=0.4495227017503622,length_95%_HPD={1.1541987078089733E-
5,1.6145446300506592},length_median=0.23264442384243011,length_range={1.1541987078089733E-
5,17.765193939208984},rate=0.002511800457636229,rate_95%_HPD={7.755893874685667E-
9,0.009805005298917},rate_median=8.666496813661735E-4,rate_range={7.755893874685667E-
9,0.2789711722787}]:0.1509309932589531)[&height=0.26521538828639324,height_95%_HPD={6.7609
2691719532E-
4,0.8641967866569757},height_median=0.1509311944246292,height_range={6.76092691719532E-
4,3.5779630728065968},length=0.47772477094769616,length_95%_HPD={1.6824837075546384E-
4,1.602484941482544},length_median=0.2778050899505615,length_range={1.6824837075546384E-
4,5.466706275939941},posterior=0.026803921568627452,rate=0.002290560165265071,rate_95%_HPD
={1.2030760857851804E-6,0.008845394171214},rate_median=8.956889577170962E-
4,rate_range={1.2030760857851804E-
6,0.05113769295505}]:0.180266834795475)[&height=0.3311980292201042,height_95%_HPD={0.26939
3153488636,0.3930029049515724},height_median=0.3311980292201042,height_range={0.269393153
488636,0.3930029049515724},length=0.543020635843277,length_95%_HPD={0.43872666358947754,0
.6473146080970764},length_median=0.543020635843277,length_range={0.43872666358947754,0.647
3146080970764},posterior=3.9215686274509805E-5,rate=9.735767441260076E-
4,rate_95%_HPD={6.527490728910154E-4,0.001294404415361},rate_median=9.735767441260076E-

4,rate_range={6.527490728910154E-
4,0.001294404415361}]:0.37692178785800934,(21[&height=2.4700987365503456E-
7,height_95%_HPD={0.0,5.960464477539062E-7},height_median=2.0116567611694336E-
7,height_range={0.0,4.928559064865112E-
6},length=0.4597772901151325,length_95%_HPD={4.365200766187627E-
6,1.6363686323165894},length_median=0.2395762950181961,length_range={4.365200766187627E-
6,10.077520370483398},rate=0.002400902234783991,rate_95%_HPD={8.813193075573047E-
8,0.00946868992049},rate_median=8.365634194522358E-4,rate_range={8.813193075573047E-
8,0.2263087912712}]:0.4665730665437877,((23[&height=2.468725828200203E-
7,height_95%_HPD={0.0,5.960464477539062E-7},height_median=2.0116567611694336E-
7,height_range={0.0,4.7869980335235596E-
6},length=0.45949424631753927,length_95%_HPD={2.057988695014501E-
6,1.632614254951477},length_median=0.2400948777794838,length_range={2.057988695014501E-
6,18.751859664916992},rate=0.002466112124608511 8,rate_95%_HPD={1.0333949130317864E-
7,0.009994308709788},rate_median=8.523892762437013E-4,rate_range={1.0333949130317864E-
7,0.1712964163318}]:0.14556498173624277,66[&height=2.469200268446421E-
7,height_95%_HPD={0.0,5.960464477539062E-7},height_median=2.0116567611694336E-
7,height_range={0.0,4.7460198402404785E-
6},length=0.46117568593352287,length_95%_HPD={3.4602844607434236E-
6,1.6165924072265625},length_median=0.2403378263115883,length_range={3.4602844607434236E-
6,21.919795989990234},rate=0.0024436497141620667,rate_95%_HPD={4.474498026129309E-
8,0.00959116431431},rate_median=8.692270965872733E-4,rate_range={4.474498026129309E-
8,0.2416454066806}]:0.14556498173624277)[&height=0.25806086510036186,height_95%_HPD={1.860
89426279068E-
4,0.8803442418575287},height_median=0.1455651829019189,height_range={1.86089426279068E-
4,7.13031530380249},length=0.46689070247899145,length_95%_HPD={3.178995393682271E-
5,1.5791802406311035},length_median=0.2764632850885391,length_range={3.178995393682271E-
5,4.510478973388672},posterior=0.025137254901960785,rate=0.0019741225143745282,rate_95%_HP
D={2.4503848247287595E-6,0.007347360950539},rate_median=8.660779084983101E-
4,rate_range={2.4503848247287595E-
6,0.03479344116476}]:0.39657984010409564,((29[&height=2.46237564206641E-
7,height_95%_HPD={0.0,5.960464477539062E-7},height_median=2.0116567611694336E-
7,height_range={0.0,4.7497451305389404E-
6},length=0.4668577820094458,length_95%_HPD={2.936471901193727E-
6,1.667115330696106},length_median=0.23773927241563797,length_range={2.936471901193727E-
6,20.415590286254883},rate=0.0025314721656420043,rate_95%_HPD={7.786535062376834E-
8,0.009587047013878},rate_median=8.634197294041684E-4,rate_range={7.786535062376834E-
8,0.2796623736517}]:0.14661312848329544,35[&height=2.469664029469382E-
7,height_95%_HPD={0.0,5.960464477539062E-7},height_median=2.0116567611694336E-
7,height_range={0.0,4.69014048576355E-
6},length=0.45442158315621894,length_95%_HPD={4.365200766187627E-
6,1.5932884216308594},length_median=0.23678699880838394,length_range={4.365200766187627E-
6,18.66707420349121},rate=0.002483409584709613,rate_95%_HPD={8.958807034509385E-
8,0.009403436068594},rate_median=8.738343825104074E-4,rate_range={8.958807034509385E-

8,0.5326151304579}]:0.14661312848329544)[&height=0.2610262746902433,height_95%_HPD={1.6599
89356994629E-
4,0.9000163804739714},height_median=0.14661332964897156,height_range={1.659989356994629E-
4,4.355910126119852},length=0.5258491356412758,length_95%_HPD={1.7000145453494042E-
4,1.7938803434371948},length_median=0.2858545780181885,length_range={1.7000145453494042E-
4,5.047255992889404},posterior=0.025588235294117648,rate=0.0021128244415500996,rate_95%_HP
D={1.6740569166576988E-6,0.007155371148503},rate_median=8.11671303995523E-
4,rate_range={1.6740569166576988E-
6,0.05789379392902}]:0.06277981954917777,33[&height=2.4712484025880095E-
7,height_95%_HPD={0.0,5.960464477539062E-7},height_median=2.0116567611694336E-
7,height_range={0.0,4.76837158203125E-
6},length=0.46463366593910066,length_95%_HPD={7.265008207468782E-
6,1.630693793296814},length_median=0.24020737409591675,length_range={7.265008207468782E-
6,29.024900436401367},rate=0.002446322368917606,rate_95%_HPD={3.916974113327786E-
8,0.009568877963324},rate_median=8.517618519770628E-4,rate_range={3.916974113327786E-
8,0.2042661418148}]:0.20939294803247321)[&height=0.4014512440713885,height_95%_HPD={0.0065
09169936180115,1.5071647693403065},height_median=0.20939314919814933,height_range={0.0065
09169936180115,3.4578472727444023},length=0.45360263112970456,length_95%_HPD={0.00629576
43531262875,2.010671377182007},length_median=0.19280897825956345,length_range={0.00629576
43531262875,4.860629558563232},posterior=0.0019215686274509803,rate=0.0027845054869593826,
rate_95%_HPD={1.5388961967935548E-6,0.01355815010101},rate_median=9.981857171171516E-
4,rate_range={1.5388961967935548E-
6,0.02918090123408}]:0.3327518738078652)[&height=0.5354219572036527,height_95%_HPD={0.4708
714410662651,0.5865263417363167},height_median=0.5421450230060145,height_range={0.4708714
410662651,0.5865263417363167},length=0.2622970408410765,length_95%_HPD={0.00341619946993
88742,0.7867059111595154},length_median=0.12953302636742592,length_range={0.0034161994699
388742,0.7867059111595154},posterior=7.843137254901961E-
5,rate=0.001382818331190941,rate_95%_HPD={2.2277859180476432E-
4,0.002306602973179},rate_median=0.00150094587989,rate_range={2.2277859180476432E-
4,0.002306602973179}]:-
0.07557175529655069)[&height=0.4756332182441838,height_95%_HPD={0.3045890985522419,0.664
7972390055656},height_median=0.46657326770946383,height_range={0.3045890985522419,0.66479
72390055656},length=0.25277118012309074,length_95%_HPD={0.07261188328266144,0.5468443036
079407},length_median=0.19581426680088043,length_range={0.07261188328266144,0.54684430360
79407},posterior=7.843137254901961E-
5,rate=0.0026661675506957556,rate_95%_HPD={8.250505993659872E-
4,0.005398017254437},rate_median=0.0022208011744900176,rate_range={8.250505993659872E-
4,0.005398017254437}]:0.24154654936864972)[&height=0.7081198170781136,length=0.37911412119
86542,posterior=1.9607843137254903E-5,rate=6.489806590601811E-
4]:0.5035217255353928,(47[&height=2.4685894493275634E-
7,height_95%_HPD={0.0,5.960464477539062E-7},height_median=2.0116567611694336E-
7,height_range={0.0,4.779547452926636E-
6},length=0.4686876225133889,length_95%_HPD={4.912716121907579E-
6,1.6495980024337769},length_median=0.24836276471614838,length_range={4.912716121907579E-

6,12.971847534179688},rate=0.002416663868690158,rate_95%_HPD={8.630036894815499E-
8,0.00919887819382},rate_median=8.43278074612766E-4,rate_range={8.630036894815499E-
8,0.2849663681944}]:0.14485470205545425,61[&height=2.465025564208868E-
7,height_95%_HPD={0.0,5.960464477539062E-7},height_median=2.0116567611694336E-
7,height_range={0.0,4.541128873825073E-
6},length=0.4621621112266026,length_95%_HPD={6.975719770707656E-
7,1.6485975980758667},length_median=0.23705286532640457,length_range={6.975719770707656E-
7,23.261728286743164},rate=0.00241033334889476,rate_95%_HPD={9.215714442099929E-
8,0.009406756998398},rate_median=8.51175113024664E-4,rate_range={9.215714442099929E-
8,0.2061227173005}]:0.14485470205545425)[&height=0.25674680543120976,height_95%_HPD={1.073
0419307947159E-
4,0.9326974898576736},height_median=0.14485490322113037,height_range={1.0730419307947159E-
4,4.010313093662262},length=0.45305474972894183,length_95%_HPD={5.622205208055675E-
4,1.5431259870529175},length_median=0.24998338520526886,length_range={5.622205208055675E-
4,5.189722061157227},posterior=0.025784313725490195,rate=0.0022422627103438767,rate_95%_HP
D={3.965862177086953E-6,0.008157495473432},rate_median=7.879079346264426E-
4,rate_range={3.965862177086953E-
6,0.1174143234386}]:1.066786639392376)[&height=1.2195891787608464,height_95%_HPD={1.087233
9382767677,1.3598920553922653},height_median=1.2116415426135063,height_range={1.087233938
2767677,1.3598920553922653},length=0.16275070110956827,length_95%_HPD={0.100839830935001
37,0.26656609773635864},length_median=0.12084617465734482,length_range={0.100839830935001
37,0.26656609773635864},posterior=5.882352941176471E-
5,rate=0.0029736566360243333,rate_95%_HPD={0.001446407214775,0.005968259075041},rate_medi
an=0.001506303618257,rate_range={0.001446407214775,0.005968259075041}]:0.1214991621673107
1,(((32[&height=2.471658022661992E-7,height_95%_HPD={0.0,5.960464477539062E-
7},height_median=2.0116567611694336E-7,height_range={0.0,4.850327968597412E-
6},length=0.4678333603180374,length_95%_HPD={2.057988695014501E-
6,1.6207506656646729},length_median=0.2393321767449379,length_range={2.057988695014501E-
6,29.618257522583008},rate=0.002412139606588493,rate_95%_HPD={1.521782985895954E-
7,0.009362805977454},rate_median=8.351333098680369E-4,rate_range={1.521782985895954E-
7,0.214940413829}]:0.1364731416106224,45[&height=2.46484508310778E-
7,height_95%_HPD={0.0,5.960464477539062E-7},height_median=2.0116567611694336E-
7,height_range={0.0,4.541128873825073E-
6},length=0.47526717159349247,length_95%_HPD={7.334198016906157E-
6,1.6543002128601074},length_median=0.2450503632426262,length_range={7.334198016906157E-
6,20.450170516967773},rate=0.0023906193264165094,rate_95%_HPD={4.474498026129309E-
8,0.009090705075115},rate_median=8.465686182292773E-4,rate_range={4.474498026129309E-
8,0.2596100113909}]:0.1364731416106224)[&height=0.2476036184074554,height_95%_HPD={2.62372
1957206726E-
5,0.8773507932201028},height_median=0.13647334277629852,height_range={2.623721957206726E-
5,3.773274391889572},length=0.4888086123212585,length_95%_HPD={3.324355639051646E-
4,1.646522045135498},length_median=0.2503637969493866,length_range={3.324355639051646E-
4,7.538283348083496},posterior=0.024843137254901962,rate=0.0023853456279285595,rate_95%_HP
D={4.493433233977143E-7,0.00927760685477},rate_median=8.432869102995178E-

4,rate_range={4.493433233977143E-
7,0.04347168052551}]:0.16077213757671416,59[&height=2.4694451487802967E-
7,height_95%_HPD={0.0,5.960464477539062E-7},height_median=2.0116567611694336E-
7,height_range={0.0,4.541128873825073E-
6},length=0.45373269022489443,length_95%_HPD={1.8922552044386975E-
5,1.5840412378311157},length_median=0.23652155697345734,length_range={1.8922552044386975E-
5,17.22887420654297},rate=0.002393836023201714,rate_95%_HPD={2.916402482267245E-
8,0.009342501508335},rate_median=8.59392417273435E-4,rate_range={2.916402482267245E-
8,0.3623891388586}]:0.29724527918733656)[&height=0.5479517612812356,height_95%_HPD={0.0317
9771825671196,1.9469371438026428},height_median=0.2972454803530127,height_range={0.031797
71825671196,5.04742044210434},length=0.5739222893775066,length_95%_HPD={0.00363916414789
8555,1.6990039348602295},length_median=0.3093110918998718,length_range={0.003639164147898
555,3.1444015502929688},posterior=0.0017058823529411764,rate=0.0020953437270556973,rate_95
%_HPD={2.474490141497925E-6,0.009610202735198},rate_median=8.08660536817661E-
4,rate_range={2.474490141497925E-6,0.02418958448528}]:-
0.041283909464254975,(58[&height=2.469991817690698E-
7,height_95%_HPD={0.0,5.960464477539062E-7},height_median=2.0116567611694336E-
7,height_range={0.0,4.7869980335235596E-
6},length=0.5264187057603297,length_95%_HPD={1.58013281179592E-
5,1.8476873636245728},length_median=0.2781580537557602,length_range={1.58013281179592E-
5,22.473995208740234},rate=0.002806403939364643,rate_95%_HPD={1.4008912115579437E-
7,0.01097081227114},rate_median=9.219528470365547E-4,rate_range={1.4008912115579437E-
7,0.348885603896}]:0.19785551726818085,88[&height=2.4707045023812197E-
7,height_95%_HPD={0.0,5.960464477539062E-7},height_median=2.0131119526922703E-
7,height_range={0.0,4.69014048576355E-
6},length=0.6475182704149977,length_95%_HPD={1.0138661309611052E-
5,2.2358500957489014},length_median=0.3509983718395233,length_range={1.0138661309611052E-
5,15.217195510864258},rate=0.002933664997369608,rate_95%_HPD={2.3887856239110376E-
7,0.01166695110333},rate_median=9.989208684457324E-4,rate_range={2.3887856239110376E-
7,0.283855748224}]:0.1978555171226617)[&height=0.3673351640872942,height_95%_HPD={8.659437
298774719E-
5,1.4006392769515514},height_median=0.19785571843385696,height_range={8.659437298774719E-
5,3.3443821519613266},length=0.5508284840851373,length_95%_HPD={0.001992712961509824,1.99
11975860595703},length_median=0.27553385496139526,length_range={0.001992712961509824,4.53
35516929626465},posterior=0.011705882352941177,rate=0.0038252815664086537,rate_95%_HPD={3.
9853030936140225E-6,0.01093575189674},rate_median=9.361230084324288E-
4,rate_range={3.9853030936140225E-
6,0.2554051226685}]:0.05810585245490074)[&height=0.2559615708887577,height_95%_HPD={0.1668
011099100113,0.3451220318675041},height_median=0.2559615708887577,height_range={0.1668011
099100113,0.3451220318675041},length=1.0771791338920593,length_95%_HPD={1.02127265930175
78,1.1330856084823608},length_median=1.0771791338920593,length_range={1.0212726593017578,1.
1330856084823608},posterior=3.9215686274509805E-
5,rate=0.0032415109174195,rate_95%_HPD={0.002406131268902,0.004076890565937},rate_median=
0.0032415109174195,rate_range={0.002406131268902,0.004076890565937}]:1.0771791338920593)[&

height=1.333140704780817,height_95%_HPD={1.188073769211769,1.478207640349865},height_median=1.333140704780817,height_range={1.188073769211769,1.478207640349865},length=0.14829612523317337,length_95%_HPD={0.13750788569450378,0.15908436477184296},length_median=0.14829612523317337,length_range={0.13750788569450378,0.15908436477184296},posterior=3.9215686274509805E-5,rate=0.003708895844238267 6,rate_95%_HPD={2.864554758465357E-4,0.00713133621263},rate_median=0.0037088958442382676,rate_range={2.864554758465357E-4,0.00713133621263}]:-0.007559049874544144,(16[&height=2.461914921196676E-7,height_95%_HPD={0.0,5.997717380523682E-7},height_median=2.0116567611694336E-7,height_range={0.0,3.814697265625E-6},length=1.5399676160246696,length_95%_HPD={6.360047700582072E-5,6.028118133544922},length_median=0.795756608247757,length_range={6.360047700582072E-5,33.06288528442383},rate=0.004119376846956448,rate_95%_HPD={5.603121378789893E-7,0.01488662829333},rate_median=0.0019458928879585,rate_range={5.603121378789893E-7,0.2308013987037}]:0.9918927326798439,(91[&height=2.46727938953846E-7,height_95%_HPD={0.0,5.960464477539062E-7},height_median=2.0116567611694336E-7,height_range={0.0,4.7497451305389404E-6},length=0.7548326611592076,length_95%_HPD={1.650406738917809E-5,2.6758949756622314},length_median=0.40029922127723694,length_range={1.650406738917809E-5,23.099336624145508},rate=0.003409111470637743 4,rate_95%_HPD={7.882423991802101E-9,0.01351761679253},rate_median=0.0010301283367675001,rate_range={7.882423991802101E-9,0.4383774408147}]:0.18101299554109573,92[&height=2.468329340182012E-7,height_95%_HPD={0.0,5.960464477539062E-7},height_median=2.0116567611694336E-7,height_range={0.0,5.0067901611328125E-6},length=0.5756667939434559,length_95%_HPD={1.0986634151777253E-5,2.0294671058654785},length_median=0.3126141279935837,length_range={1.0986634151777253E-5,11.693586349487305},rate=0.003073799089583239,rate_95%_HPD={9.215714442099929E-8,0.01214093837273},rate_median=9.872599821259403E-4,rate_range={9.215714442099929E-8,0.2719883266362}]:0.18101299554109573)[&height=0.3537020092966373,height_95%_HPD={6.300027016550303E-4,1.1536482572555542},height_median=0.18101319670677185,height_range={6.300027016550303E-4,5.439632501453161},length=0.542202148939715,length_95%_HPD={1.9085596431978047E-4,1.9027718305587769},length_median=0.30969204008579254,length_range={1.9085596431978047E-4,5.266539096832275},posterior=0.018313725490196078,rate=0.0037363170213487147,rate_95%_HPD={6.420444782087745E-8,0.0148472009293},rate_median=0.0010971499091325001,rate_range={6.420444782087745E-8,0.1960220496068}]:0.8108797371387482)[&height=1.14570057453752,height_95%_HPD={0.1729897102341056,2.826882278546691},height_median=0.99189293384552,height_range={0.0296160134021 1928,4.690337557345629},length=0.7443914332007077,length_95%_HPD={0.026436837390065193,1.660356879234314},length_median=0.32145389914512634,length_range={0.026436837390065193,4.24505615234375},posterior=0.0010,rate=0.003485967438472497,rate_95%_HPD={2.8294261177673447E-4,0.01380823779133},rate_median=0.002152701401239,rate_range={1.212385287855738E-4,0.01743535374181}]:0.33368872106075287)[&height=1.3255816549062729,length=0.14963065087795258,posterior=1.9607843137254903E-5,rate=3.896336604676609E-4]:0.14963065087795258,(36[&height=2.471005039622326E-

7,height_95%_HPD={0.0,5.960464477539062E-7},height_median=2.0116567611694336E-
7,height_range={0.0,6.4373016357421875E-
6},length=0.47250603648139855,length_95%_HPD={3.5776631648332113E-
6,1.6583948135375977},length_median=0.24260313808918,length_range={3.5776631648332113E-
6,40.872711181640625},rate=0.0023573804171438694,rate_95%_HPD={1.826460714800456E-
7,0.009181678613539},rate_median=8.444328279921488E-4,rate_range={1.826460714800456E-
7,0.1533541116698}]:0.1861219380516559,62[&height=2.470449337640641E-
7,height_95%_HPD={0.0,5.960464477539062E-7},height_median=2.0116567611694336E-
7,height_range={0.0,3.814697265625E-
6},length=0.5782411732122784,length_95%_HPD={2.844439950422384E-
5,2.025972366333008},length_median=0.3073235750198364,length_range={1.0669716175470967E-
5,19.859954833984375},rate=0.002886787640992095,rate_95%_HPD={1.3913495667950246E-
7,0.01171873315546},rate_median=9.743693434072593E-4,rate_range={7.993374512375265E-
8,0.1991610895471}]:0.1861219380516559)[&height=0.37725039838590135,height_95%_HPD={0.0019
695013761520386,1.4443063586950302},height_median=0.186122139217332,height_range={0.00196
95013761520386,6.909550875425339},length=0.48690485180521803,length_95%_HPD={7.969514263
095334E-
5,1.633428931236267},length_median=0.27326957881450653,length_range={7.969514263095334E-
5,10.178407669067383},posterior=0.01984313725490196,rate=0.002286333507968677,rate_95%_HPD
={1.4409195043464895E-6,0.00949819703992},rate_median=8.642986373515044E-
4,rate_range={1.4409195043464895E-
6,0.04416172592034}]:1.2890901665668935)[&height=1.4752123057842255,length=0.1356022208929
062,posterior=1.9607843137254903E-
5,rate=0.00252746212865]:0.6177590414881706,((34[&height=2.46918125617301E-
7,height_95%_HPD={0.0,5.960464477539062E-7},height_median=2.0116567611694336E-
7,height_range={0.0,4.779547452926636E-
6},length=0.47320690214583283,length_95%_HPD={9.386875717609655E-
6,1.6657464504241943},length_median=0.24595238268375397,length_range={9.386875717609655E-
6,12.83987045288086},rate=0.0023886275140944445,rate_95%_HPD={6.495029820432685E-
8,0.009484768880951},rate_median=8.443993421143841E-4,rate_range={6.495029820432685E-
8,0.2826668303703}]:0.13974260189570487,73[&height=2.470141933402454E-
7,height_95%_HPD={0.0,5.960464477539062E-7},height_median=2.0116567611694336E-
7,height_range={0.0,4.809349775314331E-
6},length=0.4517770396074605,length_95%_HPD={3.928319983970141E-
6,1.601143717765808},length_median=0.23504003882408142,length_range={3.928319983970141E-
6,17.129283905029297},rate=0.0024392303434119123,rate_95%_HPD={9.691527111687408E-
8,0.009366544980172},rate_median=8.467985575509601E-4,rate_range={9.691527111687408E-
8,0.1703178200003}]:0.13974260189570487)[&height=0.24660918936808618,height_95%_HPD={2.066
7316857725382E-
4,0.840629180893302},height_median=0.13974280306138098,height_range={2.0667316857725382E-
4,5.375748872756958},length=0.5000868223972095,length_95%_HPD={1.3616576325148344E-
4,1.6647799015045166},length_median=0.28516511619091034,length_range={1.3616576325148344E-
4,7.533935546875},posterior=0.027137254901960783,rate=0.0020742990164113718,rate_95%_HPD={
8.313510319794195E-7,0.008454776173749},rate_median=7.580447860707177E-

4,rate_range={8.313510319794195E-
7,0.07050868746987}]:0.27688724896870553,(48[&height=2.467990640454053E-
7,height_95%_HPD={0.0,5.960464477539062E-7},height_median=2.0116567611694336E-
7,height_range={0.0,4.7460198402404785E-
6},length=0.45889971958269343,length_95%_HPD={1.8197475583292544E-
5,1.6114836931228638},length_median=0.2417077273130417,length_range={1.8197475583292544E-
5,20.549203872680664},rate=0.002455095323085316\8,rate_95%_HPD={3.622331072556228E-
8,0.009437448605861},rate_median=8.305348830784124E-4,rate_range={3.622331072556228E-
8,0.2509081487658}]:0.1659909151494503,67[&height=2.46990146596808E-
7,height_95%_HPD={0.0,5.960464477539062E-7},height_median=2.0116567611694336E-
7,height_range={0.0,4.291534423828125E-
6},length=0.589929811863388,length_95%_HPD={1.3031896742177196E-
5,2.0508835315704346},length_median=0.3163314312696457,length_range={1.3031896742177196E-
5,16.87615966796875},rate=0.002914447700828803\5,rate_95%_HPD={3.7290997846136166E-
8,0.01145802787927},rate_median=9.687902099234915E-4,rate_range={3.7290997846136166E-
8,0.2352436324799}]:0.1659909151494503)[&height=0.29625927807640884,height_95%_HPD={1.6315
2813911438E-
4,1.0096886206883937},height_median=0.16599111631512642,height_range={1.63152813911438E-
4,3.623031795024872},length=0.4715708870865614,length_95%_HPD={2.1859399566892534E-
4,1.578748345375061},length_median=0.2592345327138901,length_range={2.1859399566892534E-
4,7.47611665725708},posterior=0.014823529411764706,rate=0.0022385418450038145,rate_95%_HPD
={1.967127238725706E-6,0.008364856418001},rate_median=9.062377187255134E-
4,rate_range={1.967127238725706E-
6,0.04429389062409}]:0.2506389357149601)[&height=0.5131774000823498,height_95%_HPD={0.1618
582084774971,1.163035947829485},height_median=0.4166300520300865,height_range={0.16185820
84774971,1.163035947829485},length=0.5890465091913939,length_95%_HPD={0.0332060493528842
9,1.386737585067749},length_median=0.40296487510204315,length_range={0.03320604935288429,1.
386737585067749},posterior=1.96078431372549E-
4,rate=0.003056419698038271,rate_95%_HPD={2.678068397042596E-
4,0.00915053028485},rate_median=9.868637891342117E-4,rate_range={2.678068397042596E-
4,0.00915053028485}]:1.6763412952423096)[&height=2.2622273946053237,height_95%_HPD={0.8631
40344619751,4.059303522109985},height_median=2.092971347272396,height_range={0.8631403446
19751,7.322620630264282},length=1.0708691044080982,length_95%_HPD={0.002995869843289256,2
.774406671524048},length_median=0.7890094816684723,length_range={0.002995869843289256,5.84
0329170227051},posterior=0.0038431372549019606,rate=0.0015785744517806657,rate_95%_HPD={1.
2283727205683873E-5,0.005356598257988},rate_median=6.786957102406937E-
4,rate_range={1.2283727205683873E-
5,0.0378734124242}]:1.3028622642159462)[&height=4.525511248215881,height_95%_HPD={0.942021
269351244,10.62169075012207},height_median=3.3958336114883423,height_range={0.94202126935
1244,14.437042713165283},length=1.2404031540134124,length_95%_HPD={0.004307139199227095,3
.588503837585449},length_median=0.9167692065238953,length_range={0.004307139199227095,8.46
1045265197754},posterior=0.004803921568627451,rate=0.0016107465500733735,rate_95%_HPD={2.3
80609450726871E-6,0.005381644801252},rate_median=9.079807898397754E-
4,rate_range={2.380609450726871E-

6,0.0104641518382}]:1.466126225888729)[&height=5.46003793421487,height_95%_HPD={2.27276244
01450157,10.368938937783241},height_median=4.861959837377071,height_range={2.215803392231
4644,35.284379571676254},length=0.6516446798692661,length_95%_HPD={0.0031240065582096577,
1.9437381029129028},length_median=0.4503672569990158,length_range={0.0031240065582096577,
5.317809581756592},posterior=0.03313725490196078,rate=0.0014121829779374666,rate_95%_HPD={
1.4330207775553088E-6,0.005961667081933},rate_median=5.031464196668214E-
4,rate_range={1.4330207775553088E-
6,0.0274610027916}]:0.4635777547955513)[&height=6.325268401627855,height_95%_HPD={2.332825
779914856,13.260334372520447},height_median=5.325537592172623,height_range={2.07865562289
95323,46.38983345031738},length=2.3574098191367345,length_95%_HPD={0.1363716721534729,5.8
52367877960205},length_median=1.8668732643127441,length_range={0.0075716120190918446,20.3
82049560546875},posterior=0.26925490196078433,rate=0.016062866709515587,rate_95%_HPD={2.35
0791978451269E-
6,0.04400696941883},rate_median=0.011564124075465,rate_range={2.350791978451269E-
6,0.2117021916609}]:4.753721976885572)[&height=11.728435977825397,height_95%_HPD={4.656563
609838486,22.825931611470878},height_median=10.079259569058195,height_range={3.6990283727
645874,124.25080060958862},length=0.0,posterior=1.0,rate=1.0]:0.0;

End;
